# NBLAST: Rapid, Sensitive Comparison of Neuronal Structure and Construction of Neuron Family Databases

**DOI:** 10.1016/j.neuron.2016.06.012

**Published:** 2016-07-20

**Authors:** Marta Costa, James D. Manton, Aaron D. Ostrovsky, Steffen Prohaska, Gregory S.X.E. Jefferis

**Affiliations:** 1 Neurobiology Division, MRC Laboratory of Molecular Biology, Cambridge CB2 0QH, UK; 2 Department of Genetics, University of Cambridge, Cambridge CB2 3EH, UK; 3 Zuse Institute Berlin (ZIB), 14195 Berlin-Dahlem, Germany; 4 Department of Zoology, University of Cambridge, Cambridge CB2 3EJ, UK

**Keywords:** neuroinformatics, NBLAST, neuron similarity, cell type, clustering, online resource

## Abstract

Neural circuit mapping is generating datasets of tens of thousands of labeled neurons. New computational tools are needed to search and organize these data. We present NBLAST, a sensitive and rapid algorithm, for measuring pairwise neuronal similarity. NBLAST considers both position and local geometry, decomposing neurons into short segments; matched segments are scored using a probabilistic scoring matrix defined by statistics of matches and non-matches. We validated NBLAST on a published dataset of 16,129 single *Drosophila* neurons. NBLAST can distinguish neuronal types down to the finest level (single identified neurons) without a priori information. Cluster analysis of extensively studied neuronal classes identified new types and unreported topographical features. Fully automated clustering organized the validation dataset into 1,052 clusters, many of which map onto previously described neuronal types. NBLAST supports additional query types, including searching neurons against transgene expression patterns. Finally, we show that NBLAST is effective with data from other invertebrates and zebrafish.

**Video Abstract:**

## Highlights

• NBLAST is a fast and sensitive algorithm to measure pairwise neuronal similarity
• NBLAST can distinguish neuronal types at the finest level without training
• Automated clustering of 16,129 *Drosophila* neurons identifies 1,052 classes
• Online search tool for databases of single neurons or genetic driver lines

## Teaser

Thousands of single-neuron images are being generated by efforts to map circuits and define neuronal types. Costa et al. validate a new neuronal similarity algorithm, NBLAST, demonstrating that it can distinguish neuronal types and organize huge datasets.

## Introduction

Correlating the functional properties and behavioral relevance of neurons with their cell type is a basic activity in neural circuit research. While there is no universally accepted definition of neuron type, key descriptors include morphology, position within the nervous system, genetic markers, connectivity, and intrinsic electrophysiological signatures (Migliore and Shepherd, 2005, Bota and Swanson, 2007, Rowe and Stone, 1977). Despite this ambiguity, neuron type is a key abstraction, helping to reveal organizational principles and enabling results to be compared and collated across research groups. There is increasing appreciation that highly quantitative approaches are critical to generate cell-type catalogs in support of circuit research (Ascoli et al., 2008, Nelson et al., 2006, Kepecs and Fishell, 2014) (http://acd.od.nih.gov/presentations/brain-interim-report.pdf).

Since neuronal morphology and position strongly constrain connectivity, they have been mainstays of circuit studies for over a century. Classic morphological techniques include the Golgi method used by Cajal, microinjection, and intracellular fills during recording. Recently, genetic approaches to sparse and combinatorial labeling have enabled increasingly large-scale characterization of single-neuron morphology (Jefferis and Livet, 2012).

Classically, the position of neuronal somata or arbors was established relative to anatomical landmarks, revealed by a general counterstain; this is especially effective in brain regions with strong laminar organization, e.g., cerebellum (Cajal, 1911), retina (e.g., Badea and Nathans, 2004, Kong et al., 2005, Sümbül et al., 2014), or fly optic lobe (Fischbach and Dittrich, 1989, Morante and Desplan, 2008). Recently, 3D light microscopy and image registration have enabled direct image fusion to generate digital 3D atlases of brain regions or whole brains (Jefferis et al., 2007, Lin et al., 2007, El Jundi et al., 2010, Rybak et al., 2010, Cachero et al., 2010, Yu et al., 2010b, Sunkin et al., 2013, Zingg et al., 2014, Oh et al., 2014). Atlases can generate specific, testable hypotheses about circuit organization and connectivity at large scales. For example, Chiang et al. (2011) combined genetic mosaic labeling and image registration to produce an atlas of over 16,000 single cells embedded within a standard *Drosophila* brain (FlyCircuit dataset).

Neuronal morphologies can be represented as directed graph structures embedded in 3D space; usually this is the (arbitrary) physical space of the imaging system, rather than a brain atlas. For this reason, databases such as NeuroMorpho.org (Parekh and Ascoli, 2013) contain >37,500 neurons but omit precise positional information. Data on this scale present both an acute challenge, finding and organizing related neurons, but also an opportunity: quantitative morphology may help solve the problem of defining cell type. A key requirement is a tool enabling rapid and sensitive computation of neuronal similarity within and between datasets. This has clear analogies with bioinformatics: the explosion of biological sequence information from the late 1980s motivated the development of sequence similarity tools such as BLAST (Altschul et al., 1990), enabling rapid database queries as well as hierarchically organized protein family databases.

Several strategies for measuring neuronal similarity exist with distinct target applications and different underlying data structures (Cardona et al., 2010, Basu et al., 2011, Mayerich et al., 2012, Ganglberger et al., 2014, Wan et al., 2015). For example, Cardona et al. (2010) developed an elegant approach to match curves in space and validated this on a few hundred traced structures; however, this algorithm treats each unbranched neuronal segment as a separate alignment problem, so there is no natural way to handle trees with many such segments. Recently, Wan et al. (2015) developed a sophisticated approach that combines graph matching with 3D positional information for sensitive global alignment of fully branched neurons, but this carries a significant computational cost (minutes per pair of neurons).

Our own approach started with a simple but flexible representation of neurons as point clouds with vectors defining the local heading of individual processes (Masse et al., 2012). We found that this can be efficiently computed for single-neuron data refractory to automated tracing (Peng et al., 2015), as well as more complex expression patterns. Combining this representation with a very large single-neuron dataset (Chiang et al., 2011) allowed us to validate a new algorithm, NBLAST, that is flexible, extremely sensitive, and very fast (pairwise search times of 2 ms on a laptop). Critically, the algorithm’s scoring parameters are defined statistically rather than by expert intuition, but generalize across neuronal classes.

We first describe the NBLAST algorithm, providing an open source implementation in R and a web query tool. We validate NBLAST for applications including neuron database search, unsupervised clustering, and expression pattern search. NBLAST can identify well-studied neuronal types in *Drosophila* with sensitivity matching domain experts, in a fraction of the time. NBLAST can also identify new neuronal types and reveal undescribed features of topographic organization. Finally, we apply our method to 16,129 neurons from the FlyCircuit dataset, reducing this to a non-redundant set of 1,052 morphological clusters. Manual evaluation of a subset of clusters shows they closely match expert definition of cell types. These clusters, which we organize into an online supercluster hierarchy, represent a preliminary global cell type classification for the *Drosophila* brain.

## Results

### Algorithm

Our goal was to develop a neuron similarity algorithm depending on both spatial location (within a brain or brain region) and branching pattern that was both extremely sensitive and very fast. We envisaged searching large databases of neurons (10,000–100,000 neurons), clustering neurons into families by calculating all-against-all similarity matrices, and efficient navigation of such large datasets. We eventually selected an approach based on direct pairwise comparison of neurons pre-registered to a template brain and represented as vector clouds (further details in Supplemental Information, available online).

**Figure 1:**
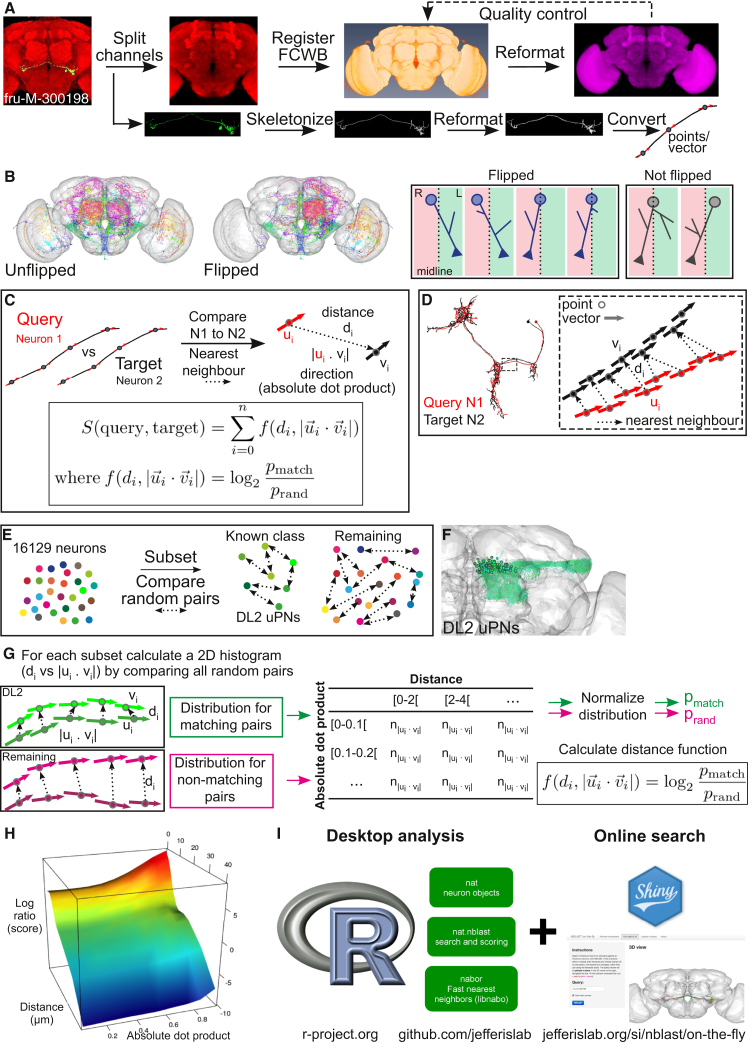
Image Preprocessing, Registration, and NBLAST Algorithm (A) Flowchart describing the image preprocessing and registration procedure. FlyCircuit images were split into two channels. The Dlg-stained brain (Discs large) images were registered against the FCWB template. Successful registrations were applied to neuron skeletons converted into points and vectors. (B) Neurons in the right hemisphere were flipped to the left. Brain plots show 50 random neurons before and after flipping. On the right, cases for which the neuron flipping was assessed manually. (C) NBLAST algorithm. The similarity of two neurons (query and target) is a function of the distance and absolute dot product between nearest-neighbor segments of the query/target pair. This function reflects the probability of a match between a pair of segments (pmatch) relative to a random pair (prand). (D) Diagram illustrating how nearest-neighbor points are calculated. For a query (N1)/target (N2) pair, each point of N1 (u_i_) is matched to a point in N2 (v_i_), minimizing the distance (d_i_). (E) Defining the scoring function. Random pairs of neurons within two groups, DL2 uPNs and all remaining neurons, were compared. (F) Brain plot of DL2 uPNs. (G) Calculation of the distribution for matching and non-matching pairs of segments. For all segment pairs of all neuron pairs in each group, the distance and a 2D histogram were calculated for absolute dot product (10 bins) and distance (21 bins). These histograms were converted to joint probability densities for matching (pmatch) or non-matching pairs (prand) by normalizing the distance histogram to sum to 1. (H) Plot showing that similarity score depends on distance between points and the vector direction (absolute dot product). (I) Summary diagram of the desktop and online NBLAST implementation.

The starting point for our algorithm is to break neurons into short segments, each characterized by a location and tangent vector. This retains local geometry, but not the topology of the neuron’s branching structure. This simplified representation can be constructed for image data that would not permit automated reconstructions. To prepare such data in quantity, we developed an image processing pipeline summarized in Figure 1A (see Experimental Procedures). Briefly, brain images from the FlyCircuit dataset (Chiang et al., 2011) were subjected to non-rigid image registration (Jefferis et al., 2007) to a new intersex template brain. Neuron images were thresholded and skeletonized (Lee et al., 1994) using Fiji (Schindelin et al., 2012), thresholded images were converted to the point and tangent vector (i.e., local heading) representation (Masse et al., 2012) using our R package nat (Jefferis and Manton, 2014), and tangent vectors were computed as the first eigenvector of a singular value decomposition (SVD) of each point and its five nearest neighbors.

After preprocessing, 3D data were visualized and analyzed in R (Figure 1B). Neurons had a median of 1,070 points/vectors; the 16,129 neurons occupied 1.8 GB, fitting comfortably in a laptop’s main memory. Since the fly brain is almost completely symmetric, we mapped all neurons to the left hemisphere (defined primarily by cell body location; see Experimental Procedures and Figure 1B) using a non-rigid mirroring procedure (Manton et al., 2014).

We then calculated NBLAST pairwise similarity scores using this database of preprocessed, aligned neurons. For a given query and target neuron, we iterate over each segment in the query neuron, identifying the nearest neighbor (Euclidean distance) in the target neuron (Figures 1C and 1D). The score for the segment pair is a function of two measurements: $d_{i}$, the distance between matched segments (indexed by *i*), and $\left|\vec{u_{i}}\cdot \vec{v_{i}}\right|$, the absolute dot product of the two tangent vectors; the absolute dot product is used because the head-to-tail orientation of tangent vectors is arbitrary (Figure 1C). The scores are then summed over each segment pair to give a raw score, *S*:

(Equation 1) $$S\left(\text{query},\text{target}\right)=\sum_{i=1}^{n}f\left(d_{i},\left|\vec{u_{i}}\cdot \vec{v_{i}}\right|\right).$$

The next question is, what is an appropriate function $f\left(d_{i},\left|\vec{u_{i}}\cdot \vec{v_{i}}\right|\right)$? Our approach was inspired by the BLAST scoring system (Altschul et al., 1990). For each segment pair, we defined the score as the log probability ratio,

(Equation 2) $$f={\text{log}}_{2}\frac{p_{\text{match}}}{p_{\text{rand}}},$$

i.e., the probability that the segment pair was derived from a pair of neurons of the same type, versus a pair of unrelated neurons. We then defined $p_{\text{match}}$ empirically using the joint distribution of *d* and $\left|\vec{u_{i}}\cdot \vec{v_{i}}\right|$ for pairs of neurons of the same type (Figures 1E–1G); we used 150 olfactory projection neurons (PNs) innervating the same glomerulus, therefore unambiguously the same neuronal type (Figure 1F). $p_{\text{rand}}$ was calculated by drawing 5,000 random pairs of neurons from the database, assuming that the large majority of such pairs are unrelated neurons. Joint distributions were calculated for both groups and normalized to convert them to probabilities, and the log ratio defined the final scoring matrix (Figure 1G). Plotting the scoring matrix emphasizes the strong distance dependence of the score but also shows that for segment pairs closer than ∼10 μm, the logarithm of the odds score increases markedly as the absolute dot product moves from 0 to 1 (Figure 1H).

**Figure 2:**
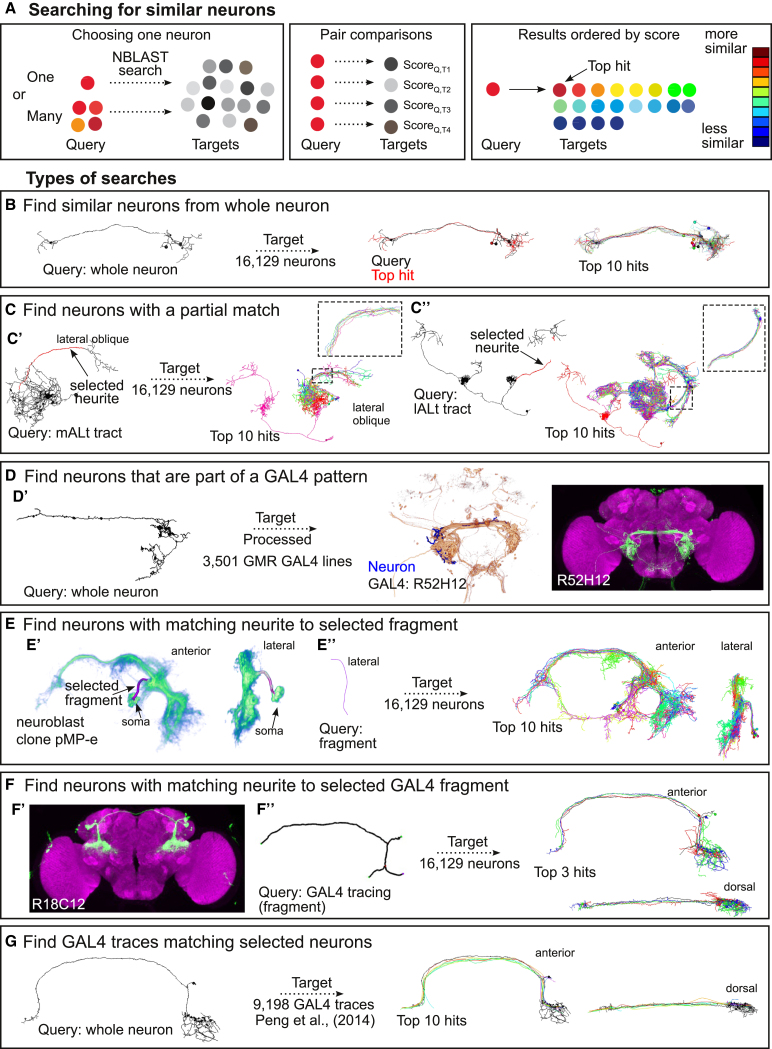
NBLAST Allows Different Search Types (A) Searching for neurons with NBLAST. Pairwise scores between a query and target neurons produce a ranked result set. (B) NBLAST search using a whole query neuron against the FlyCircuit dataset. The query (fru-M-400121), top hit, and top ten hits in anterior view. (C) NBLAST search using a neuron fragment against the FlyCircuit dataset. The query and top ten hits are shown. (C′) Search with the mALT tract from an olfactory PN (Cha-F-000239). Lateral oblique view; inset shows mALT tract for top ten hits. (C′′) Search with lALT tract from an olfactory PN (Gad1-F-200095). Anterior view; inset shows lALT tract of top ten hits. (D) NBLAST search of neuron against FlyLight GAL4 lines. (D′) From left to right: query neuron (Trh-M-300069), volume rendering of query and best GAL4 hit (R52H12), and maximum Z projection of hit. (E) NBLAST search for FlyCircuit neurons matching a fragment from a *fruitless* neuroblast clone (pMP-e). (E′) Volume rendering of pMP-e clone with the traced fragment (anterior and lateral views). (E′′) Query fragment in lateral view. Top ten hits in anterior and lateral view. (F) NBLAST search for FlyCircuit neurons matching fragment traced from GAL4 image (R18C12) (Jenett et al., 2012). (F′) Maximum Z projection of line R18C12. (F′′) The query fragment traced in Vaa3D (anterior view). Top three hits (anterior and dorsal views). (G) GAL4 traces (Peng et al., 2014) matching selected FlyCircuit neuron (VGlut-F-500818). The top ten trace hits are shown (anterior and dorsal views).

We implemented the NBLAST algorithm as an R package (nat.nblast), building on a high-performance k-nearest neighbor library (nabor), that immediately enables pairwise queries, searches of a single query neuron against a database of target neurons (Figure 2), and all-by-all searches (Figure 1I). Runtimes on a single core laptop computer were 2 ms per comparison or 30 s for all 16,129 neurons. In order to enable interactive neuron clustering, we also pre-computed an all-by-all similarity matrix for all 16,129 neurons (2.6 × 10^8^ scores, 1.0 GB). We also developed a simple web application (see jefferislab.org/si/nblast), enabling online queries of this test dataset (Figure 1I).

### NBLAST Finds Whole or Partial Matches for Diverse Query Objects

NBLAST is flexible, identifying both global and partial matches for multiple classes of queries (Figure 2). The only requirements are that these objects (or fragments) must be registered against a template brain and converted to a point and vector representation.

Our first example queries a (whole) FlyCircuit neuron against 16,129 FlyCircuit neurons. The top hits are very similar neurons with small differences in length and neurite position (Figure 2B). Using this search type, we identified Kenyon cell (KC), olfactory projection, and auditory neuron classes and subclasses, known and new (Figures 4 and S6). A second example uses an axon fragment; all top hits follow the same axon tract, but their variable axonal and dendritic arbors define distinct neuron types (Figure 2C). NBLAST searches using a neuron fragment could identify known and new visual projection and mAL neuron types (Figures 5, S5, and 6). In a third example, we query against 3,501 FlyLight GAL4 driver lines (Jenett et al., 2012), finding lines that contain the query neuron (Figure 2D).

User tracings can also be used as queries. We traced the characteristic bundle of 20–30 primary neurites of the *fruitless* neuroblast clone pMP-e that generates male-specific P1 neurons (Kimura et al., 2008, Cachero et al., 2010). This returned many P1 neurons (Figure 2E), identifying new subtypes likely to have distinct functions in male behavior (Figure 6). A similar approach can be used to identify candidate neuronal types labeled by genetic driver lines even when the detailed morphology of individual neurons cannot be determined: we traced the main neurites of a cell cluster in a GAL4 line (Jenett et al., 2012) (Figure 2F) and used that trace as the query. NBLAST identified three very similar FlyCircuit neurons, which completely overlapped with the GAL4 expression pattern. These three neurons appear to be different subtypes, each varying in their terminal arborizations. Conversely, we used one tracing from a published projectome dataset containing >9,000 neurite fibers (Peng et al., 2014) to find similar FlyCircuit neurons (Figure 2G).

### NBLAST Scores Are Sensitive and Biologically Meaningful

A good similarity algorithm should be sensitive enough to reveal identical neurons with certainty, while having the specificity to ensure that all high-scoring results are relevant. We used the full FlyCircuit dataset to validate NBLAST performance.

**Figure 3:**
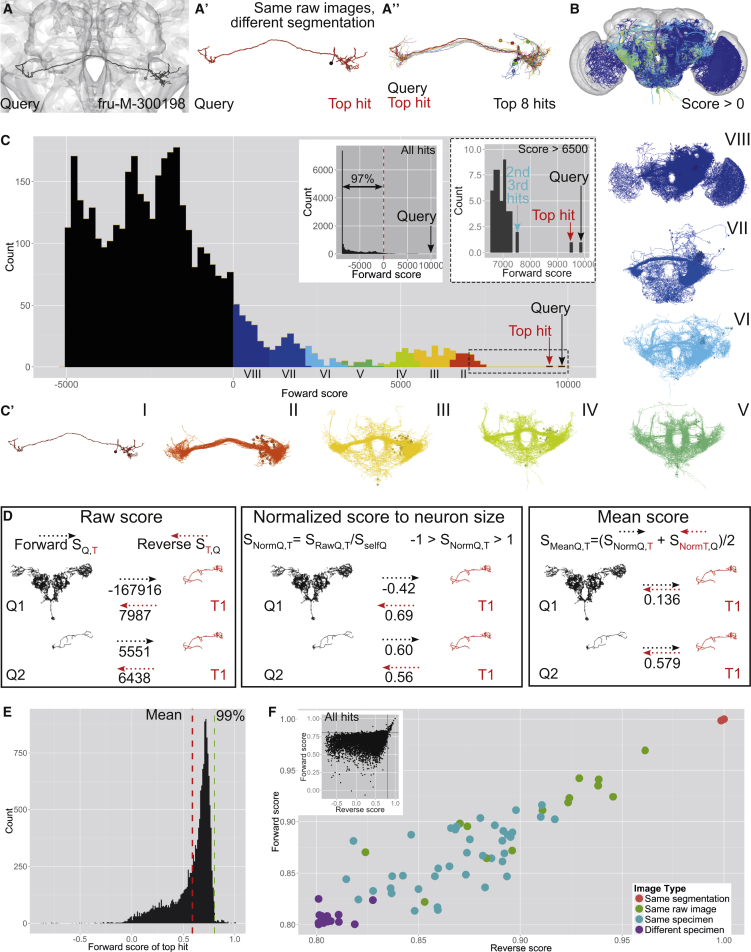
NBLAST Scores Are Accurate and Meaningful (A) NBLAST search with fru-M-300198 (black). (A′) Query neuron (black) and top hit (red). The top hit is a different segmentation of the same raw confocal image. (A′′) Top eight hits have differences in neurite branching, length, and position. (B) All hits with forward score >0, colored by score, as shown in (C). (C) Histogram of forward scores for fru-M-300198. Only hits with scores >−5,000 are shown. Left inset shows score histogram for all hits; right inset shows zoomed view of top hits (score > 6,500). See also Figure S1. (C′) Neurons in each of the score bins in (C). (D) Comparison of raw, normalized, and mean score for two pairs of neurons: one of unequal (Q1, T1) and one of similar size (Q2, T1). (E) Histogram of normalized top scores for each neuron in the whole dataset. The mean and 99th percentile are shown as dashed red and green lines, respectively. (F) Plot of normalized reverse and forward scores for 72 pairs of neurons exceeding threshold score of 0.8. These pairs were classified into four categories of decreasing predicted similarity: same segmentation, same raw image, same specimen, and different specimen. Inset shows normalized reverse and forward scores for all top hits with threshold of 0.8 indicated by two black lines.

Our first example uses an auditory interneuron, fru-M-300198, as query (Figures 3A–3C). The highest NBLAST score was the query neuron itself (it is present in the database), followed by the top hit (fru-M-300174), which completely overlaps with the query (Figure 3A′). A histogram of NBLAST scores showed that the top hit was clearly an outlier, scoring 96.1% compared to the self-match score of the query neuron (Figure 3C). Further investigation revealed that these “identical twins,” both derived from the same raw confocal image, were likely the result of a data entry error. The next eight hits are also very similar to the query but are clearly distinct specimens, having small differences in position, length, and neurite branching that are typical of sister neurons of the same type (Figure 3A′′).

The score histogram shows that only a minority of hits (3%) have a score above 0 (Figures 3B and 3C). A score of 0 represents a natural cutoff for NBLAST, since it means that, on average, segment pairs from this query and target neuron have a similarity level that is equally likely to have arisen from a random pair of neurons in the database as a pair of neurons of the same type. We divided the neurons with score >0 into 8 groups with decreasing similarity scores (Figure 3C′). Only the highest-scoring real hits (group II) appear to be of exactly the same type, although lower-scoring groups contain neurons that would be ranked as very similar.

Although raw NBLAST scores correctly identify similar neurons, they are not comparable from one query neuron to the next: the score depends on neuron size and segment number. This confounds search results for neurons of very different sizes or when the identity of query and target neurons is reversed. For example, a search with a large neuron as query and a smaller one as target (pair 1) will have a very low forward score because the large neuron has many unmatched segments, but a high reverse score, since most of target will match part of the query (Figure 3D). One approach to correct for this is to normalize the scores by the size of the query neuron. Although normalized scores are comparable, unequal forward and reverse scores between large and small neurons remain an issue. One simple strategy is to calculate the mean of the forward and reverse scores (mean score). Two neurons of similar size have a higher mean score than two neurons of unequal size (Figure 3D). Repeating the analysis of Figures 3C and 3C′ using mean scores (Figure S2) eliminated some false matches due to unequal size.

During our analysis, we sporadically noticed cases where two database images were derived from the same physical specimen (Figure S1). We tested if NBLAST could identify these instances. We collected the top hit for each neuron and analyzed the distribution of forward (Figure 3E) and reverse scores (data not shown). A small tail (∼1% of all top hits) has anomalously high scores (>0.8). Given this distribution, we examined neuron pairs with forward and reverse scores >0.8. We classified these 72 pairs into 4 different groups. From highest to lowest predicted similarity, the groups are as follows: same segmentation, i.e., a neuron image duplicated after segmentation (Figure S1A); same raw image, resulting in different segmentations of the same neuron (Figure 3B′); same specimen, i.e., two separate confocal images from the same brain (Figure S1B); and different specimen, when two neurons are actually from different brains (but of the same neuron type). The distribution of NBLAST scores for these four categories matches the predicted hierarchy of similarity (Figure 3F). These results underline the high sensitivity of the NBLAST algorithm to small differences between neurons.

Taken together, these results validate NBLAST as a sensitive and specific tool for finding similar neurons.

### NBLAST Scores Can Distinguish Kenyon Cell Classes

We next investigated whether NBLAST scores can be used to cluster neurons, potentially revealing functional classes. We began with KCs, the intrinsic neurons of the mushroom body and an intensively studied population given their key role in memory formation and retrieval (reviewed in Kahsai and Zars, 2011).

**Figure 4:**
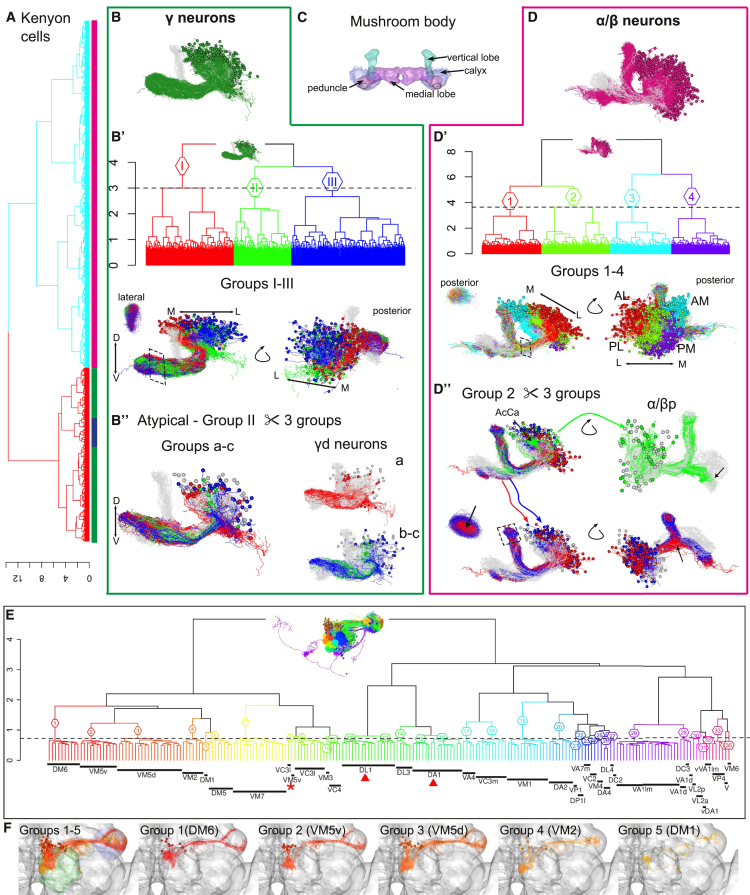
NBLAST Search and Clustering Reveal Kenyon Cell Subtypes (A) Hierarchical clustering (HC) of KCs (n = 1,664). Bars below the dendrogram indicate the γ (green), α′/β′ (blue), and α/β neurons (magenta); h = 8.9. (B) Plot of all γ neurons. KC exemplars plotted in gray for context. (B′) HC of γ neurons (I–III); h = 3. Neuron plots of groups I–III. Lateral oblique and posterior views of neurons and lateral view of slice through horizontal lobe. (B′′) HC of atypical γ neurons (group II in B′) divided into three groups (a–c). Neuron plots of groups a–c, a, and b and c. Group a corresponds to the γd subtype. See also Figure S3. (C) Mushroom body neuropil and subregions. (D) Neuron plot of α/β neurons. KC exemplars plotted in gray for context. (D′) HC of α/β neurons divided into four groups (1–4); h = 3.64. Neuron plots of groups 1–4, which match neuroblast clones AM, AL, PM, and PL in posterior and lateral oblique views. (D′′) HC of group 2 divided into three subgroups. Lateral oblique, posterior oblique, and dorsal view of a peduncle slice are shown. Red and blue subgroups match core and surface neurons, respectively; green subgroup corresponds to α/β posterior subtype (α/βp) (see also Figure S3D). AcCa, accessory calyx. (E) Hierarchical clustering of uPNs (non-DL2s) (n = 214) cut into 35 groups (1–35) at h = 0.725. Dendrogram shows glomerulus for each neuron. Inset shows uPNs colored by dendrogram group. Neurons that innervate each glomerulus are indicated by black rectangles under dendrogram. Neurons originating from ventral neuroblast are indicated as vVA1lm and vDA1. Dendrogram groups correspond to unique neuron types, except for DL1 and DA1 neurons, which are split into two groups (12–13, 15–16, respectively) (red arrowhead), and the outlier neuron VM5v in group 9 (red asterisk). (F) Neurons for groups 1–5 from (E); antennal lobe in green; lateral horn in purple. See also Figure S4.

There are around 2,000 KCs in each mushroom body (Aso et al., 2009), whose axons form the medial lobe, consisting of the γ, β′, and β lobes, and the vertical lobe, consisting of the α and α′ lobes. The dendrites form the calyx around which cell bodies are positioned; the axon peduncle joins the calyx to the lobes (Figure 4C). Three main classes of KCs are recognized, named by the lobes they innervate: γ neurons are the first born, α′/β′ neurons are generated next, and last born are α/β neurons. Four neuroblasts each generate the whole repertoire of KC types (Lee et al., 1999).

We started with a dataset of 1,664 KCs, representing 10.3% of the FlyCircuit dataset (see Supplemental Information for selection protocol), and calculated raw NBLAST scores of each KC against all others. Iterative hierarchical clustering allowed us to identify the main KC types, followed by detailed analyses that distinguished several subtypes.

For γ neurons (Figure 4B), we identified the classical neurons (Figure 4B′) (groups I and III), the recently described γd neurons (group a) (Aso et al., 2009, Aso et al., 2014), and two previously uncharacterized types (groups b and c) (Figure 4B′′). Analysis of α′/β′ neurons highlighted the characterized subtypes of these neurons (Figure S3C), which differ in their anterior/posterior position in the peduncle and β′ lobe (Tanaka et al., 2008, Aso et al., 2014).

The largest KC subset corresponds to α/β neurons (Figure 4D). We identified neurons from each of the four neuroblast lineages (Figure 4D′) (Zhu et al., 2003), and for each of these, we distinguished morphological subtypes that correlate to their birth time (Figures 4D′′ and S3D′): the last born (α/β core) inside the α lobe, the earlier (α/β) surface layer, and the earliest born (α/β posterior or pioneer) (Tanaka et al., 2008).

Hierarchical clustering of KCs using NBLAST scores therefore resolved KCs into three main types, identified the reported subtypes, and even isolated uncharacterized subtypes in an intensively studied cell population. This supports our claim that the NBLAST scores are a good metric when searching for similar neurons and organizing large datasets of related cells.

### NBLAST Identifies Classic Cell Types at the Finest Level: Olfactory PNs

We have shown that clustering NBLAST scores can identify KC types. However, it remains uncertain what corresponds to an identified cell type, which we take to be the finest neuronal classification in the brain. We therefore analyzed a different neuron family, the olfactory PNs, which represent one of the best-defined cell types in the fly brain.

PNs transmit information between antennal lobe glomeruli, which receive olfactory input, and higher brain centers, including the mushroom body and the lateral horn (Masse et al., 2009). Uniglomerular PNs (uPNs) are unambiguously classified into individual types based on the glomerulus innervated by their dendrites and the axon tract they follow; these features show fixed relationships with their axonal branching patterns in higher centers and their parental neuroblast (Marin et al., 2002, Jefferis et al., 2001, Jefferis et al., 2007, Wong et al., 2002, Yu et al., 2010a, Tanaka et al., 2012).

We manually classified the 400 FlyCircuit uPNs by glomerulus (see Experimental Procedures). We found a very large number of DL2 uPNs (145 DL2d and 37 DL2v), out of 397 classified neurons. Nevertheless, our final set of uPNs broadly represents the total variability of described classes and contains neurons innervating 35 out of 56 different glomeruli (Tanaka et al., 2012), as well as examples of the three main lineage clones and tracts.

We computed mean NBLAST scores for each uPN versus the other 16,128 neurons, checking if the top hit was exactly the same uPN type, another uPN type, or a match to a different neuron class (Figure S4A). There were only eight cases in which the top hit did not match the query’s type. These matches represented cases of uPNs innervating a neighboring glomerulus or multiglomerular PNs. This exercise encapsulates a very simple form of supervised learning (k-nearest neighbor with k = 1 and leave-one-out cross-validation) and shows that NBLAST scores are a useful metric, with an error rate of 2.4% for 35 classes; it is noteworthy that there was a huge amount of distracting information since uPNs represented only 2.47% of the 16,128 test neurons.

We also compared how the top three hits matched the query type (Figure S4B). For uPN types with more than three examples (non-DL2, n = 187), we collected the top three NBLAST hits for each of these neurons. We achieved very high matching rates: in 98.9% of cases (i.e., all but two), at least one of the top hits matched the query type, and all three hits matched the query type in 95.2% of cases.

Given the very high prediction accuracy, we wondered if unsupervised clustering based on NBLAST scores would group uPNs by type. To test this, we clustered uPNs (non-DL2, n = 214) and cut the dendrogram at a height of 0.725: at this level most groups corresponded to single-neuron types. For types with more than one representative neuron, all neurons co-clustered, with three exceptions (Figures 4E, 4F, and S4). The cluster organization also reflects higher-level features such as the axon tract/neuroblast of origin. Thus, unsupervised clustering of uPNs based on NBLAST scores gives an almost perfect neuronal classification: our two expert annotators took three iterative rounds of consensus-driven manual annotation to better this error rate of 1.4%.

In conclusion, these results demonstrate that morphological comparison by NBLAST is powerful enough to resolve differences at the finest level of neuronal classification. Furthermore, they suggest that unsupervised NBLAST clustering could help reveal new neuronal types.

### NBLAST Can Define New Cell Types

**Figure 5:**
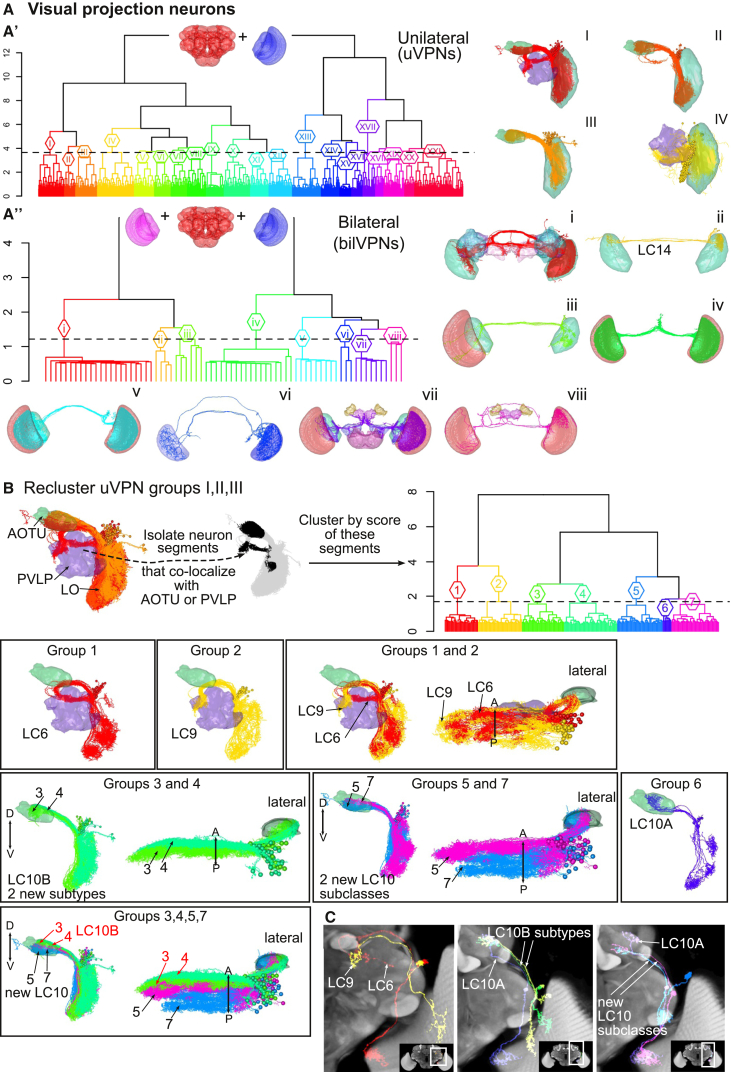
NBLAST Classification of Visual PNs (A) Clustering of unilateral (uVPNs) and bilateral visual PNs (bilVPNs). Inset shows neuropils to which NBLAST was restricted. At right, plots of neuron groups, showing neuropils with most overlap. See also Figure S5. (A′) Hierarchical clustering (HC) of uVPNs divided into 21 groups (I–XXI); h = 3.65. (A′′) HC of bilVPNs divided into 8 groups (i–viii); h = 1.22. Group ii corresponds to the LC14 neuron type (Otsuna and Ito, 2006). (B) Reclustering of uVPN groups I, II, and III from A′. Only neuron segments within anterior optic tubercle (AOTU) or posterior ventrolateral protocerebrum (PVLP) were used for NBLAST HC. The dendrogram was cut into groups 1–7; h = 1.69. Neuron plots match dendrogram groups to known uVPN types. Group 1, LC6 neurons; group 2, LC9. Groups 3 and 4, two new LC10B subtypes. Groups 5 and 7, possible new LC10 types. Group 6, LC10A neurons. This analysis identified five subgroups of LC10 neurons, four of them not previously identified (see also Table S1 and Figure S5B). (C) Plots of neuron skeletons with partial confocal image Z projections for selected types. White rectangle in inset shows location of close-up. LC, lobula columnar neuron.

We wished to show the usefulness of whole and partial NBLAST searches in classifying other well-studied neuron types, and especially in identifying new cell types. We analyzed the visual PNs (VPNs), which relay information between optic lobe and the central brain (Figures 5 and S5). This is a morphologically diverse group with 44 types already described (Otsuna and Ito, 2006). We clustered FlyCircuit VPNs based only on the parts of their skeletons that overlap the central brain neuropils; this identified 11 known VPN types, 3 new subclasses, and 4 subtypes of unilateral VPNs (Table S1).

Another large and diverse neuron group is the auditory neurons. Several distinct types have been described based on anatomical and physiological features (Yorozu et al., 2009, Lai et al., 2012, Kamikouchi et al., 2006, Kamikouchi et al., 2009, Matsuo et al., 2016). Using simple whole-neuron searches, we were able to reveal new subtypes that differed mainly in their lateral arborizations (Figure S6; Table S2).

**Figure 6:**
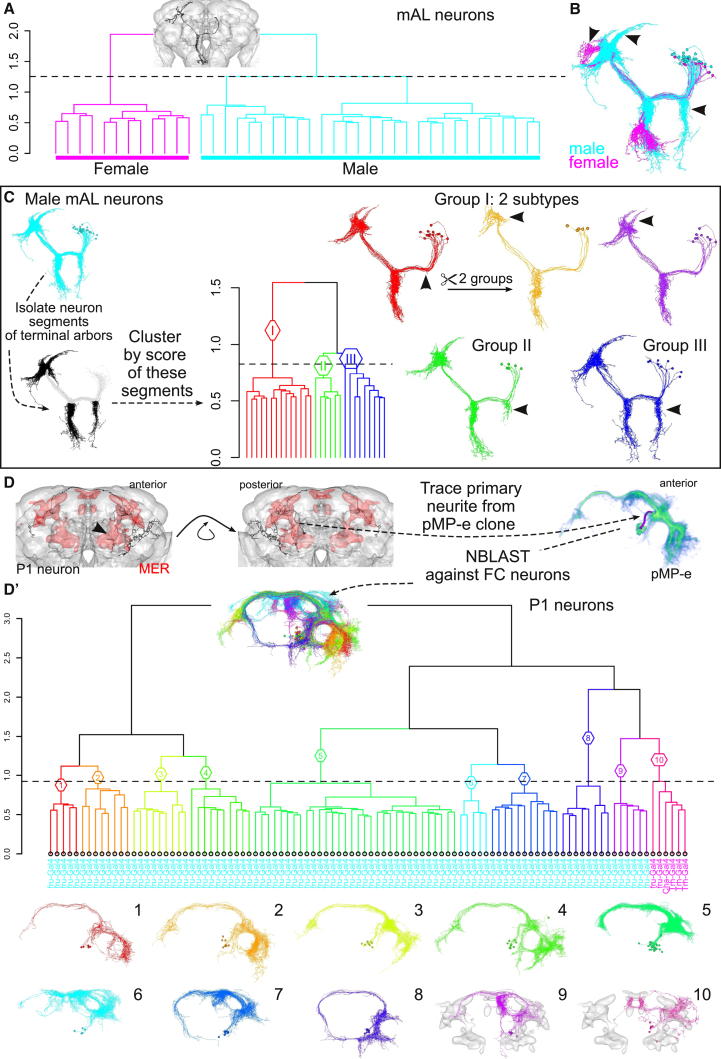
NBLAST Classification of Sexually Dimorphic Neurons (A) *fruitless* mAL neurons. Hierarchical clustering (HC) of hits cut into two groups (h = 1.25). Inset shows mAL query neuron (fru-M-500159). Leaves labeled with sex of neuron: female or male. (B) Neurons from two dendrogram groups: male (cyan) and female (magenta). (C) Analysis of male mAL neurons. Neuron segments for terminal arbors (ipsi- and contralateral) were isolated and NBLAST scores calculated. HC divided into three groups, I–III (h = 0.83). Arborization differences indicated by arrowheads. Group I (red) is further subdivided in two. (D) *fruitless* P1 neurons. Plot of query neuron (fru-M-400046). Male enlarged brain region (MER) shown in red (anterior and posterior views). Volume rendering of pMP-e *fruitless* neuroblast clone containing P1 neurons. The distinctive primary neurite of pMP-e was traced. (D′) HC of NBLAST hits for P1 trace divided into groups 1–10 (h = 0.92). Inset shows neurons colored by group. Leaves labeled by GAL4 driver used to obtain neuron, colored cyan (male) and magenta (female). Below dendrogram, neuron plots of each group; MER shown in gray for groups 9 and 10.

We also studied two classes of *fruitless*-expressing, sexually dimorphic neurons, critical for courtship behavior, the mAL (Koganezawa et al., 2010, Kimura et al., 2005) and P1 neurons (Kimura et al., 2008). We calculated NBLAST scores for partial mAL skeletons containing their axonal and dendritic arbors, clustering cleanly separated male and female neurons (Figures 6A and 6B; Supplemental Experimental Procedures), and identified three main types and two subtypes for the male neurons (Figure 6C). These male neurons include types with correlated differences in the position of input and output arbors (and likely therefore in functional connectivity). Clustering P1 neurons identified ten anatomical subtypes (Figure 6D). Nine of these contained only male neurons, each with highly distinctive patterns of dendritic and axonal arborization, suggesting that they are likely to integrate distinct sensory inputs and connect with distinct downstream targets. The last group consists only of female neurons, suggesting that a small population of female neurons shares anatomical features (and likely originates from the same neuroblast) with the male P1 neurons, key regulators of male behavior.

These analyses demonstrate that NBLAST scores for whole neurons or subregions can highlight morphological features important for defining neuron classes and provide an efficient and quantitative way to identify new cell types even for intensively studied neuronal classes.

### Superclusters and Exemplars to Organize Huge Data

**Figure 7:**
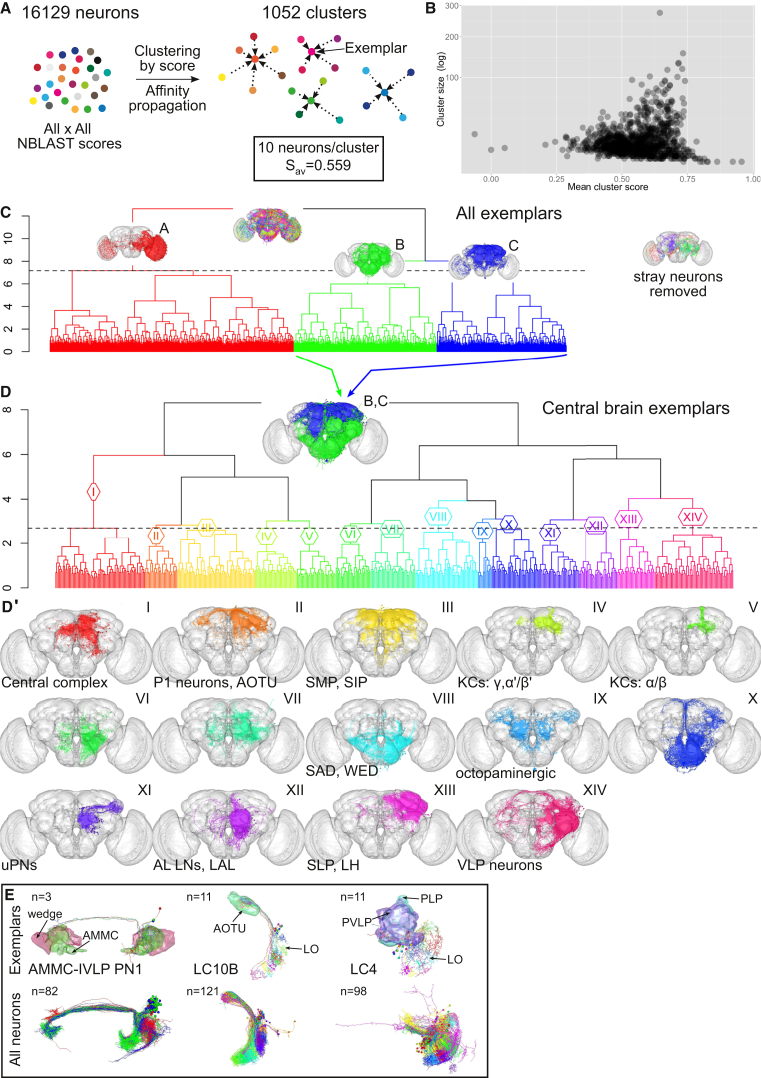
Organizing Huge Neuron Datasets (A) Affinity propagation generated 1,052 clusters. The mean within-cluster similarity score was 0.559; mean cluster size = 10. Exemplars were identified for each cluster. (B) Mean cluster score versus cluster size. (C) Hierarchical clustering (HC) of 1,052 exemplars divided into three groups (A–C). Group A corresponds mostly to optic lobe and VPN neurons; B and C to central brain neurons. Insets show neurons from these groups. Right inset shows stray neurons removed (n = 11). (D) HC of central brain exemplars (groups B and C, inset) cut into 14 groups; h = 2.7. (D′) Neuron plots from dendrogram groups in (D). Main neuron types/innervated neuropils are noted. (E) Affinity propagation of defined neuron types. Exemplar neurons (top row) or all neurons (bottom row) for auditory AMMC-IVLP PN1 neurons (compare Figure S6D) and VPN types LC10B (compare Figure 5B) and LC4 (compare Figure S5B). Numbers of exemplars and neurons indicated in top left corner. AMMC (antennal mechanosensory and motor center) in green; wedge in magenta; AOTU (anterior optic tubercle) in green; LO, lobula; PVLP (posterior ventrolateral protocerebrum) in purple; PLP (posterior lateral protocerebrum) in cyan.

We have shown that NBLAST clustering can identify known and novel neuron types starting from a collection of neurons of a particular superclass (e.g., olfactory PNs). However, isolating such neuronal subsets requires considerable time. We next established a method to organize large datasets, extracting the main types automatically, retaining information on the similarity between types and subtypes, and allowing quicker navigation. We used affinity propagation clustering (Frey and Dueck, 2007), combined with hierarchical clustering, to achieve this. Applying affinity propagation to the 16,129 neurons in the FlyCircuit dataset resulted in 1,052 clusters (Figures 7A and 7B), each characterized by a single exemplar neuron. Hierarchical clustering of the exemplars and manually removing eleven stray neurons isolated the central brain neurons (groups B and C) (Figure 7C). Further hierarchical clustering of central brain exemplars revealed large superclasses of neuron types (groups I–XIV), with most containing an anatomically distinct subset, e.g., central complex neurons (I), P1 neurons (II), KCs (IV and V), and auditory neurons (VIII) (Figures 7D and 7D′). There were, however, superclusters for which the classification logic was not as clear (XI and XII, for example).

The affinity propagation clusters are also useful for identifying neuronal subtypes by comparing all clusters that contain a specified neuronal type (Figure 7E). We present examples for the neuronal types AMMC-IVLP PN 1 (AMMC-IVLP PN1) (Lai et al., 2012), and the uVPNs LC10B and LC4. For each of these, morphological differences are clear between clusters, suggesting that each one might help to identify distinct subtypes.

In short, combining affinity propagation with hierarchical clustering is an effective way to organize and explore large datasets, condensing information into a single exemplar, while retaining the ability to move up or down in the hierarchical tree, revealing broader superclasses or more narrow subtypes.

### NBLAST Extensions

**Figure 8:**
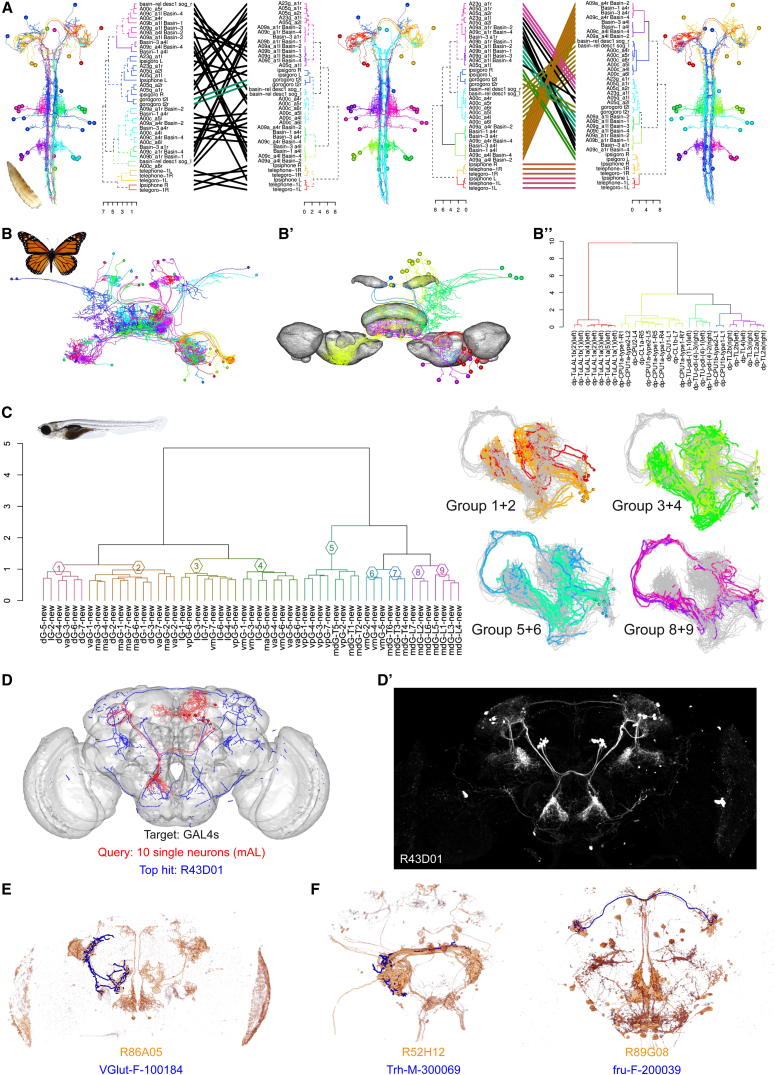
NBLAST extensions (A) Larval *Drosophila* EM tracings. Tanglegrams match two different dendrograms; neurons are plotted by dendrogram group. In the middle, the original neurons; on the left, clustering based on synaptic connectivity network; on the right, NBLAST clustering after pruning first- and second-order terminal branches from neurons. Left tanglegram compares network clustering to NBLAST clustering; right tanglegram compares NBLAST clustering of original and pruned neurons. (B) Monarch butterfly central complex neurons. (B′) Neurons after mirroring and clustering colored by dendrogram group. Some brain neuropils are shown in gray. (B′′) Hierarchical clustering after mirroring neurons. (C) Zebrafish mitral neurons. Left: hierarchical clustering after mirroring neurons. Right: four examples of pairwise comparisons of neuron groups, colored by dendrogram group. (D) Vector cloud representation of R43D01 expression pattern (blue) and query FlyCircuit mAL neurons (red). (D′) R43D01 expression pattern. (E) Candidate V-glomerulus-selective GAL4 expression pattern with query neuron (VGlut-F-100184). (F) Identified GAL4 expression patterns found with AMMC-AMMC PN1 auditory neuron (Trh-M-300069) and AOTUv2 lineage neuron (fru-F-200039).

NBLAST is a powerful tool for working with single neurons from the adult fly; however, the algorithm was designed to be general. We now illustrate NBLAST in a wide variety of experimental contexts. We first use 40 neurons reconstructed from a complete serial section electron microscopy (EM) volume of the *Drosophila* larva. Clustering NBLAST scores recovers functional groups of neurons within a multimodal escape circuit (Figure 8A) (Ohyama et al., 2015). Pruning fine terminal branches from the EM reconstructions (mimicking light level reconstructions) has little impact on cluster assignments; therefore, NBLAST clustering of coarsely skeletonized neurons could be an important step to organize EM connectome data.

We next show two examples applying NBLAST to single-cell data from another invertebrate, the monarch butterfly, and a vertebrate, the larval zebrafish (Figures 8B and 8C). Clustering 29 monarch butterfly neurons from the central complex (Heinze et al., 2013) largely matches neuronal types defined by expert neuroanatomists—the few discrepancies were reviewed with the data provider and determined to be cases where computationally defined cell groups revealed features that were orthogonal to expert classification but still a valid classification.

The zebrafish data consisted of 55 mitral cells (second-order olfactory neurons) projecting to a variety of higher brain areas (Miyasaka et al., 2014). NBLAST clustering identified clearly distinct morphological groups (Figure 8C). Very similar neurons were co-clustered both by our algorithm and that of the original authors, but clustering of distantly related neurons was distinct. Only future experiments will show if one clustering has more functional relevance.

In our final example, we apply NBLAST to a distinct but experimentally vital form of neuroanatomical image data. Circuit neuroscience in many model organisms depends on manipulating circuit components with cell-type-specific driver lines. We have registered (Manton et al., 2014) and processed image data from the most widely used *Drosophila* collection, 3,501 GMR driver lines generated at the Janelia Research Campus (Jenett et al., 2012). We applied an image processing pipeline emphasizing tubular features (Masse et al., 2012), generating a vector cloud representation identical to that used elsewhere in this paper. These data (9 Gb for 3,501 image stacks) can be queried with single neurons or tracings in less than 30 s on a desktop computer. To demonstrate this approach at scale, we mapped GAL4 data to the same template space (Manton et al., 2014) as the FlyCircuit single neurons (merging these data in silico) and computed NBLAST scores for 16,129 neurons against 3,501 driver lines. We provide a simple web server for these queries at jefferislab.org/si/nblast/on-the-fly. We showcase this by identifying GAL4 driver lines targeting the sexually dimorphic mAL neuron population (Figures 8D and 8D′). We selected ten mAL neurons and then examined the ten GAL4 lines with the highest mean scores. The top hit line R43D01 has just been identified as targeting this population (Kallman et al., 2015), and all the top ten hits target the same population.

As a second example, we looked at driver lines labeling olfactory PNs targeting the CO_2_-responsive V glomerulus. Comprehensive single-cell labeling identified classes critical for behavioral responses to different CO_2_ concentrations (Lin et al., 2013). However, one class highly selective for the V glomerulus could not be functionally studied because no GAL4 line was identified. Searching this neuron, we found the fourth hit (R86A05) was highly selective for this cell type (Figure 8E). Finally, we take an auditory interneuron (AMMC-AMMC PN1; Figures S6 and S2) and a presumptive visual interneuron of the anterior optic tubercle. The top ten hits for both neurons included numerous matching GAL4 lines; we display one example for each in Figure 8F. Although all of these lines label multiple neuronal classes, NBLAST enables very rapid identification of lines containing a neuronal population of interest that could be used for the construction of completely cell-type-specific lines by intersectional approaches (Luan et al., 2006).

## Discussion

Comprehensive mapping of neuronal types in the brain will depend on methods for unbiased classification of pools of thousands or millions of individual neurons. Comparison of neurons relies strongly on morphology and brain position, essential determinants of connectivity and function. A neuron similarity measure should (1) be accurate, generating biologically meaningful hits; (2) be computationally inexpensive; (3) enable interactive searches for data exploration; and (4) be generally applicable. NBLAST satisfies all these criteria.

First, NBLAST correctly distinguishes closely related types across a range of major neuron groups, achieving 97.6% accuracy for 35 types of olfactory PNs. Unsupervised neuron clustering based on NBLAST scores correctly organized neurons into known types. We did find that neuron sizes (especially when very small) can influence the algorithm: one future direction is to convert raw scores into an expectation (E) value that accounts for the size of a neuron and the database in direct analogy to the results of Karlin and Altschul (1990) for sequence alignments, although the problem appears more complicated for neurons.

Second, NBLAST searches are fast, with pairwise comparisons taking about 2 ms on a laptop. Furthermore, for defined datasets all-by-all scores can be pre-computed, enabling highly interactive analysis. With data volumes increasing, one effective approach to handle much larger numbers of neurons will be to compute sparse similarity matrices, storing the top *n* hits for a given neuron. Alternatively, queries could be computed only against the non-redundant set of neurons that collectively embody the structure of the brain (analogous to UniProt; Suzek et al., 2007). For the fly brain, this could not exceed 50,000 neurons (due to bilateral symmetry), and we expect the actual number to be ∼5,000. Our clustering of all 16,129 FlyCircuit neurons identified 1,052 exemplars, providing a non-redundant dataset that we use for rapid searches.

Third, NBLAST enables multiple types of analysis. Searches can use neuron fragments or tracings from complex image data as queries and databases of GAL4 lines as targets. Closely related neuronal types can be distinguished by clustering of only their terminal arbors without considering common features such as axon tracts.

Finally, one important question is the generality of our approach. This largely reduces to the relationship between length scales of neurons being examined and their absolute spatial stereotypy. Our method implicitly assumes spatial co-localization of related neurons; this is enforced by the use of image registration. Our strategy should be appropriate for any situation in which neuronal organization is highly stereotyped at the length scale of the neurons themselves. There is already strong evidence that this is true across large parts of the brain for simple vertebrate models like the larval zebrafish; indeed, we show that our NBLAST method can be applied directly to olfactory projectome data (Miyasaka et al., 2014). Mouse gene expression (Lein et al., 2007) and long-range connectivity also show global spatial stereotypy, as evidenced by recent atlas studies combining sparse labeling and image registration (Zingg et al., 2014, Susaki et al., 2014, Oh et al., 2014). Our method could be adapted for querying and hierarchical organization of these datasets by calculating an appropriate scoring matrix.

However, there are situations in which global brain registration is not appropriate. For example, the vertebrate retina has both laminar and tangential organization. Sümbül et al. (2014) recently introduced a registration strategy that showed that lamination of retinal ganglion cells is spatially stereotyped to the nearest micron. However, retinal interneurons and ganglion cells are organized in mosaics tangential to the retinal surface; global registration is not appropriate in this plane. The situation is similar for parallel columns of the outer *Drosophila* optic lobe. Possible approaches include local re-registration, mapping neurons onto a single canonical column, or amassing sufficient data so that neurons from neighboring columns/mosaics tile the brain, enabling identification of related groups by clustering or graph theoretic approaches.

Cataloguing all neuron types in the brain will rely not only on effective measures of neuronal similarity, but also on methods for automated classification of neurons into functionally relevant types. This is a challenging problem: it may be necessary to combine morphological approaches with data such as connectivity patterns, single-neuron gene expression patterns, or physiological properties to provide unambiguous automated classification (reviewed by Armañanzas and Ascoli, 2015). We have shown that NBLAST scores define a highly effective similarity metric that can be combined with hierarchical clustering and a specific dendrogram cut height to define a very wide range of neuronal classes. This approach enables very rapid exploratory analysis of new cell types even without expert neuroanatomical knowledge. Indeed, for *Drosophila* neurons it seems that NBLAST clustering is sufficient to define cell type.

Sümbül et al. (2014) recently explored the issue of defining the optimal dendrogram cut height for morphological clustering of 363 mouse retinal ganglion cells, establishing a reliable approach for these specific neurons. Nevertheless, our experience from the 16,129 neuron validation set is that differences in similarity levels within classically defined neuronal types preclude the existence of a universal value for dendrogram cut height. Some of this range (0.7 to 2 in this study) is probably due to differences in definitions: classic neuronal types may in some cases require splitting for consistency—we see evidence for this in the KC and visual PN datasets. More sophisticated statistical criteria may enable automated classification, especially when combined with measurements of, e.g., physiological or gene expression data (Armañanzas and Ascoli, 2015). However, all approaches to defining cluster numbers (i.e., statistically based cell types) depend on biological priors that must be acknowledged. Nevertheless, NBLAST’s speed and sensitivity and the size of this validation dataset represent a significant step toward fully automated classification.

Finally, we note that NBLAST can identify genetic driver lines labeling a given query neuron. The pre-computed NBLAST result matrix that we provide for the GMR GAL4 collection (Jenett et al., 2012) will be of immediate utility to *Drosophila* colleagues planning experimental studies of particular cell classes. Thus, NBLAST can provide a vital link between studies of anatomical logic and neural circuit function.

## Experimental Procedures

### Image Preprocessing

flycircuit.tw supplied 16,226 raw confocal stacks, which we converted to NRRD format with Fiji/ImageJ (http://fiji.sc/). We successfully preprocessed 16,204/16,226 total images, i.e., a 0.14% failure rate.

To make a template brain, we first averaged 17 female and 9 male brains to construct sex-specific templates using the CMTK (http://www.nitrc.org/projects/cmtk) avg_adm tool. We then averaged these two templates to generate an intersex template (FCWB) used for all subsequent analysis. We used CMTK to register images against this template first with a linear (9 degrees of freedom) and then a non-rigid registration (Rohlfing and Maurer, 2003, Jefferis et al., 2007). All registrations were checked by visual comparison with the template in Amira (academic version, Zuse Institute). Poor registrations (10%) were re-initialized using affine registration based on a Global Hough Transform (Ballard, 1981, Khoshelham, 2007) calculated with an Amira extension module available from 1000shapes GmbH, or Amira’s surface-based registration module, resulting in 16,129/16,204 successfully registered images (0.46% failure rate).

Chiang et al. (2011) included a segmented image for each neuron in their raw confocal dataset. We skeletonized this image using the Fiji plugin “Skeletonize (2D/3D)” (Doube et al., 2010) and then calculated a vector cloud representation for each skeleton (Masse et al., 2012) in R. Neurons on the right side of the brain were flipped to the left by applying a mirroring and flipping registration as described in Manton et al. (2014). We also calculated an overlap score for each neuron with the neuropil domains defined by Ito et al. (2014). See Supplemental Experimental Procedures for further details.

### Neuron Search

Our reference implementation is the nblast function in the R package nat.nblast. Fast nearest-neighbor search depends on the nabo C++ library (Elseberg et al., 2012). The scoring matrix that we used for FlyCircuit neurons was constructed by taking 150 DL2 PNs, defining a neuron type at the finest level, and calculating the joint histogram of distance and absolute dot product for the 150 × 149 combinations of neurons, resulting in 1.4 × 10^7^ measurement pairs; the number of counts in the histogram was then normalized (dividing by 1.4 × 10^7^) to give a probability density, $p_{\text{match}}$. We then carried out a similar procedure for 5,000 random pairs of neurons sampled from the FlyCircuit dataset to give $p_{\text{rand}}$. Finally, the scoring matrix was calculated as ${\text{log}}_{2}\left(p_{\text{match}}+\epsilon /p_{\text{rand}}+\epsilon \right)$, where $\varepsilon =10^{-6}$ (a pseudocount to avoid infinite values).

### Clustering

We used two methods for morphological clustering. For data subsets, we used hierarchical clustering with Ward’s method (R function hclust). Dendrograms were cut at a height selected for each class (range 0.7–2), shown by a dashed line. By default, R plots the square of the Euclidean distance as the y axis, but we use the unsquared distance.

We used affinity propagation to cluster the whole dataset (Frey and Dueck, 2007) implemented in R package apcluster (Bodenhofer et al., 2011). This iterative method finds exemplars (representative members of each cluster) and does not require a priori input on the final number of clusters. An input preference parameter (*p*) can be set to control the final number of clusters. We used p=0, since this is the value where, on average, matched segments are equally likely to have come from matching and non-matching neurons. Empirically, this produced clusters that mostly grouped neurons of the same type according to biological expert opinion.

### Computer Code and Data

There is a dedicated website at http://jefferislab.org/si/nblast. This provides online NBLAST search tools, a web version of the affinity propagation clustering, video demos, and links to all computer code and data used to generate the figures in this paper, along with the open source libraries we have written and a help forum. See Supplemental Information for details.

## Author Contributions

G.S.X.E.J. designed and implemented the NBLAST algorithm, supervised the study, and carried out initial data processing. M.C. and G.S.X.E.J. curated data, carried out validation studies of the NBLAST algorithm, and analyzed and visualized neuroanatomical data. J.D.M. refined NBLAST software, contributed to data visualization and analysis, and developed online search tools with G.S.X.E.J. A.D.O. constructed the template brain and contributed to initial image registration. S.P. implemented the Global Hough Transform registration pipeline and manual review system and investigated alternative search strategies. M.C. prepared figures with G.S.X.E.J. and J.D.M. M.C. and G.S.X.E.J. wrote the paper with J.D.M., incorporating feedback from A.D.O. and S.P.

## Supplemental Information

Document S1. Supplemental Experimental Procedures, Figures S1–S6, and Tables S1 and S2

Movie S1. Online NBLAST Search Related to Figure 2. This movie describes how to use the NBLAST web app to search for similar neurons within the FlyCircuit dataset.

Movie S2. Online NBLAST Search for GAL4 Drivers Related to Figure 2. This movie describes how to use the NBLAST web app to search for GAL4 drivers of the FlyLight collection that might label a specified neuron.

Movie S3. Online NBLAST Search for Neurons Using a Trace Related to Figure 2. This movie describes how to use the NBLAST web app to search for FlyCircuit neurons that are similar to an uploaded trace.

Document S2. Article plus Supplemental Information
