# Supplementary figures and images for: NBLAST: Rapid, Sensitive Comparison of Neuronal Structure and Construction of Neuron Family Databases
Source: Neuron. 2016 Jul 20;91(2):293–311. doi: 10.1016/j.neuron.2016.06.012 (PMC4961245; doi:10.1016/j.neuron.2016.06.012)

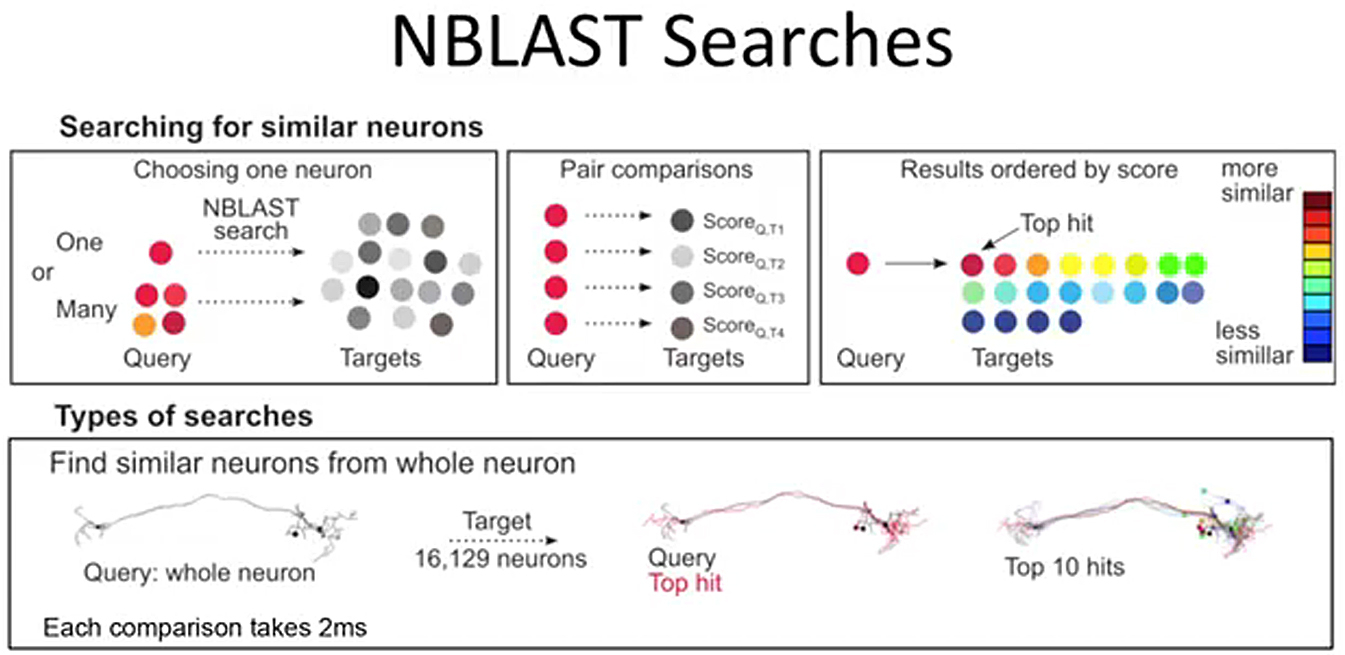

Supplement: Supplementary file 1 [file mmc6.jpg]

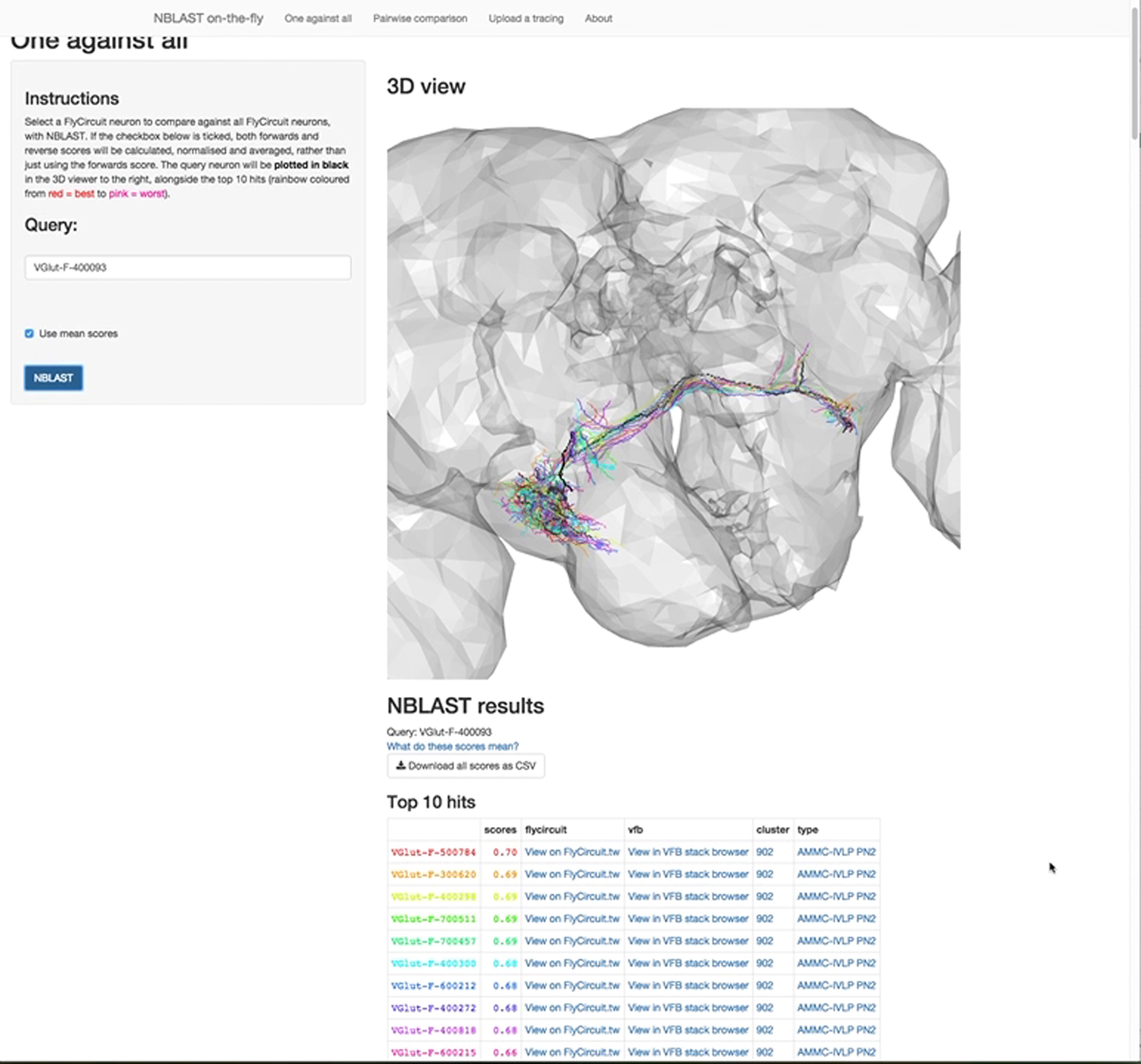

Supplement: Movie S1. Online NBLAST Search — Related to Figure 2. This movie describes how to use the NBLAST web app to search for similar neurons within the FlyCircuit dataset. [file mmc2.jpg]

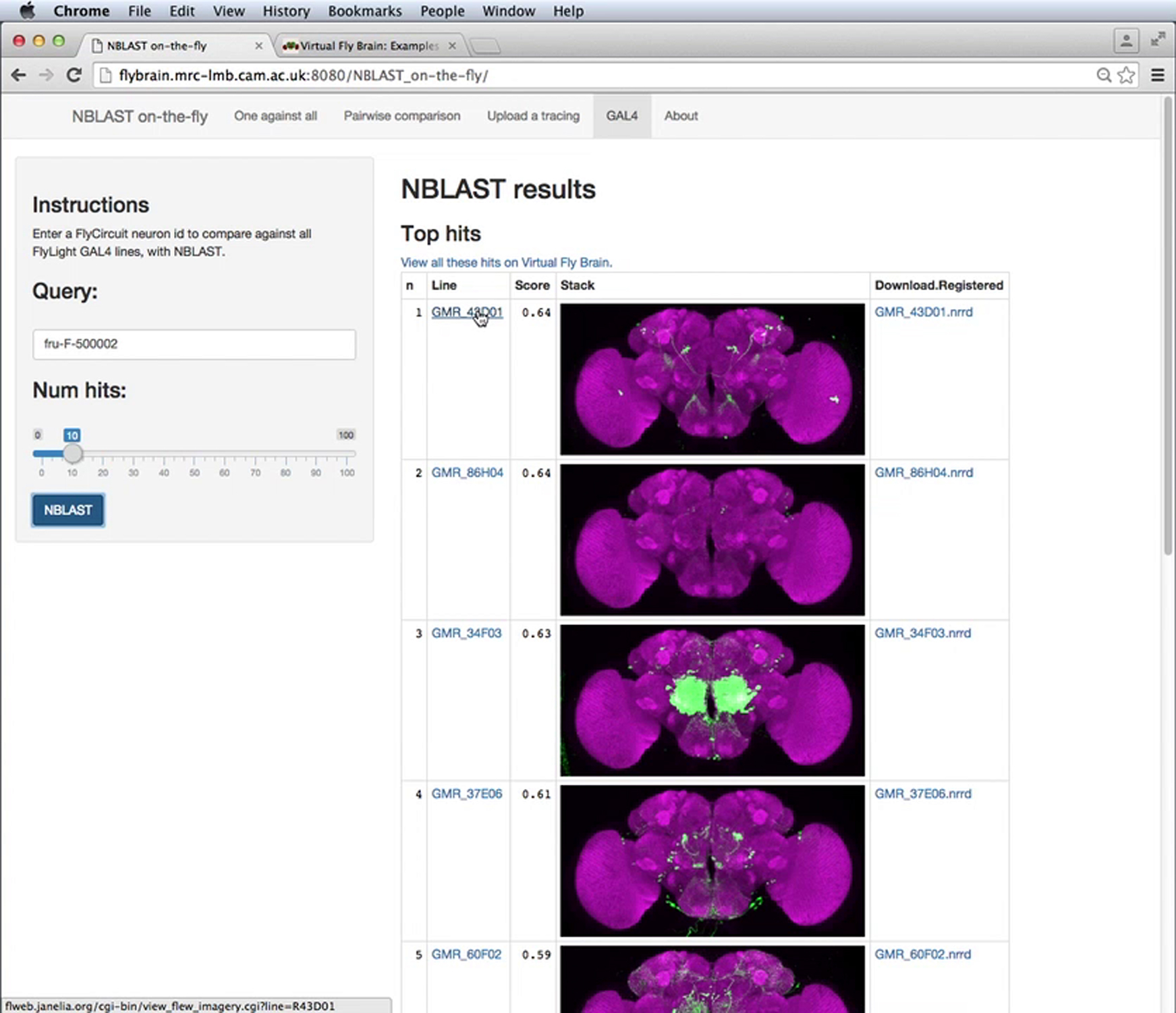

Supplement: Movie S2. Online NBLAST Search for GAL4 Drivers — Related to Figure 2. This movie describes how to use the NBLAST web app to search for GAL4 drivers of the FlyLight collection that might label a specified neuron. [file mmc3.jpg]

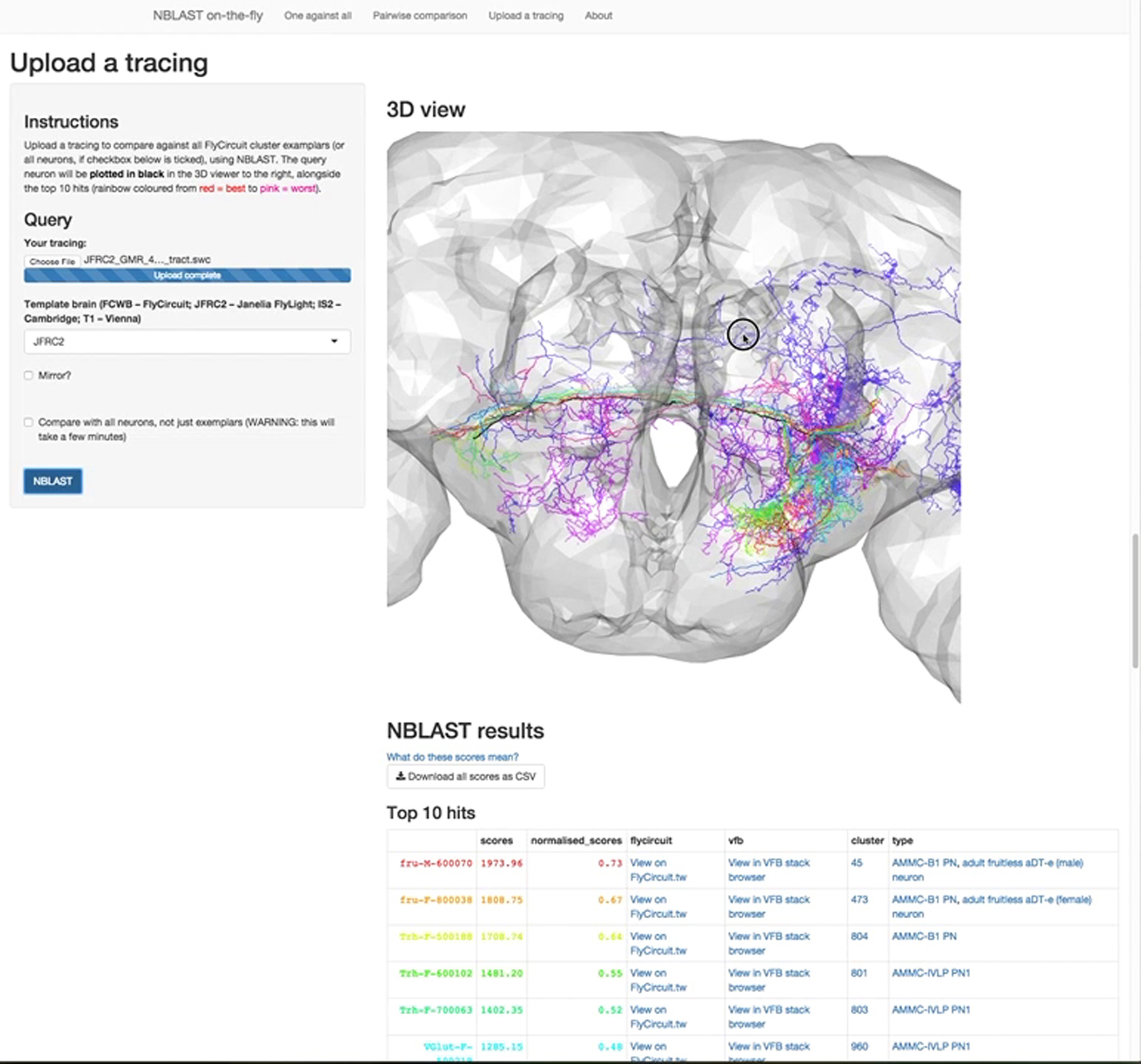

Supplement: Movie S3. Online NBLAST Search for Neurons Using a Trace — Related to Figure 2. This movie describes how to use the NBLAST web app to search for FlyCircuit neurons that are similar to an uploaded trace. [file mmc4.jpg]
