# Supplementary material for: NBLAST: Rapid, Sensitive Comparison of Neuronal Structure and Construction of Neuron Family Databases
Source: Neuron. 2016 Jul 20;91(2):293–311. doi: 10.1016/j.neuron.2016.06.012 (PMC4961245; doi:10.1016/j.neuron.2016.06.012)
Supplement: Document S1. Supplemental Experimental Procedures, Figures S1–S6, and Tables S1 and S2 [file mmc1.pdf]

Neuron, Volume *91*

Supplemental Information

NBLAST: Rapid, Sensitive Comparison of Neuronal Structure and Construction of Neuron Family Databases

Marta Costa, James D. Manton, Aaron D. Ostrovsky, Steffen Prohaska, and Gregory S.X.E. Jefferis

Supplemental Information

Supplemental Experimental Procedures

Image Preprocessing

flycircuit.tw supplied 16,226 raw confocal stacks in Zeiss LSM format on a single 2 TB hard drive in April 2011. Each stack was uncompressed, then read into Fiji/ImageJ (http://fiji.sc/Fiji) where channels were split and resaved as individual NRRD (http://teem.sourceforge.net/nrrd) files. Where calibration information was missing in the LSM metadata, we used a voxel size of (0.318427, 0.318427, 1.00935) microns as recommended by the FlyCircuit team. There were two issues to solve before images could be used for registration: 1) identifying which image channel contained the anti-Dlg (discs large 1) counterstaining 2) determining whether the brains had been imaged from anterior to posterior, or the reverse. The first issue was solved by exporting metadata associated with each LSM using the LOCI bioformats (http://loci.wisc.edu/software/bio-formats) plugin and developing heuristics to automate the identification of the channel sequence; for a minority of images metadata was missing and channel order was determined manually. Slice order could not be determined automatically from the image metadata. We therefore made maximum intensity Z projections (using the unu tool, http://teem.sourceforge.net/unrrdu) for the labeled neuron channel for each stack. Each projection was then compared with the matching thumbnail available from the flycircuit.tw website. The correlation score between the projection and thumbnail images was calculated both with and without a mirror flip across the YZ plane; a large correlation score for only one orientation was used as evidence for a given slice ordering. A small number of ambiguous results were verified manually. We successfully preprocessed 16,204/16,226 total images i.e. a 0.14 % failure rate. 12 failures were due to mismatches that could not be resolved between the segmented neuron present in the LSM file and the thumbnail image on the flycircuit.tw website; the remaining 10 failures were due to physical offsets between the brain and GFP channels or corrupt image data.

Template Brain

The template brain (FCWB) was constructed by screening for whole brains within the FlyCircuit dataset and manually selecting a pool of stacks that appeared of good quality. Separate average female and average male template brains were constructed from 17 and 9 brains, respectively using the CMTK (http://www.nitrc.org/projects/cmtk) `avg_adm` tool which takes a single brain as a seed. After five iterations the resultant average male and average female brains were placed in an affine symmetric position within their image stacks so that a simple horizontal (x-axis) flip of either template brain resulted in an almost perfect overlap of left and right hemispheres. Finally the two sex-specific template brains were then averaged (with equal weight) to make an intersex template brain using `avg_adm`. Since this template was designed to provide an optimized registration target for the FlyCircuit dataset, no correction was made for the disparity between the XY and Z voxel dimensions common to all images in the dataset. The scripts used for the construction of the template are available at https://github.com/jefferislab/MakeAverageBrain.

Image Registration

Image registration of Dlg neuropil staining used a fully automatic intensity-based (landmark free) 3D image registration implemented in the CMTK toolkit (Rohlfing and Maurer, 2003; Jefferis et al., 2007). An initial linear registration with 9 degrees of freedom (translation, rotation and scaling of each axis) was followed by a non-rigid registration that allows different brain regions to move independently, subject to a smoothness penalty (Rueckert et al., 1999). It is our experience that obtaining a satisfactory initial linear registration is crucial. All registrations were therefore manually checked by comparing the reformatted brain with the template in Amira (academic version, Zuse Institute, Berlin), using ResultViewer https://bitbucket.org/jefferis/resultviewer. This

identified about 10 % of brains with poor initial registrations. For these images a new affine registration was calculated using a Global Hough Transform (Ballard, 1981; Khoshelham, 2007) with an Amira extension module available from 1000shapes GmbH; the result of this affine transform was again manually inspected. In the minority of cases where this approach failed, a surface based alignment was calculated in Amira after manually aligning the two brains. Following satisfactory initial affine registration, a non-rigid registration was calculated. Finally each registration was checked manually in Amira against the template brain. This sequential procedure was resulted in successfully registration of 16,129/16,204 preprocessed images, giving a registration failure rate of 0.46 %.

Image Postprocessing

The confocal stack for each neuron available at `http://flycircuit.tw` includes an 8 bit image containing a single (semiautomatically) segmented neuron prepared by Chiang et al. (2011). This image was downsampled by a factor of 2 in x and y, binarized with a threshold of 1 and skeletonized using the Fiji plugin 'Skeletonize (2D/3D)' (Doube et al., 2010). Dot properties for each skeleton Masse et al. (2012), were extracted using the `dotprops` function of our new nat package for R. This converted each skeleton into segments, described by its location and tangent vector. Neurons on the right side of the brain were flipped to the left by applying a mirroring and a flipping registration as described in Manton et al. (2014). The decision to flip a neuron depended on earlier assignment of each neuron to a brain hemisphere using a combination of automated and manual approaches. Neurons whose cell bodies were more than 20 µm away from the mid-sagittal YZ plane were automatically defined as belonging to the left or right hemisphere. Neurons with cell bodies inside this 40 µm central corridor were manually assigned to a hemisphere, based on cell body position (right or left side), path taken by the primary neurite, location and length of first branching neurite. For example, neurons that had a cell body on the midline with significant innervation from the first branching neurite near the cell body on the left hemisphere, with the rest of the arborization on the right, were assigned to the left side and not flipped.

The cell body positions used were based on those published on the `http://flycircuit.tw` website for each neuron; these positions are in the space of the FlyCircuit female and male template brains (typical_brain_female and typical_brain_male). In order to transform them into the new FCWB template that we constructed, affine bridging registrations were constructed from the typical_brain_female and typical_brain_male brains to FCWB and the cell body positions were then transformed to this new space. Since these cell body positions depend on two affine registrations (one conducted by Chiang et al. (2011) to register each sample brain onto either their typical_brain_female or typical_brain_male templates and a second carried out by us to map those template brains onto our FCWB template) these positions are likely accurate only to ±5 microns in each axis.

We also calculated an overlap score for each neuron with the neuropil regions defined by Ito et al. (2014). We converted the JFRC2 space segmented neuropil volume available from virtualflybrain.org to the FCWB template space used in this study using the approach of Manton et al. (2014).

NBLAST Design

The algorithm design process was primarily motivated by a requirement for rapid and sensitive searches of neuron databases. It was necessary to consider both the data structure and the similarity algorithm jointly in the face of the design requirements. Our ideas in this area have been under independent development since 2007, but our efforts were strongly motivated by a very large single neuron dataset (Chiang et al., 2011) which offered the possibility of validating candidate algorithms at scale. We begin by summarizing published algorithms and data structures that represent different strategies that we were aware of as our own began to mature.

Mayerich et al. (2012) applied general graph similarity metrics (reviewed by Conte et al., 2004) to compare neurons represented as fully-connected graphs with a ground truth reconstruction; however this approach emphasizes the details of branching structure over positional information. Basu et al. (2011) decomposed neuronal trees into a family of unbranched paths proposing a geometric measure of similarity that could include positional informa-

tion, however the approach was not further developed or validated. Further simplifying the neuronal representation, Cardona et al. (2010) decomposed single unbranched neurites into sequences of vectors and used dynamic programming to find an optimal 3D alignment. Critically, they validated this approach on a database of a few hundred traced structures, achieving very high classification accuracy. However as noted in the main Introduction, this algorithm treats each unbranched neuronal segment as a separate alignment problem, so there is no natural way to handle trees with many such segments.

Recently the approach of Wan et al. (2015) combining graph matching with 3D positional information represents a natural combination of these different methods. However it seems restricted to a specific set of problems since 1) computation times are of the order minutes per neuron pair 2) it is restricted to fully traced neurons 3) it does not presently handle partial matches 4) it is not designed to use absolute positional information of neurons in a brain or brain structure.

One other approach developed in parallel to our own algorithm was presented by Ganglberger et al. (2014) who compared it to our earlier work (Masse et al., 2012). This uses a flexible data representation in which co-registered volumetric image data are smoothed and a vector field is calculated representing the local direction of tubular objects in the image. This approach has some advantages of the method that we will now describe but the use of large image volumes severely limits the throughput that can be achieved (the authors reported loading data for 1000 brains into memory over 15 mins). Furthermore the relatively small test dataset available to those authors did not allow them to make a strong case for the sensitivity of their algorithm.

Our first application would be data acquired in *Drosophila*, where previous studies using image registration have shown a high degree of spatial stereotypy (standard deviation of landmarks ~2.5 µm in each axis for a brain of 600 µm in its longest axis, Jefferis et al., 2007). Therefore one key design decision was to use co-registered data rather than calculating similarity using features of the neuron that are independent of absolute spatial location.

On the algorithm side, the key initial design decision was whether to develop a direct pairwise comparison algorithm or to use a form of dimensional reduction to map neuronal structure into a lower-dimensional space. The major advantage of the latter approach is that the similarity between neurons can be computed directly and almost instantaneously in the low dimensional space. However, the construction of a suitable embedding function either requires existing knowledge of neuronal similarity (likely supplied by experts in the form of large amounts of training data), huge amounts of unlabeled data that enable direct learning of features (e.g. Le et al., 2012), or a strategy based on successful extraction of key image features.

A number of considerations made us favor the approach of direct pairwise comparison. First, we suspected that it would be possible to make a more sensitive algorithm by working with the original data. Second, the amounts of image data available did not seem large enough to avoid a requirement for extensive labeled training data. Third, we reasoned that our own intuitions about neuronal similarity could be better expressed in the original physical space of the neuron than in a low dimensional embedding. Our own exploratory analysis in which we summarized each neuron in different ways as feature vectors of the same dimension and used a comparison function in the feature space (SP, GSXEJ unpublished observations) confirmed that constructing a sensitive metric of this sort is challenging.

The selection of a pairwise similarity metric meant that we had to give particularly careful consideration to performance issues in the design phase. We set two practical performance targets: 1) being able to carry out searches of a single neuron against a database of 10,000 neurons in less than a minute on a simple desktop or laptop computer. 2) Being able to complete all-against-all searches for 10,000 neurons (10^8 comparisons) in < 1 day on a powerful desktop computer. These targets meant that each elementary comparison operation should take around 5 ms or less. Image pre-processing carried out once per neuron would therefore be a good investment if it reduced the time taken for each pairwise comparison. These considerations prompted us to generate a spatially registered, compact representation of each neuron as a separate pre-processing step, rather than develop an algorithm that simultaneously solved both the spatial alignment and similarity problem.

NBLAST Scoring

As described in the main results section, we defined NBLAST raw scores as the sum of segment pair scores:

$$S(\text{query}, \text{target}) = \sum_{i=1}^{n} f(d_i, |\vec{u}_i \cdot \vec{v}_i|). \tag{3}$$

We initially experimented with a function based on expert intuition:

$$f = \sqrt{|\vec{u}_i \cdot \vec{v}_i| \exp\left(-d_i^2/2\sigma^2\right)}. \tag{4}$$

This includes a negative exponential function of distance (related to the normal distribution), with a free parameter σ based on our previous estimates of the variability in position within the fly brain of landmarks after registration (Jefferis et al., 2007; Yu et al., 2010) set to 3 µm. Although this provided a useful starting point, we were unhappy with a scoring system that required parameters to be specified rather than derived empirically from data. This then motivated us to investigate the statistical scoring approach based on log probability ratios. This single parameter approach may still be helpful in some situations where insufficient neurons are available in order to define a statistical scoring matrix (we used 150 similar neurons and 5,000 random pairs). It can also enable a bootstrapping approach for new datasets in order to help identify similar pairs of neurons that can then be used to define a full scoring matrix.

Neuron Tracing

Neuron tracing for some sample queries was carried out in Amira (commercial version, FEI Visualization Sciences Group, Merignac, France) using the hxskeletonize plugin (Evers et al., 2005) or in Vaa3D (Peng et al., 2014) on registered image data. Traces were loaded into R using the `nat` package. When necessary, they were transformed into the space of the FCWB template brain (Manton et al., 2014).

Olfactory Projection Neuron Analysis

We started by manually classifying the 400 uPNs in the FlyCircuit dataset by glomerulus, neuroblast lineage, and axon tract, using the original image stacks. The definition of the manual gold standard annotations was an iterative process that took several days. The first round accuracy was about 95 %. Numerous discrepancies were revealed by subsequent NBLAST analysis and difficult cases were resolved by discussion between two expert annotators before finalizing assignments. We excluded 3 neurons for which no conclusion could be reached. We found a very large number of DL2 uPNs, (145 DL2d and 37 DL2v), in a total of 397 neurons. Nevertheless, our final set of uPNs broadly represents the total variability of described classes and contains neurons innervating 35 out of 56 different glomeruli (Tanaka et al., 2012), examples of the three main lineage clones (adPNs, lPNs and vPNs) in addition to one bilateral uPN, and neurons that follow each of the three main tracts (medial, mediolateral and lateral antennal lobe tracts). For subsequent analysis, we removed 3 neurons for which registration failed.

We restricted the analysis in Figure S4A to types with at least two examples in the dataset and to unique pairs (if PN A and PN B were top hits for each other, we only counted them once) (n=327). There were only 8 cases in which the top hit did not match the query neuron's class. Of these, four had matches to a uPN innervating a neighboring glomerulus with identical axon projections (DL2d vs DL2v, VM5d vs VM5v) that are challenging even for experts to distinguish. There were a further four matches to multi-glomerular PNs that innervated the same glomerulus as well as some others.

There were three mis-clustered neurons in the unsupervised hierarchical clustering (Figure 4E). For one case there was substantial mis-registration of the antennal lobe overlooked by our image registration quality control; in the other 2 cases, neurons of the same type were split into two adjacent clusters.

Visual Projection Neuron Analysis

We started with the 1,052 exemplars found by affinity propagation clustering of NBLAST scores (Figure 7). We then clustered those exemplars using hierarchical clustering and found that extrinsic and intrinsic optic lobe neurons together formed a distinct "optic lobe" group within this (Figure 7C). We then collected all neurons associated with those "optic lobe" exemplars and calculated the overlap of neurons with each of the standard neuropils defined by Ito et al. (2014) (see Experimental Procedures and Manton et al., 2014 for technical details). This then allowed us to separate neurons by innervation pattern into 3 groups: 1) ipsilateral optic lobe neuropils only (see Figure S5D for intrinsic optic lobe neurons), 2) ipsilateral and central brain neuropils (unilateral VPNs, uVPNs) or 3) both optic lobes and central brain neuropils (bilateral VPNs). This selection procedure resulted in a set of 1,793 uVPNs, 72 bilateral VPNs and 2,892 intrinsic optic lobe neurons.

Hierarchical clustering of the unilateral VPNs (uVPNs) resulted in a dendrogram, which we divided into 21 groups (I-XXI), in order to isolate one or a few cell types by group based both directly on morphological stereotypy and on previous literature (Otsuna and Ito, 2006) (Figure 5A–A' and Figure S5A–A').

To isolate uVPNs that overlap the lobula, anterior optic tubercle (AOTU) and posterior ventrolateral protocerebrum (PVLP), we took neuron skeletons from groups I–III uVPN and isolated the axon arbors innervating the two latter neuropils (Figure 5B). A new clustering based on the scores of these partial skeletons, allowed us to identify 7 different groups (1-7). A clear distinction between neurons that innervated the PVLP (groups 1, 2) and those that extensively innervated the AOTU (groups 3–6) was evident.

To isolate uVPNs that overlap the lobula, PVLP- and posterior lateral protocerebrum (PLP) we performed a similar analysis with uVPN groups that had dendritic innervation restricted to the lobula and axons projecting to the PVLP and PLP (groups IV, VI, VII and XI) (Figure S5A'). Following the same strategy, we re-clustered neurons based on the scores calculated only for the axon arbors that overlapped with the PVLP or PLP (Figure S5B).

Auditory Neuron Analysis

We employed a two-step search strategy to identify the pool of auditory interneurons for analysis. First, for 5 of the auditory types, we used the FlyCircuit neuron named by Lai et al. (2012) as the seed neuron for an initial search. Candidate neurons were selected using strict anatomical criteria. A second search was then done using these candidates as query neurons and collecting all high scoring neurons (mean NBLAST score >0.5) generating our set for analysis of types.

Sexually Dimorphic Neuron Analysis

Males have around 30 neurons mAL, females only 5; they regulate wing extension by males during courtship song (Koganezawa et al., 2010). The set of mAL neurons resulted from a search with a seed neuron, fru-M-500159. We then collected 41 hits with a mean NBLAST score greater than 0.2. Cluster analysis of the male neurons used partial skeletons that only contained the axonal and dendritic arbors (Figure 6C). We used this approach because all mAL neurons have a very similar primary neurite and main axon tract; differences in the position of these parts of the neuron are unlikely to be of great functional significance. In contrast differences in dendritic arborisations likely alter input connectivity including pheromone information detected by chemoreceptors on the male forelegs (Koganezawa et al., 2010; Kohl et al., 2015). The 3 different male types differed in the length of the ipsilateral ventral projection; this feature has previously been proposed as the basis of a qualitative classification of mAL neurons (Kimura et al., 2005).

There are around 20 P1 neurons that develop from the pMP-e *fruitless* neuroblast clone. They have extensive bilateral arborizations in the PVLP and ring neuropil, partially overlapping with the male-specific enlarged brain regions (MER) (Kimura et al., 2008; Cachero et al., 2010). The set of P1 neurons for analysis was identified by searching the FlyCircuit dataset with a tracing of the distinctive primary neurite of a pMP-e clone (Cachero et al.,

2010). Hierarchical clustering of the top hits, after manual verification, identified a subset consisting solely of P1 neurons which was used in the subsequent analysis.

NBLAST of Other Species

The 29 monarch butterfly neurons were downloaded from `http://insectbraindb.org` and scaled by a factor of 5 before clustering so that their length scale matched the adult *Drosophila* brains that we used. The 55 zebrafish mitral cells were provided by Dr Y. Yoshihara and S. Seung and colleagues. A bridging registration was constructed using CMTK to map opposite brain hemispheres onto each other. All neurons were then mapped onto the right-hand side of the larval fish brain before clustering.

Online resources

The image processing pipeline used two custom packages for the R statistical environment (`http://www.r-project.org`): `https://github.com/jefferis/nat` and `https://github.com/jefferis/nat.as` that coordinated processing by the low level registration (CMTK) and image processing (Fiji, unu) software mentioned above. NBLAST neuron search is implemented in R package **nat.nblast** available at `https://github.com/jefferislab/nat.nblast`. Analysis code specific to the flycircuit dataset is available in a dedicated R package `https://github.com/jefferis/flycircuit`, with a package vignette showcasing the main tools that we have developed. Further details of these supplemental software and the associated data are presented at `http://jefferislab.org/si/nblast`. The registered image dataset can be viewed in the stack viewer of the `http://virtualflybrain.org` website and all 16,129 registered single neuron images and corresponding skeletonized neurons will be available at `http://jefferislab.org/si/nblast` or on request to GSXEJ on a hard drive; the unregistered data remain available at `http://flycircuit.tw`.

Precomputed score matrices of all by all FlyCircuit neurons and all FlyCircuit neurons against all GMR GAL4 lines are distributed through the `https://github.com/jefferis/flynblastscores` R package or available via a remote web service that can return the top n hits for any neuron (see `vfb_nblast` function in package `https://github.com/jefferis/vfbr`).
The online resources accompanying this paper are listed at http://jefferislab.org/si/nblast and include the following:

- Code and instructions to generate some of the figure panels used in the paper. Instructions can be found at https://github.com/jefferislab/NBLAST_figures. A video demo is also available at http://youtu.be/LJgZejabqqg.
- The affinity propagation clustering of the flycircuit.tw dataset (as in Figure 7D), excluding intrinsic optic lobe neurons. This can be viewed online at http://jefferislab.org/si/nblast/clusters, including interactive 3D rendering of clusters powered by WebGL.
- The clusters identified by affinity propagation clustering (including intrinsic optic lobe neurons) are all indexed by the Virtual Fly Brain (http://www.virtualflybrain.org, VFB) website, which links to 3D WebGL renderings of each cluster hosted at jefferislab.org. Clusters can be identified by VFB queries for the neuropil region that they innervate. For example, search from the VFB homepage for "AMMC". From the results page, choose the query "Images of neurons with: some part here (clustered by shape)". A list of clusters, with thumbnail images is displayed; single exemplars are also displayed for each cluster, hyperlinked to the original data at flycircuit.tw. The images of the individual neurons that are part of this cluster can also be displayed in the stack browser from this page ("List individual members" or "Open all in viewer"). Clicking on a cluster thumbnail links to a page which includes a snapshot and 3D rendering of the cluster, and information about the neurons that are part of this cluster, including links to the appropriate Neuron ID pages at flycircuit.tw; a second table provides links to result pages for the most similar clusters. A video demo is available at http://youtu.be/YFsxjkdr5y8.

- An online web-app allowing on-the-fly NBLAST queries of FlyCircuit neurons against other FlyCircuit neurons, as well as queries of user-uploaded neurons against the FlyCircuit dataset, available at http://jefferislab.org/si/nblast/on-the-fly/.
- Video demos for the online resources are available at http://jefferislab.org/si/nblast/www/demos.

Supplemental Figures and Legends

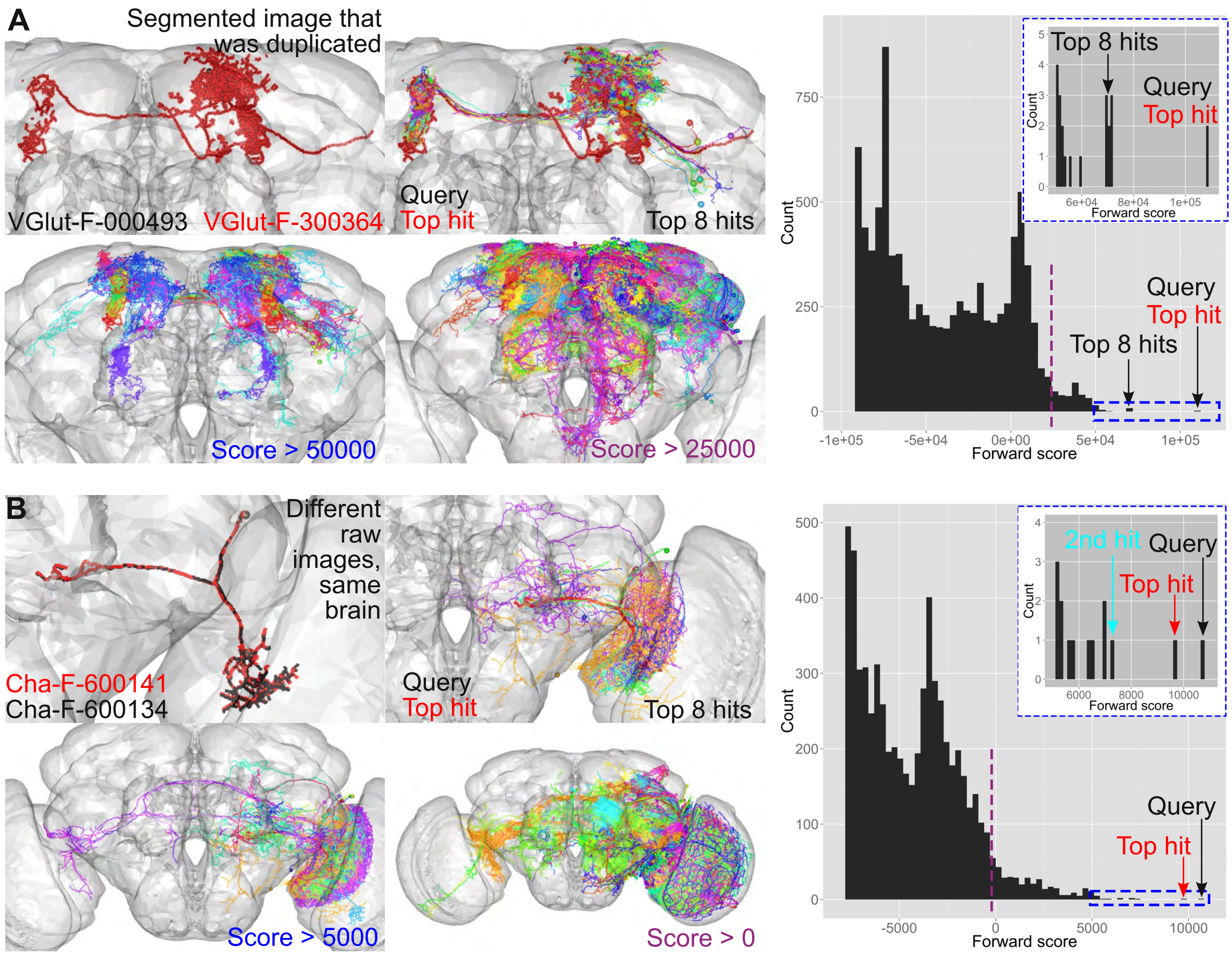

Figure S1: **Neuron search with NBLAST, related to Figure 3**

(A) NBLAST search with VGlut-F-000493 as query. Neuron plots of (from higher to lower score): the query (black) and top hit (red), top 8 hits, hits with a score over 50,000 and hits with a score over 25,000. The top hit corresponds to a segmented image that was duplicated. It perfectly overlays the query neuron. As the score decreases, so does the similarity of the hits to the query. On the right, histogram of forward scores. Only hits with scores over −100,000 are shown. The score of the query, top hit and top 8 hits are indicated. A dashed purple line marks 25,000. The left inset shows a zoomed view of the top hits (score > 50,000) (dashed blue rectangle in main plot). The score of the query, top hit and top 8 hits are indicated. **(B)** NBLAST search with Cha-F-600134 as query (black). Neuron plots of (from higher to lower score): the query and top hit, top 8 hits, hits with a score over 5,000 and hits with a score over 0. The top hit corresponds to an image of a neuron from the same brain but from a different raw image. It is very similar to the query neuron. As the score decreases, so does the similarity of the hits to the query. On the right, histogram of forward scores. Only hits with scores over −8,000 are shown. The score of the query and top hit are indicated. A dashed purple line marks 0. The left inset shows a zoomed view of the top hits (score > 5,000) (dashed blue rectangle in main plot). The score of the query, top hit and second top hits are indicated.

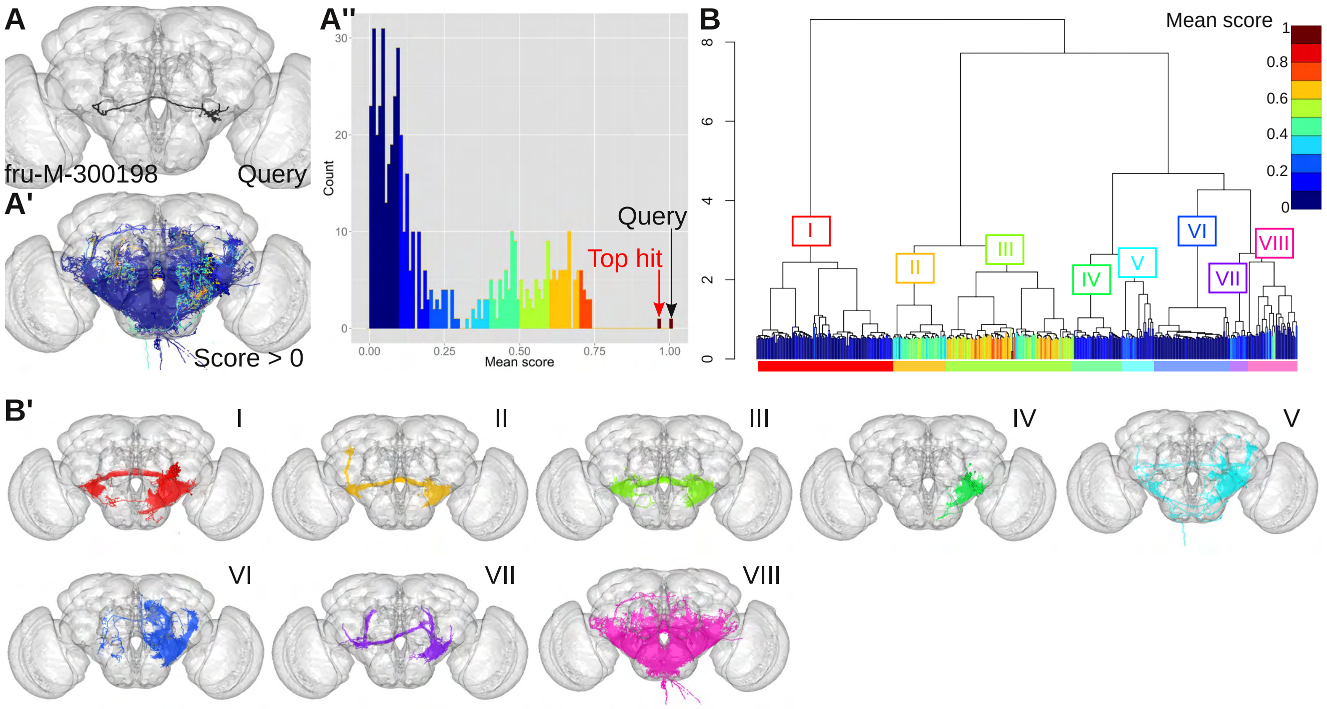

Figure S2: **NBLAST search using mean scores, related to Figure 3**
(A) The query neuron fru-M-300198 (same as in Figure 3B). **(A')** Neuron plot of the hits with a mean score over 0 for a search against the query. Hits are colored by score bin (10), as in A". **(A")** Histogram of mean scores for hits against fru-M-300198 with a score over 0 divided into 10 bins (indicated in the scale bar in B). **(B)** Hierarchical clustering of hits with a mean score over 0. The leaves of the dendrogram are colored by score (same as in A"), and as shown in the scale bar. The dendrogram was divided into eight groups (I–VIII), with each one being assigned a color, shown on the colored rectangle below the leaves. The query neuron is in group III, and the hits with the higher scores are in groups II and III. **(B')** Neuron plots corresponding to the dendrogram groups (I–VIII), following the colors assigned to each group. Groups II and III, corresponding to the highest scores, are the most similar neurons to the query.

Supplemental Kenyon cell analysis

Dataset collection and dividing into types We collected the dataset of initial KCs by performing a forward and reverse search against all neurons using one identified KC (fru-M-500225). We then selected neurons that had both raw scores above −2,500 (2,088 neurons). We performed affinity propagation clustering (Frey and Dueck, 2007) of these neurons, obtaining 59 clusters, and manually verified each one, resulting in 1,562 neurons being identified as KCs. An additional search for high scorers against these KC exemplars uncovered an extra 102 neurons, bringing the total number of KCs used in our analysis to 1,664, representing 10.3 % of the FlyCircuit dataset.

We performed hierarchical clustering of the KCs, based on the NBLAST scores, and divided the dendrogram into two groups (Figure 4A and Figure S3A). Contrary to expectations, one group contained both the γ and α′/β′ neurons, whereas the other group consisted exclusively of α/β neurons (Figure S3B–D), the largest subset in our sample. We separated α′/β′ from the γ neurons in a subsequent hierarchical clustering of this group. We performed additional analysis for each of the neuron types.

Analysis of γ neurons Hierarchical clustering of the 470 γ neurons resulted in a dendrogram which we divided into three groups (I–III) (Figure 4B'). The number of clusters was chosen by visual inspection in order to reveal differences in morphology and organization between the groups. Groups I and III corresponded to the classical γ neurons while group II matched atypical γ neurons. There were differences in neurite positioning in the calyx, from medial to lateral, with group I being the most medial, followed by groups II and III. There were also differences in the gamma lobe, with group II occupying the anterodorsal region, while groups I and III were mostly mixed in the rest of the lobe. A subsequent clustering analysis of the classical γ neurons divided into 4 groups (groups A-D) revealed that there were differences between the groups in their medial to lateral position in the calyx. These differences correlated to a certain degree with differences in the dorsal/ventral position of the projections in the γ lobe, with groups C and D, the most medial, being also the most dorsal (Figure 4B'). These observations suggest that the relative position of the projections of classical γ neurons is maintained at the calyx and γ lobe.

In order to understand if the relative position of the classical γ neurites was maintained in between the calyx and the γ lobe, we clustered the neurons based on the scores of the segments in the peduncle. We took neuron skeletons from classical γ neurons and isolated the axon arbors that co-localized with the peduncle volume (Figure S3E'). We then carried out a new clustering based on all-by-all NBLAST scores of these partial skeletons, cutting the dendrogram at a level defined by visual inspection (4 groups). The overall organization almost fully recapitulated the positioning of the neurites in the whole neuron analysis (compare Figure 4B' with Figure S3E'). A clear and expected lamination was found in the peduncle, with neurites occupying the most outer stratum. Differences in the medial to lateral positioning of neurites in the calyx followed the previously observed organization, with the most medial groups occupying the dorsal region of the gamma lobe. The overall organization almost fully recapitulated the positioning of the neurites in the whole neuron analysis. Thus, the stereotypical organization of the classical γ neurons is maintained throughout the neuropil.

Group II of the γ neurons matched atypical γ neurons (γ dorsal neurons) (Aso et al., 2014) with neurons that extended neurites posteriolaterally in the calyx and projected to the most dorsal region of the γ lobe (Figure 4B"). Hierarchical clustering of these neurons resulted in a dendrogram that we divided into 3 groups (a–c). This number of groups isolated the previously identified subtype of atypical γ neurons – γd neurons (Aso et al., 2009) – into one group (group a). These neurons extend neurites ventrolaterally at the level of the calyx (identified as ventral accessory calyx) (Aso et al., 2014). The other 2 groups (b, c) correspond to uncharacterized types. Although they project to a similar region in the γ lobe, their dendrites do not extend laterally and their calyx neurites are longer than γd neurons.

Analysis of α′/β′ neurons Hierarchical clustering analysis of the α′/β′ neurons and separation into 4 groups highlighted the characterized subtypes of α′/β′ neurons (Figure S3C). They differ in their anterior/posterior position in the peduncle and β′ lobe with three types described - α′/β′ anterior and posterior (α′/β′ap) and α′/β′ medial (α′/β′m)

(Tanaka et al., 2008, Y. Aso, personal communication)). Although we were unable to unambiguously assign a α′/β′ subtype to each group i–iv because our sample was too small, there were clear trends. Neurites of neurons in groups i and iv were more anterior than the other 2 groups (ii, iii) in both the peduncle and β′ lobe. These relative positions were not maintained in the calyx, with the two the anterior groups (i, iv) occupying either a medial or a lateral position.

Analysis of α/β neurons The largest subset of KCs corresponds to α/β neurons (Figure 4D). During the analysis of this group we found 18 neurons that did not correspond to α/β cells, since they innervated either only the β or the α lobes, and they were removed from the analysis. We performed hierarchical clustering on the remaining 1,091 cells and divided the resulting dendrogram into four groups (1–4) (Figure 4D'), which matched the four neuroblast lineages from which they originate (Zhu et al., 2003). The relative position of the neurites of the four groups within the calyx is somewhat maintained in the peduncle, with the lateral neuroblast clones (group 1 AL; group 2 PL) extending along the dorsolateral peduncle, while the medial clones (group 3 AM; group 4 PM) occupy a more ventromedial region. Hierarchical clustering of each group revealed an expected common organization for all neuroblast clones (Figure 4D" and Figure S3D'). For all groups there was a clear distinction between the late born core (α/β core, α/β-c) and early born peripheral neurons (α/β surface, α/β-s). Core neurons are on the inside stratum of the α lobe. They are also reported to occupy the inside stratum of the peduncle and β lobe (Tanaka et al., 2008). We were unable to observe this, although the projections of α/β core neurons were ventral to α/β surface neurons in both the peduncle and β lobe. There was also a trend for α/β surface neurons to occupy a more medial position in the calyx in comparison to α/β core ones. A subgroup of group 2 corresponded to the α/β posterior or pioneers neurons (α/βp). The α/βp neurons are the earliest born α/β and they innervate the accessory calyx, run along the surface of the posterior peduncle into the β lobe but stop before reaching the medial tip (Tanaka et al., 2008). A new clustering based on peduncle position of the neuron segments did not recapitulate the relative positions of the calyx neurites for each of the neuroblast clones observed in the whole neuron analysis suggesting that the relative position of the α/β neurons in the peduncle does not completely reflect their stereotypical organization in the calyx.

In order to investigate the stereotypical organization of α/β neurites, we performed a similar analysis as for the classic γ neurons, isolating the axon arbors that co-localized with the peduncle for groups 1 to 4 (Figure S3E"). The new clustering based on peduncle position of these partial neuron skeletons did not recapitulate the relative positions of the calyx neurites for each of the neuroblast clones observed in the whole neuron analysis (compare Figure 4D' with Figure S3E"). In addition, there was no clear organization of neurites in the α lobe that correlated with their position in the peduncle.

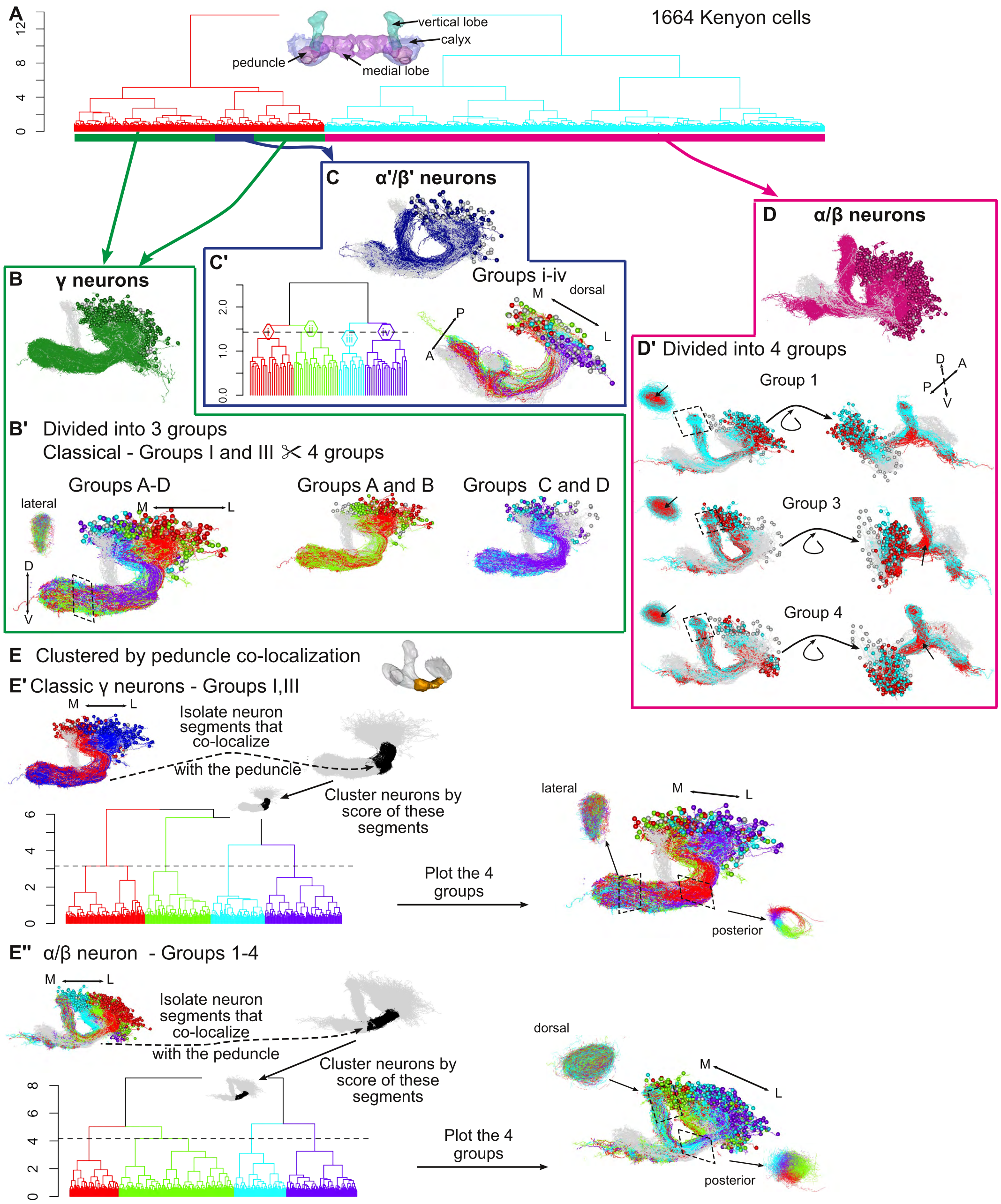

Figure S3: **NBLAST search and classification of hits reveals Kenyon cells subtypes, related to Figure 4**

(A) Hierarchical clustering (HC) of Kenyon cells (n=1664). Bars below the dendrogram indicate the γ (green), α′/β′ (blue) and α/β neurons (magenta), h=8.9. **(B)** Neuron plot of γ neurons. **(B')** HC of the classic γ neurons, corresponding to groups I and III in Figure 4B', divided into four groups (A–D). Neuron plots of groups A–D, A–B and C–D. **(C)** Neuron plot of α′/β′ neurons. **(C')** HC of α′/β′ neurons, divided into four groups (i–iv), h=1.43. **(D)** Neuron plot of α/β neurons. **(D')** α/β neurons divided into four groups (see Figure 4D') and neuron plots of groups 1, 3 and 4. Each group was divided divided into 2 subgroups, separating the neurons into surface (cyan) and core (red) (same as in Figure 4D"). Peripheral neurons occupied a more lateral calyx position and were dorsal to core neurons in the peduncle and β lobe. Lateral oblique, posterior oblique and a dorsal view of a peduncle slice (position indicated by dashed rectangle) are shown. **(E)** Reclustering of Kenyon cells based on the co-localization of neurons segments in the peduncle. The neuron segments that co-localize with the peduncle were isolated, followed by HC of the neurons based on the NBLAST score of the segments. **(E')** HC of the neurons segments of classic γ neurons, groups I and III (see B') divided into 4 groups, h=3.16. Neuron plot of the 4 groups. A posterior view of a slice of the peduncle shows an expected clear organization. It correlates to the position of the neurons in the calyx, with more medial neurons (cyan and green) being dorsal and ventral in the peduncle than more lateral neurons (red and purple). No clear structural organization is discernible in a lateral view of a slice of the γ lobe. **(E")** HC of the neurons segments of classic α/β neurons, groups 1 to 4 (see Figure 4D') divided into 4 groups, h=4.16. Neuron plot of the 4 groups. A posterior view of a peduncle slice shows an expected clear organization. It correlates to the position of the neurons in the calyx, with more medial neurons (cyan and green) being ventrolateral in the peduncle than more lateral neurons (red and purple). No structural organization is discernible in a dorsal view of a slice of the α lobe. For all neuron plots, the neurons in grey correspond to the Kenyon cell exemplars.

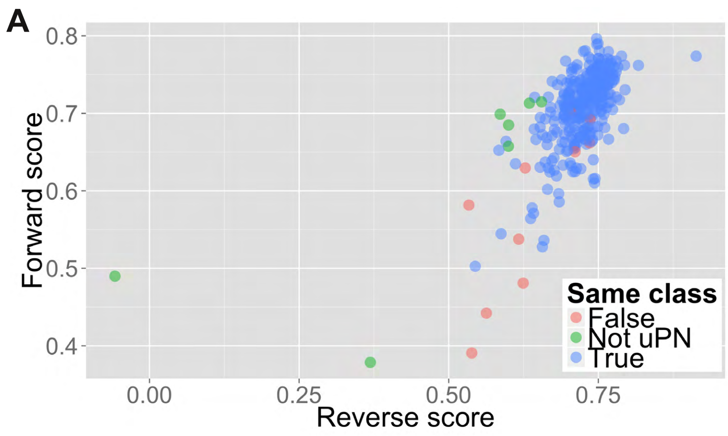

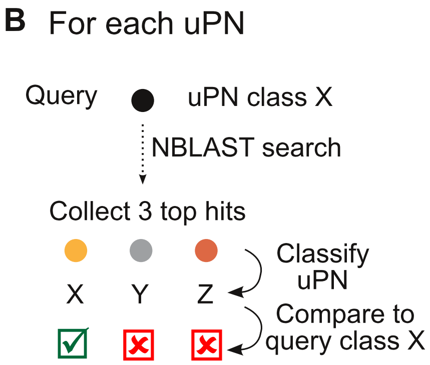

Matches to query class	%	n
Top hit	98.3	184
Any one	98.9	185
Any two	96.2	180
All three	95.2	178
		187

Figure S4: **NBLAST search and classification of hits reveals olfactory projection neuron neuronal types, related to Figure 4**
(A) Plot of the reverse and forward normalized scores for the top hit in an NBLAST search using the uniglomerular olfactory projection neurons (uPNs) as queries (n=327) against all other neurons. For each query neuron, we identified cases for which both the top hit and query were of the same class (True) (n=319); the top hit is a uPN but does not match the class of the query (False) (n=4), or the top hit is not a uPN (Not uPN) (n=4). **(B)** Percentage of neuron type matches in the top hit and top 3 hits for each uPN. The top three hits for each uPN (mean score) were collected and the neuron type of each hit and query was compared.

Dendrogram group	Neuron type	Comments
1	LC6	Lobula innervation, dorsal cell bodies, axons follow the anterior optic tract to the AOTU, turning ventrally midway to innervate the lateral PVLP.
2	LC9	As group 1, but terminating in the medial PVLP rather than extending laterally.
3	LC10B (possibly different subtype to 4)	Dorsal AOTU innervation, ventral to group 4.
4	LC10B (possibly different subtype to 3)	Dorsal AOTU innervation, dorsal to group 3.
5	New LC10 subtype	The most ventral AOTU innervation. Similar to group 7, but ventral to it in the AOTU.
6	LC10A	Axons project through ventral AOTU, turn sharply dorsally to terminate in the dorsal AOTU.
7	New LC10 subtype	Ventral AOTU innervation, dorsal to group 5.
A	LC12 subtype	Innervation in most lateral and anterior PVLP glomerulus. Note: corresponds to LC17 in Panser et al. (2015).
B	LC12 subtype	Innervation in more posterior and medial PVLP glomerulus than group A. Note: corresponds to LC12 in Panser et al. (2015).
C	LC4	Innervates a more medial PVLP glomerulus than LC12.
D	LT12	Tentative match. Class was identified in Otsuna and Ito (2006) based on a single neuron.
E	New LC class	Innervates more dorsal PVLP glomerulus than LC12. Extends along the posterior PVLP, with a sharp anterior turn. Terminates with a blunt-stick like ending in the lateral PVLP. Corresponds to LC18 in Panser et al. (2015).
F	New LT class	Similar to LT12, but with projections posterior to it, terminating in the lateral region of the superior posterior slope.
G	Unmatched	Do not correspond to a single type.

Table S1: Correspondences between hierarchical clustering groups of AOTU- and PVLP-innervating (groups 1-7) and PVLP- and PLP-innervating (groups A-G) uVPNs via NBLAST scores and previously determined neuron types (Otsuna and Ito, 2006). Related to Figure 5 and Figure S5.

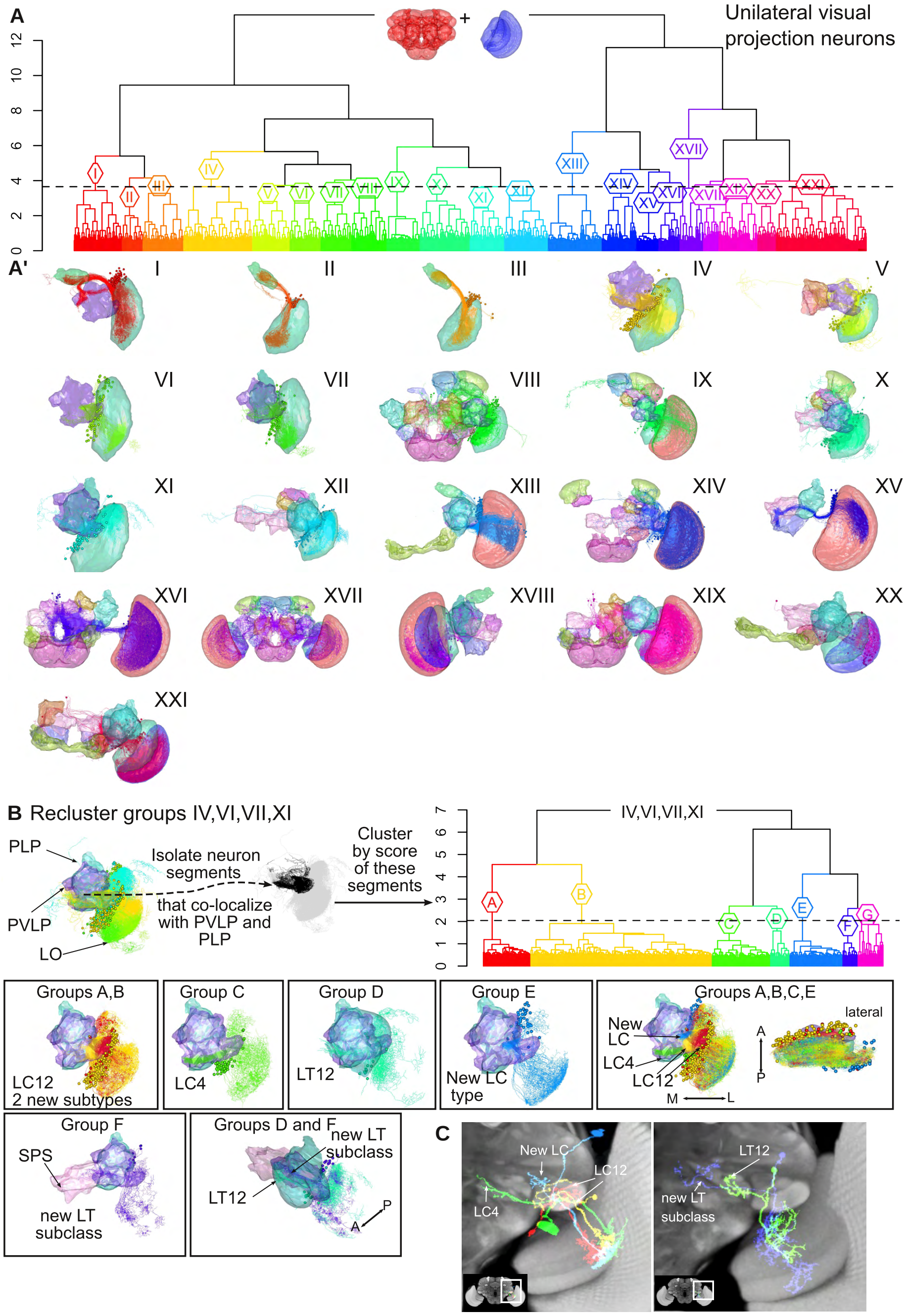

Figure S5: **NBLAST search and classification hits uncovers unilateral visual projection neurons neuronal types, related to Figure 5**
(A) Hierarchical clustering (HC) analysis of unilateral visual projection neurons (uVPNs). The dendrogram was divided into 21 groups (I–XXI), h=3.65. Inset on the dendrogram shows the neuropils considered for the overlap. **(A')** Neuron plots of groups I to XXI. The neuropils that contain the most overlap are shown. **(B)** Reclustering of uVPN groups IV, VI, VII and XI from A. The neuron segments that co-localize with either the PVLP or PLP were isolated, followed by HC of the neurons based on the NBLAST score of these neuron segments. The dendrogram was divided into seven groups (A–G), h=2.04. Neuron plots matching the dendrogram groups to known uVPN types. An anterior, a lateral or lateral oblique views are shown. Groups A and B possibly correspond to two LC12 subtypes. Group B innervates a more anterior and medial glomeruli than group A (see also C). Group C corresponds to LC4. Group D corresponds to LT12 neurons. Group E corresponds to a new LC type. Group F corresponds to a possibly new LT subclass. This analysis identified 5 distinct types, including a new subtype. **(C)** Overlay of Z projections of registered image stacks of example neurons from the types identified in B on a partial Z projection of the template brain (a different one for each panel). The white rectangle on the inset shows the location of the zoomed in area. LC: lobula columnar neuron; LT: lobula tangential neuron. **(D)** Hierarchical clustering of intrinsic optic lobe neurons. This neuron set was defined as any neuron that overlapped only one of the optic lobes and with no arborization in the central brain neuropils. Dendrogram of the intrinsic optic lobe neurons, divided into into 20 groups (I–XX) with the corresponding heatmap calculated from the neuropil overlap in the different neuropils: medulla (ME), lobula (LO), lobula plate (LOP) and accessory medulla (AME). Neuron plots corresponding to the dendrogram groups are shown below. The neuropils for which the overlap is more significant are plotted. Although some organizational structure is seen, the dendrogram groups to do not represent unique types.

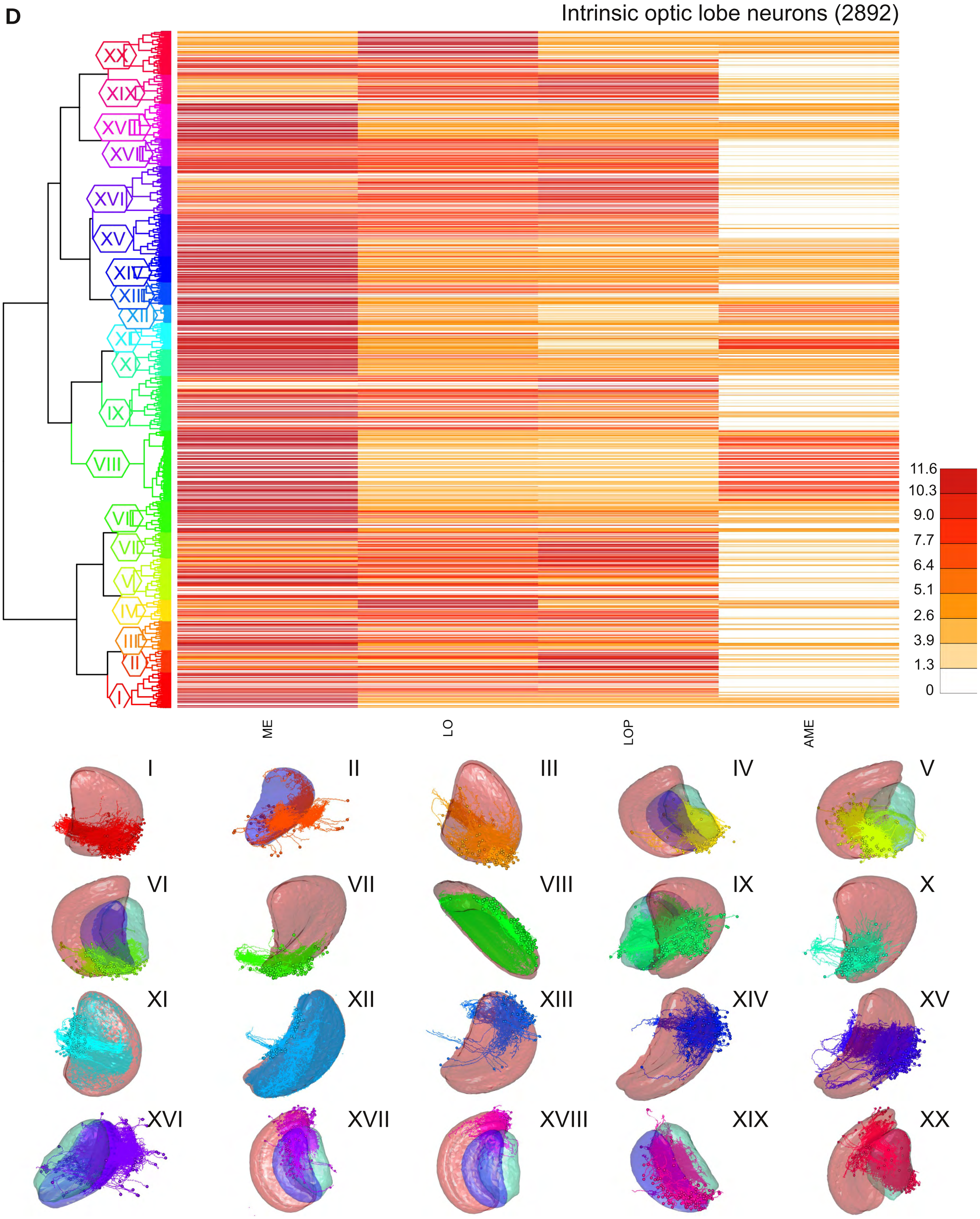
D
Intrinsic optic lobe neurons (2892)
XX
XIX
XVIII
XVII
XVI
XV
XIV
XIII
XII
XI
X
IX
VIII
VII
VI
V
IV
III
II
I
11.6
10.3
9.0
7.7
6.4
5.1
2.6
3.9
1.3
0
ME
LO
LOP
AME
I
II
III
IV
V
VI
VII
VIII
IX
X
XI
XII
XIII
XIV
XV
XVI
XVII
XVIII
XIX
XX

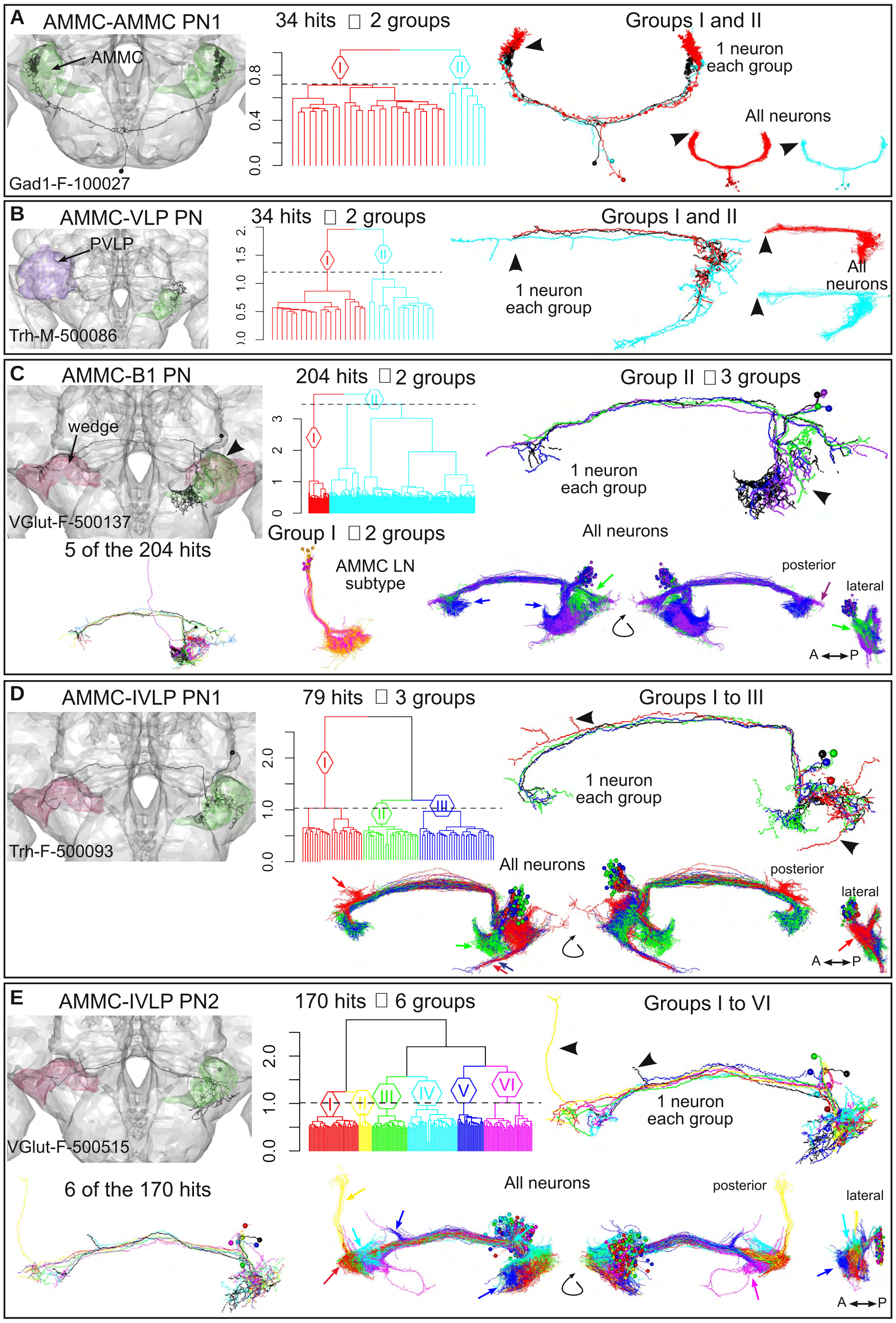

Figure S6: **NBLAST search and clustering identifies auditory neuron types, related to Figure 5**

On the left, a neuron of the types identified by Lai et al. (2012) (A-E) which was used as query. The dendrogram and neuron plots for each type (anterior, posterior and lateral views) showing either one or all neurons from each clustering group are shown on the middle and right panels. Main differences in arborization between neurons are indicated by arrowheads and arrows. **(A)** Hierarchical clustering (HC) of AMMC-AMMC projection neuron 1 (PN1). The 34 top scorers were clustered and divided into 2 groups, h=0.72. Neuron plot of the query neuron (black), and one neuron from each group (red and cyan). To the right, neuron plots of of each group. **(B)** HC of AMMC-VLP PNs. The 34 top scorers were clustered and divided into 2 groups, h=1.2. Neuron plot of the query neuron (black), and one neuron from each group (red and cyan). On the left, neuron plots of each group. **(C)** HC of AMMC-B1 PNs. Five hits of the 204 top scorers are shown on the left. The 204 top scorers were clustered and divided into 2 groups, h=3.46. Group I was matched to an unidentified type of AMMC local neurons (LN). It was clustered and divided into 2 groups, h=0.77. Group II corresponds to a mix of AMMC-B1 PNs and AMMC-IVLP PN1. After selecting the AMMC-B1 PNs, the neurons were clustered and divided into 3 groups, h=1.5. Neuron plot of the query neuron (black), and one neuron from each group (purple and green). Below, neuron plots of the 3 groups. **(D)** HC of AMMC-IVLP PN1. The 79 top scorers were clustered and divided into 3 groups, h=1.03. Neuron plot of the query neuron (black), and one neuron from each group (red, green and blue). Below, anterior, posterior and lateral view neuron plots of the 3 groups. **(E)** HC of AMMC-IVLP PN2. Six hits of the 170 top scorers are shown on the left. The 170 top scorers were clustered and divided into 6 groups, h=1.02. Neuron plot of the query neuron (black), and one neuron from each group (red, yellow, green, cyan, blue, magenta). Below, anterior, posterior and lateral view neuron plots of the 6 groups. Arrows and arrowheads indicate differences between groups. wedge in magenta, AMMC in green or PVLP in purple.

Panel	Neuron type	Comments
A	AMMC-AMMC PN1 (2 possible subtypes)	These neurons innervate both AMMCs, with a ventral cell body. Group I extends more dorsally than group II.
B	AMMC-VLP (2 possible subtypes)	These neurons innervate the ipsilateral AMMC and the contralateral VLP. Group II extends more laterally than group I.
C	AMMC-B1 PN (3 possible subtypes)	These neurons innervate the ipsilateral AMMC and IVLP and the contralateral IVLP. Blue group innervates more medial regions and has more extensive innervation contralaterally; purple group innervates more anterior and posterior regions ipsilaterally, and the green group innervates the dorsal regions in the ipsilateral AMMC.
C	New AMMC-LN type (2 subtypes)	A type of AMMC LN, with a dorsal cell body. Two possible subtypes: the magenta group innervates more dorsal regions than the orange group.
D	AMMC-IVLP PN1 (3 possible subtypes)	These neurons innervate the ipsilateral AMMC and the contralateral IVLP. Red group has a more dorsomedial ipsilateral innervation more medial regions, with some dorsal medial branches in the contralateral hemisphere; some neurons extend a long neurite ventrally ipsilaterally. The green group innervates the more ventromedial regions ipsilaterally. The blue group is similar to the green one, although it does not extend as ventrally in the ipsilateral side, and a few neurons extend a long neurite ventrally (similar to red group).
E	AMMC-IVLP PN2 (6 possible subtypes)	These neurons innervate the ipsilateral AMMC and the contralateral IVLP, with a posterior cell body. Group I innervates the more lateral regions in both hemispheres. Group II has a long dorsal branch in the contralateral hemisphere, at the lateral edge of the neuron. Group III and IV are very similar, with the latter innervating more dorsal regions in both hemispheres. Group V corresponds to the strict definition of the neuron type by Lai et al. (2012), showing a short dorsal branch just medial to the contralateral IVLP. Group VI are similar to group V, with a few neurons showing a short dorsal branch, and innervating a more ventral region in the contralateral IVLP.

Table S2: Correspondence between hierarchical clustering of auditory neuron via NBLAST scores and previously determined neuron types (Lai et al., 2012). Related to Figure 5 and Figure S6.

Supplemental Movies

Movie S1: Online NBLAST search, related to Figure 2.

This movie describes how to use the NBLAST web app to search for similar neurons, within the FlyCircuit dataset.

Movie S2: Online NBLAST search for GAL4 drivers, related to Figure 2.

This movie describes how to use the NBLAST web app to search for GAL4 drivers of the FlyLight collection that might label a specified neuron.

Movie S3: Online NBLAST search for neurons using a trace, related to Figure 2.

This movie describes how to use the NBLAST web app to search for FlyCircuit neurons that are similar to an uploaded trace.

Supplemental References

Aso, Y., Grübel, K., Busch, S., Friedrich, A.B., Siwanowicz, I., and Tanimoto, H. (2009). The mushroom body of adult Drosophila characterized by GAL4 drivers. Journal of neurogenetics *23*, 156–172.

Aso, Y., Hattori, D., Yu, Y., Johnston, R.M., Iyer, N.A., Ngo, T.T., Dionne, H., Abbott, L., Axel, R., Tanimoto, H., et al. (2014). The neuronal architecture of the mushroom body provides a logic for associative learning. eLife *3*, e04577.

Ballard, D.H. (1981). Generalizing the Hough transform to detect arbitrary shapes. Pattern Recognition *13*, 111–122.

Basu, S., Condron, B., and Acton, S.T. (2011). Path2Path: hierarchical Path-Based analysis for neuron matching. In Biomedical Imaging: From Nano to Macro, 2011 IEEE International Symposium on (IEEE), pp. 996–999.

Cachero, S., Ostrovsky, A.D., Yu, J.Y., Dickson, B.J., and Jefferis, G.S.X.E. (2010). Sexual dimorphism in the fly brain. Curr Biol *20*, 1589–601.

Cardona, A., Saalfeld, S., Arganda, I., Pereanu, W., Schindelin, J., and Hartenstein, V. (2010). Identifying neuronal lineages of Drosophila by sequence analysis of axon tracts. J Neurosci *30*, 7538–53.

Chiang, A.S., Lin, C.Y., Chuang, C.C., Chang, H.M., Hsieh, C.H., Yeh, C.W., Shih, C.T., Wu, J.J., Wang, G.T., Chen, Y.C., Wu, C.C., Chen, G.Y., Ching, Y.T., Lee, P.C., Lin, C.Y., Lin, H.H., Wu, C.C., Hsu, H.W., Huang, Y.A., Chen, J.Y., Chiang, H.J., Lu, C.F., Ni, R.F., Yeh, C.Y., and Hwang, J.K. (2011). Three-dimensional reconstruction of brain-wide wiring networks in Drosophila at single-cell resolution. Curr Biol *21*, 1–11.

Conte, D., Foggia, P., Sansone, C., and Vento, M. (2004). Thirty years of graph matching in pattern recognition. International journal of pattern recognition and artificial intelligence *18*, 265–298.

Doube, M., Kłosowski, M.M., Arganda-Carreras, I., Cordelières, F.P., Dougherty, R.P., Jackson, J.S., Schmid, B., Hutchinson, J.R., and Shefelbine, S.J. (2010). BoneJ: Free and extensible bone image analysis in ImageJ. Bone *47*, 1076 – 1079.

Evers, J.F., Schmitt, S., Sibila, M., and Duch, C. (2005). Progress in functional neuroanatomy: precise automatic geometric reconstruction of neuronal morphology from confocal image stacks. J Neurophysiol *93*, 2331–42.

Frey, B.J., and Dueck, D. (2007). Clustering by passing messages between data points. Science *315*, 972–976.

Ganglberger, F., Schulze, F., Tirian, L., Novikov, A., Dickson, B., Bühler, K., and Langs, G. (2014). Structure-Based Neuron Retrieval Across Drosophila Brains. Neuroinformatics .

Ito, K., Shinomiya, K., Ito, M., Armstrong, J.D., Boyan, G., Hartenstein, V., Harzsch, S., Heisenberg, M., Homberg, U., Jenett, A., et al. (2014). A systematic nomenclature for the insect brain. Neuron *81*, 755–765.

Jefferis, G.S.X.E., Potter, C.J., Chan, A.M., Marin, E.C., Rohlfing, T., Maurer, C.R.J., and Luo, L. (2007). Comprehensive maps of Drosophila higher olfactory centers: spatially segregated fruit and pheromone representation. Cell *128*, 1187–1203.

Jenett, A., Rubin, G.M., Ngo, T.T., Shepherd, D., Murphy, C., Dionne, H., Pfeiffer, B.D., Cavallaro, A., Hall, D., Jeter, J., et al. (2012). A GAL4-Driver Line Resource for Drosophila Neurobiology. Cell Rep *2*, 991–1001.

Khoshelham, K. (2007). Extending Generalized Hough Transform to Detect 3D Objects in Laser Range Data. In ISPRS Workshop on Laser Scanning, Proceedings, LS 2007. pp. 206–210.

Kimura, K.i., Hachiya, T., Koganezawa, M., Tazawa, T., and Yamamoto, D. (2008). Fruitless and doublesex coordinate to generate male-specific neurons that can initiate courtship. Neuron *59*, 759–769.

Kimura, K.I., Ote, M., Tazawa, T., and Yamamoto, D. (2005). Fruitless specifies sexually dimorphic neural circuitry in the Drosophila brain. Nature *438*, 229–233.

Koganezawa, M., Haba, D., Matsuo, T., and Yamamoto, D. (2010). The Shaping of Male Courtship Posture by Lateralized Gustatory Inputs to Male-Specific Interneurons. Current Biology *20*, 1–8.

Kohl, J., Huoviala, P., and Jefferis, G.S. (2015). Pheromone processing in Drosophila. Curr Opin Neurobiol *34*, 149–157.

Lai, J.S.Y., Lo, S.J., Dickson, B.J., and Chiang, A.S. (2012). Auditory circuit in the Drosophila brain. Proc Natl Acad Sci U S A *109*, 2607–12.

Le, Q., Ranzato, M., Monga, R., Devin, M., Chen, K., Corrado, G., Dean, J., and Ng, A. (2012). Building high-level features using large scale unsupervised learning. In International Conference in Machine Learning.

Manton, J.D., Ostrovsky, A.D., Goetz, L., Costa, M., Rohlfing, T., and Jefferis, G.S.X.E. (2014). Combining genome-scale Drosophila 3D neuroanatomical data by bridging template brains. bioRxiv .

Masse, N.Y., Cachero, S., Ostrovsky, A., and Jefferis, G.S.X.E. (2012). A mutual information approach to automate identification of neuronal clusters in Drosophila brain images. Frontiers in Neuroinformatics *6*.

Mayerich, D., Bjornsson, C., Taylor, J., and Roysam, B. (2012). NetMets: software for quantifying and visualizing errors in biological network segmentation. BMC Bioinformatics *13 Suppl 8*, S7.

Otsuna, H., and Ito, K. (2006). Systematic analysis of the visual projection neurons of Drosophila melanogaster. I. Lobula-specific pathways. J Comp Neurol *497*, 928–958.

Panser, K., Tirian, L., Schulze, F., Villalba, S., Jefferis, G.S., Buehler, K., and Straw, A.D. (2015). Automatic segmentation of Drosophila neural compartments using GAL4 expression data reveals novel visual pathways. bioRxiv .

Peng, H., Tang, J., Xiao, H., Bria, A., Zhou, J., Butler, V., Zhou, Z., Gonzalez-Bellido, P.T., Oh, S.W., Chen, J., Mitra, A., Tsien, R.W., Zeng, H., Ascoli, G.A., Iannello, G., Hawrylycz, M., Myers, E., and Long, F. (2014). Virtual finger boosts three-dimensional imaging and microsurgery as well as terabyte volume image visualization and analysis. Nat Commun *5*, 4342.

Rohlfing, T., and Maurer, C. R., J. (2003). Nonrigid image registration in shared-memory multiprocessor environments with application to brains, breasts, and bees. IEEE Trans Inf Technol Biomed *7*, 16–25.

Rueckert, D., Sonoda, L.I., Hayes, C., Hill, D.L., Leach, M.O., and Hawkes, D.J. (1999). Nonrigid registration using free-form deformations: application to breast MR images. IEEE Trans Med Imaging *18*, 712–21.

Tanaka, N.K., Endo, K., and Ito, K. (2012). Organization of antennal lobe-associated neurons in adult Drosophila melanogaster brain. J Comp Neurol *520*, 4067–4130.

Tanaka, N.K., Tanimoto, H., and Ito, K. (2008). Neuronal assemblies of the Drosophila mushroom body. J Comp Neurol *508*, 711–755.

Wan, Y., Long, F., Qu, L., Xiao, H., Hawrylycz, M., Myers, E.W., and Peng, H. (2015). BlastNeuron for Automated Comparison, Retrieval and Clustering of 3D Neuron Morphologies. Neuroinformatics .

Yu, J.Y., Kanai, M.I., Demir, E., Jefferis, G.S.X.E., and Dickson, B.J. (2010). Cellular organization of the neural circuit that drives Drosophila courtship behavior. Curr Biol *20*, 1602–14.

Zhu, S., Chiang, A.S., and Lee, T. (2003). Development of the Drosophila mushroom bodies: elaboration, remodeling and spatial organization of dendrites in the calyx. Development *130*, 2603–2610.
