# Supplementary material for: NBLAST: Rapid, Sensitive Comparison of Neuronal Structure and Construction of Neuron Family Databases
Source: Neuron. 2016 Jul 20;91(2):293–311. doi: 10.1016/j.neuron.2016.06.012 (PMC4961245; doi:10.1016/j.neuron.2016.06.012)
Supplement: Document S2. Article plus Supplemental Information [file mmc5.pdf]

Neuron

NeuroResource

NBLAST: Rapid, Sensitive Comparison of Neuronal Structure and Construction of Neuron Family Databases

Highlights

- NBLAST is a fast and sensitive algorithm to measure pairwise neuronal similarity
- NBLAST can distinguish neuronal types at the finest level without training
- Automated clustering of 16,129 *Drosophila* neurons identifies 1,052 classes
- Online search tool for databases of single neurons or genetic driver lines

Authors

Marta Costa, James D. Manton, Aaron D. Ostrovsky, Steffen Prohaska, Gregory S.X.E. Jefferis

Correspondence

jefferis@mrc-lmb.cam.ac.uk

In Brief

Thousands of single-neuron images are being generated by efforts to map circuits and define neuronal types. Costa et al. validate a new neuronal similarity algorithm, NBLAST, demonstrating that it can distinguish neuronal types and organize huge datasets.

Costa et al., 2016, Neuron *91*, 293–311
July 20, 2016 © 2016 MRC Laboratory of Molecular Biology.
Published by Elsevier Inc.
http://dx.doi.org/10.1016/j.neuron.2016.06.012

NBLAST: Rapid, Sensitive Comparison of Neuronal Structure and Construction of Neuron Family Databases

Marta Costa,[1,2] James D. Manton,[1,5] Aaron D. Ostrovsky,[1,6] Steffen Prohaska,[1,3] and Gregory S.X.E. Jefferis[1,4,*]
[1]Neurobiology Division, MRC Laboratory of Molecular Biology, Cambridge CB2 0QH, UK
[2]Department of Genetics, University of Cambridge, Cambridge CB2 3EH, UK
[3]Zuse Institute Berlin (ZIB), 14195 Berlin-Dahlem, Germany
[4]Department of Zoology, University of Cambridge, Cambridge CB2 3EJ, UK
[5]Present address: Department of Chemical Engineering and Biotechnology, University of Cambridge, Cambridge CB2 3RA, UK
[6]Present address: Centre for Organismal Studies, Heidelberg University, Heidelberg D-69120, Germany
*Correspondence: jefferis@mrc-lmb.cam.ac.uk
http://dx.doi.org/10.1016/j.neuron.2016.06.012

SUMMARY

Neural circuit mapping is generating datasets of tens of thousands of labeled neurons. New computational tools are needed to search and organize these data. We present NBLAST, a sensitive and rapid algorithm, for measuring pairwise neuronal similarity. NBLAST considers both position and local geometry, decomposing neurons into short segments; matched segments are scored using a probabilistic scoring matrix defined by statistics of matches and non-matches. We validated NBLAST on a published dataset of 16,129 single *Drosophila* neurons. NBLAST can distinguish neuronal types down to the finest level (single identified neurons) without a priori information. Cluster analysis of extensively studied neuronal classes identified new types and unreported topographical features. Fully automated clustering organized the validation dataset into 1,052 clusters, many of which map onto previously described neuronal types. NBLAST supports additional query types, including searching neurons against transgene expression patterns. Finally, we show that NBLAST is effective with data from other invertebrates and zebrafish.

INTRODUCTION

Correlating the functional properties and behavioral relevance of neurons with their cell type is a basic activity in neural circuit research. While there is no universally accepted definition of neuron type, key descriptors include morphology, position within the nervous system, genetic markers, connectivity, and intrinsic electrophysiological signatures (Migliore and Shepherd, 2005; Bota and Swanson, 2007; Rowe and Stone, 1977). Despite this ambiguity, neuron type is a key abstraction, helping to reveal organizational principles and enabling results to be compared and collated across research groups. There is increasing appreciation that highly quantitative approaches are critical to generate cell-type catalogs in support of circuit research (Ascoli et al., 2008; Nelson et al., 2006; Kepecs and Fishell, 2014) (http://acd.od.nih.gov/presentations/brain-interim-report.pdf).

Since neuronal morphology and position strongly constrain connectivity, they have been mainstays of circuit studies for over a century. Classic morphological techniques include the Golgi method used by Cajal, microinjection, and intracellular fills during recording. Recently, genetic approaches to sparse and combinatorial labeling have enabled increasingly large-scale characterization of single-neuron morphology (Jefferis and Livet, 2012).

Classically, the position of neuronal somata or arbors was established relative to anatomical landmarks, revealed by a general counterstain; this is especially effective in brain regions with strong laminar organization, e.g., cerebellum (Cajal, 1911), retina (e.g., Badea and Nathans, 2004; Kong et al., 2005; Sümbül et al., 2014), or fly optic lobe (Fischbach and Dittrich, 1989; Morante and Desplan, 2008). Recently, 3D light microscopy and image registration have enabled direct image fusion to generate digital 3D atlases of brain regions or whole brains (Jefferis et al., 2007; Lin et al., 2007; El Jundi et al., 2010; Rybak et al., 2010; Cachero et al., 2010; Yu et al., 2010b; Sunkin et al., 2013; Zingg et al., 2014; Oh et al., 2014). Atlases can generate specific, testable hypotheses about circuit organization and connectivity at large scales. For example, Chiang et al. (2011) combined genetic mosaic labeling and image registration to produce an atlas of over 16,000 single cells embedded within a standard *Drosophila* brain (FlyCircuit dataset).

Neuronal morphologies can be represented as directed graph structures embedded in 3D space; usually this is the (arbitrary) physical space of the imaging system, rather than a brain atlas. For this reason, databases such as NeuroMorpho.org (Parekh and Ascoli, 2013) contain >37,500 neurons but omit precise positional information. Data on this scale present both an acute challenge, finding and organizing related neurons, but also an opportunity: quantitative morphology may help solve the problem of defining cell type. A key requirement is a tool enabling rapid and sensitive computation of neuronal similarity within

This is an open access article under the CC BY license (http://creativecommons.org/licenses/by/4.0/).

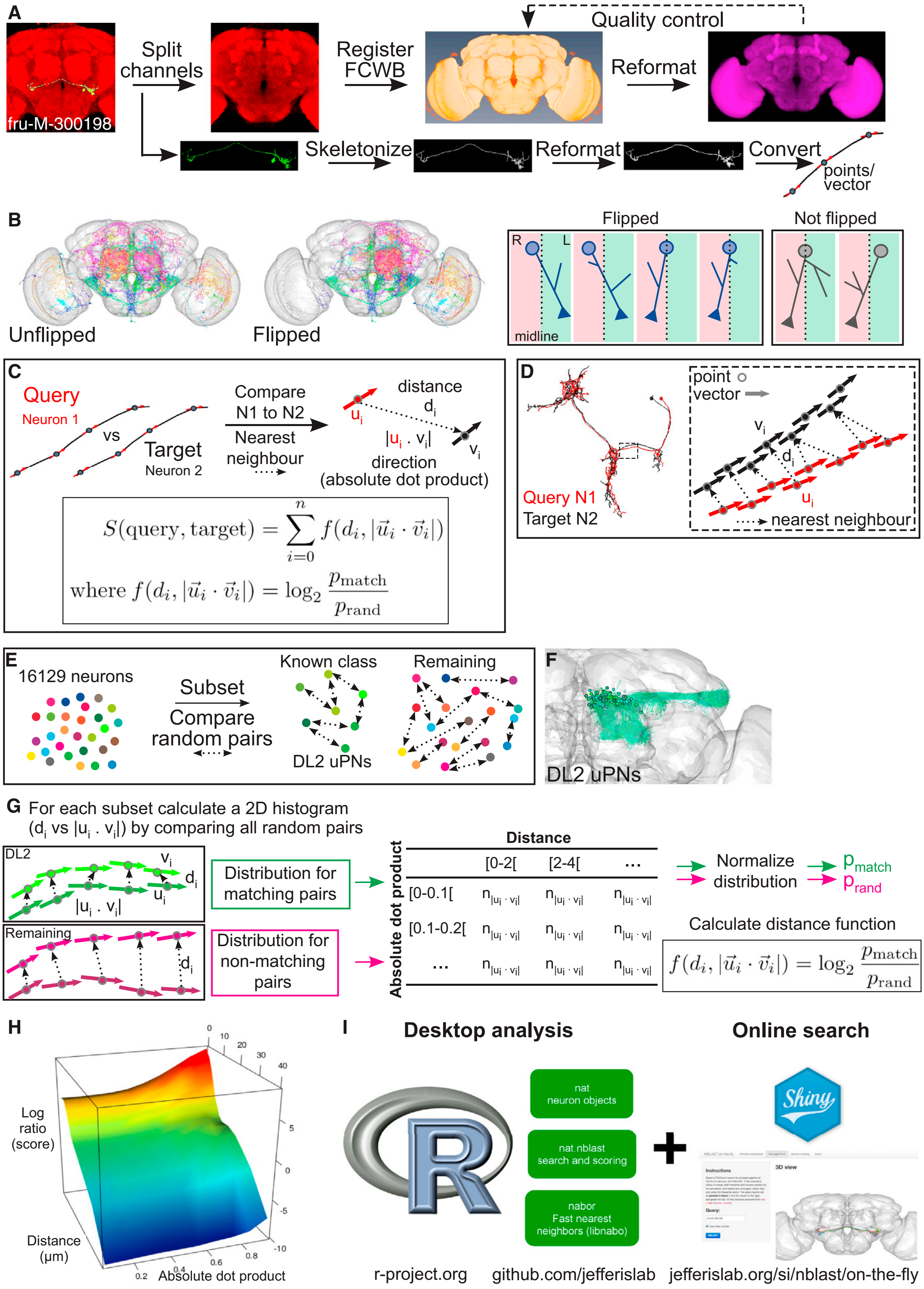

(legend on next page)

and between datasets. This has clear analogies with bioinformatics: the explosion of biological sequence information from the late 1980s motivated the development of sequence similarity tools such as BLAST (Altschul et al., 1990), enabling rapid database queries as well as hierarchically organized protein family databases.

Several strategies for measuring neuronal similarity exist with distinct target applications and different underlying data structures (Cardona et al., 2010; Basu et al., 2011; Mayerich et al., 2012; Ganglberger et al., 2014; Wan et al., 2015). For example, Cardona et al. (2010) developed an elegant approach to match curves in space and validated this on a few hundred traced structures; however, this algorithm treats each unbranched neuronal segment as a separate alignment problem, so there is no natural way to handle trees with many such segments. Recently, Wan et al. (2015) developed a sophisticated approach that combines graph matching with 3D positional information for sensitive global alignment of fully branched neurons, but this carries a significant computational cost (minutes per pair of neurons).

Our own approach started with a simple but flexible representation of neurons as point clouds with vectors defining the local heading of individual processes (Masse et al., 2012). We found that this can be efficiently computed for single-neuron data refractory to automated tracing (Peng et al., 2015), as well as more complex expression patterns. Combining this representation with a very large single-neuron dataset (Chiang et al., 2011) allowed us to validate a new algorithm, NBLAST, that is flexible, extremely sensitive, and very fast (pairwise search times of 2 ms on a laptop). Critically, the algorithm's scoring parameters are defined statistically rather than by expert intuition, but generalize across neuronal classes.

We first describe the NBLAST algorithm, providing an open source implementation in R and a web query tool. We validate NBLAST for applications including neuron database search, unsupervised clustering, and expression pattern search. NBLAST can identify well-studied neuronal types in *Drosophila* with sensitivity matching domain experts, in a fraction of the time. NBLAST can also identify new neuronal types and reveal undescribed features of topographic organization. Finally, we apply our method to 16,129 neurons from the FlyCircuit dataset, reducing this to a non-redundant set of 1,052 morphological clusters. Manual evaluation of a subset of clusters shows they closely match expert definition of cell types. These clusters, which we organize into an online supercluster hierarchy, represent a preliminary global cell type classification for the *Drosophila* brain.

RESULTS

Algorithm

Our goal was to develop a neuron similarity algorithm depending on both spatial location (within a brain or brain region) and branching pattern that was both extremely sensitive and very fast. We envisaged searching large databases of neurons (10,000–100,000 neurons), clustering neurons into families by calculating all-against-all similarity matrices, and efficient navigation of such large datasets. We eventually selected an approach based on direct pairwise comparison of neurons pre-registered to a template brain and represented as vector clouds (further details in Supplemental Information, available online).

The starting point for our algorithm is to break neurons into short segments, each characterized by a location and tangent vector. This retains local geometry, but not the topology of the neuron's branching structure. This simplified representation can be constructed for image data that would not permit automated reconstructions. To prepare such data in quantity, we developed an image processing pipeline summarized in Figure 1A (see Experimental Procedures). Briefly, brain images from the FlyCircuit dataset (Chiang et al., 2011) were subjected to non-rigid image registration (Jefferis et al., 2007) to a new intersex template brain. Neuron images were thresholded and skeletonized (Lee et al., 1994) using Fiji (Schindelin et al., 2012), thresholded images were converted to the point and tangent vector (i.e., local heading) representation (Masse et al., 2012) using our R package nat (Jefferis and Manton, 2014), and tangent vectors were computed as the first eigenvector of a singular value decomposition (SVD) of each point and its five nearest neighbors.

After preprocessing, 3D data were visualized and analyzed in R (Figure 1B). Neurons had a median of 1,070 points/vectors; the 16,129 neurons occupied 1.8 GB, fitting comfortably in a laptop's main memory. Since the fly brain is almost completely symmetric, we mapped all neurons to the left hemisphere (defined

Figure 1. Image Preprocessing, Registration, and NBLAST Algorithm

(A) Flowchart describing the image preprocessing and registration procedure. FlyCircuit images were split into two channels. The Dlg-stained brain (Discs large) images were registered against the FCWB template. Successful registrations were applied to neuron skeletons converted into points and vectors.

(B) Neurons in the right hemisphere were flipped to the left. Brain plots show 50 random neurons before and after flipping. On the right, cases for which the neuron flipping was assessed manually.

(C) NBLAST algorithm. The similarity of two neurons (query and target) is a function of the distance and absolute dot product between nearest-neighbor segments of the query/target pair. This function reflects the probability of a match between a pair of segments (pmatch) relative to a random pair (prand).

(D) Diagram illustrating how nearest-neighbor points are calculated. For a query (N1)/target (N2) pair, each point of N1 (u_i) is matched to a point in N2 (v_i), minimizing the distance (d_i).

(E) Defining the scoring function. Random pairs of neurons within two groups, DL2 uPNs and all remaining neurons, were compared.

(F) Brain plot of DL2 uPNs.

(G) Calculation of the distribution for matching and non-matching pairs of segments. For all segment pairs of all neuron pairs in each group, the distance and a 2D histogram were calculated for absolute dot product (10 bins) and distance (21 bins). These histograms were converted to joint probability densities for matching (pmatch) or non-matching pairs (prand) by normalizing the distance histogram to sum to 1.

(H) Plot showing that similarity score depends on distance between points and the vector direction (absolute dot product).

(I) Summary diagram of the desktop and online NBLAST implementation.

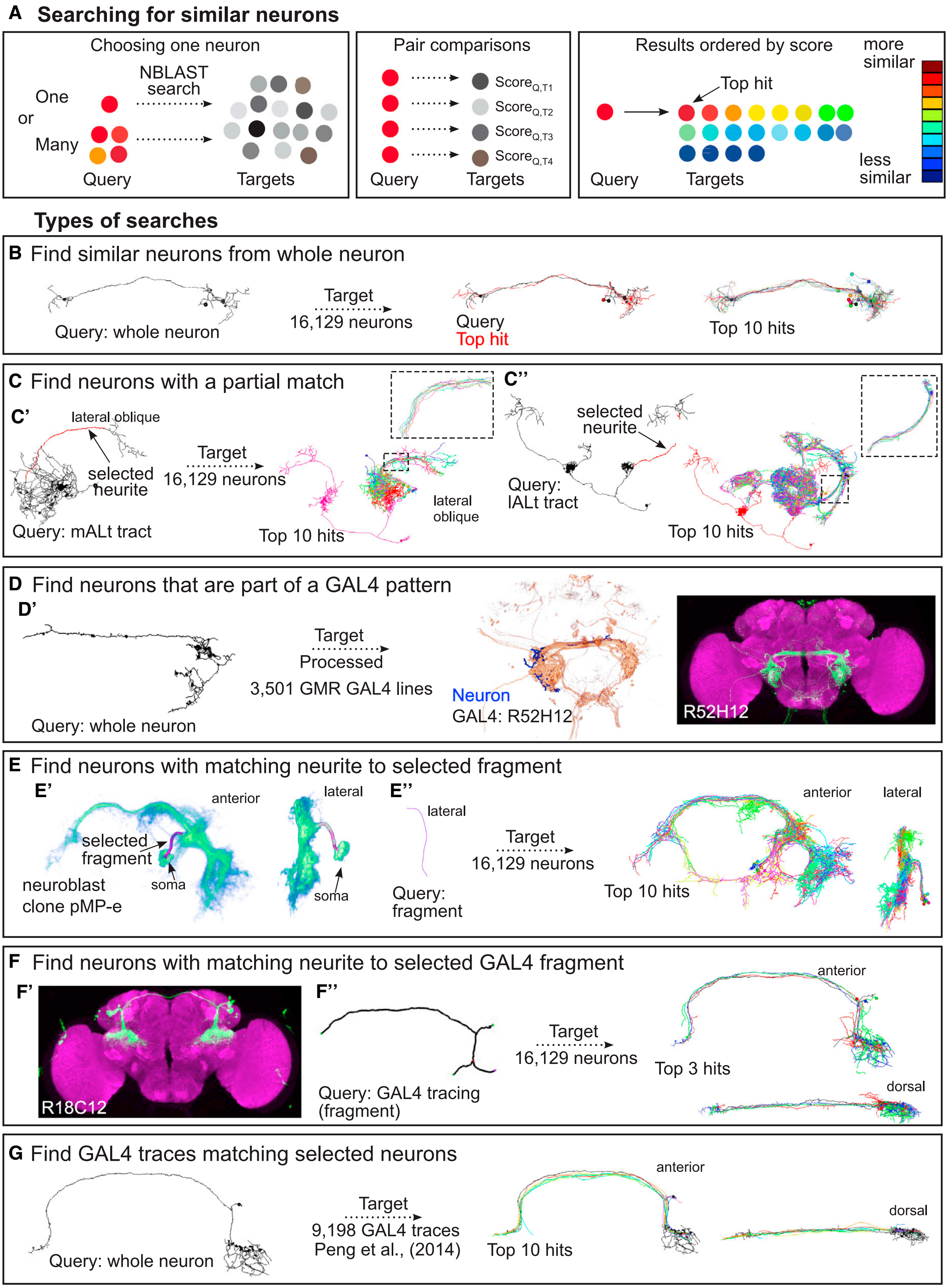

(legend on next page)

primarily by cell body location; see Experimental Procedures and Figure 1B) using a non-rigid mirroring procedure (Manton et al., 2014).

We then calculated NBLAST pairwise similarity scores using this database of preprocessed, aligned neurons. For a given query and target neuron, we iterate over each segment in the query neuron, identifying the nearest neighbor (Euclidean distance) in the target neuron (Figures 1C and 1D). The score for the segment pair is a function of two measurements: d_i, the distance between matched segments (indexed by i), and $\left|\vec{u_i}\cdot\vec{v_i}\right|$, the absolute dot product of the two tangent vectors; the absolute dot product is used because the head-to-tail orientation of tangent vectors is arbitrary (Figure 1C). The scores are then summed over each segment pair to give a raw score, S:

$$S(\text{query}, \text{target}) = \sum_{i=1}^{n} f\left(d_i, \left|\vec{u_i}\cdot\vec{v_i}\right|\right). \qquad \text{(Equation 1)}$$

The next question is, what is an appropriate function $f(d_i, \left|\vec{u_i}\cdot\vec{v_i}\right|)$? Our approach was inspired by the BLAST scoring system (Altschul et al., 1990). For each segment pair, we defined the score as the log probability ratio,

$$f = \log_2 \frac{p_{\text{match}}}{p_{\text{rand}}}, \qquad \text{(Equation 2)}$$

i.e., the probability that the segment pair was derived from a pair of neurons of the same type, versus a pair of unrelated neurons. We then defined p_{match} empirically using the joint distribution of d and $\left|\vec{u_i}\cdot\vec{v_i}\right|$ for pairs of neurons of the same type (Figures 1E–1G); we used 150 olfactory projection neurons (PNs) innervating the same glomerulus, therefore unambiguously the same neuronal type (Figure 1F). p_{rand} was calculated by drawing 5,000 random pairs of neurons from the database, assuming that the large majority of such pairs are unrelated neurons. Joint distributions were calculated for both groups and normalized to convert them to probabilities, and the log ratio defined the final scoring matrix (Figure 1G). Plotting the scoring matrix emphasizes the strong distance dependence of the score but also shows that for segment pairs closer than ~10 μm, the logarithm of the odds score increases markedly as the absolute dot product moves from 0 to 1 (Figure 1H).

We implemented the NBLAST algorithm as an R package (nat.nblast), building on a high-performance k-nearest neighbor library (nabor), that immediately enables pairwise queries, searches of a single query neuron against a database of target neurons (Figure 2), and all-by-all searches (Figure 1I). Runtimes on a single core laptop computer were 2 ms per comparison or 30 s for all 16,129 neurons. In order to enable interactive neuron clustering, we also pre-computed an all-by-all similarity matrix for all 16,129 neurons (2.6×10^8 scores, 1.0 GB). We also developed a simple web application (see jefferislab.org/si/nblast), enabling online queries of this test dataset (Figure 1I).

NBLAST Finds Whole or Partial Matches for Diverse Query Objects

NBLAST is flexible, identifying both global and partial matches for multiple classes of queries (Figure 2). The only requirements are that these objects (or fragments) must be registered against a template brain and converted to a point and vector representation.

Our first example queries a (whole) FlyCircuit neuron against 16,129 FlyCircuit neurons. The top hits are very similar neurons with small differences in length and neurite position (Figure 2B). Using this search type, we identified Kenyon cell (KC), olfactory projection, and auditory neuron classes and subclasses, known and new (Figures 4 and S6). A second example uses an axon fragment; all top hits follow the same axon tract, but their variable axonal and dendritic arbors define distinct neuron types (Figure 2C). NBLAST searches using a neuron fragment could identify known and new visual projection and mAL neuron types (Figures 5, S5, and 6). In a third example, we query against 3,501 FlyLight GAL4 driver lines (Jenett et al., 2012), finding lines that contain the query neuron (Figure 2D).

User tracings can also be used as queries. We traced the characteristic bundle of 20–30 primary neurites of the *fruitless* neuroblast clone pMP-e that generates male-specific P1 neurons (Kimura et al., 2008; Cachero et al., 2010). This returned many P1 neurons (Figure 2E), identifying new subtypes likely to have distinct functions in male behavior (Figure 6). A similar approach can be used to identify candidate neuronal types labeled by genetic driver lines even when the detailed morphology of individual neurons cannot be determined: we traced the main neurites of a cell cluster in a GAL4 line (Jenett et al., 2012) (Figure 2F) and used that trace as the query. NBLAST identified three very similar FlyCircuit neurons, which completely overlapped with the GAL4 expression pattern. These three

Figure 2. NBLAST Allows Different Search Types

(A) Searching for neurons with NBLAST. Pairwise scores between a query and target neurons produce a ranked result set.

(B) NBLAST search using a whole query neuron against the FlyCircuit dataset. The query (fru-M-400121), top hit, and top ten hits in anterior view.

(C) NBLAST search using a neuron fragment against the FlyCircuit dataset. The query and top ten hits are shown.

(C′) Search with the mALT tract from an olfactory PN (Cha-F-000239). Lateral oblique view; inset shows mALT tract for top ten hits.

(C″) Search with lALT tract from an olfactory PN (Gad1-F-200095). Anterior view; inset shows lALT tract of top ten hits.

(D) NBLAST search of neuron against FlyLight GAL4 lines.

(D′) From left to right: query neuron (Trh-M-300069), volume rendering of query and best GAL4 hit (R52H12), and maximum Z projection of hit.

(E) NBLAST search for FlyCircuit neurons matching a fragment from a *fruitless* neuroblast clone (pMP-e).

(E′) Volume rendering of pMP-e clone with the traced fragment (anterior and lateral views).

(E″) Query fragment in lateral view. Top ten hits in anterior and lateral view.

(F) NBLAST search for FlyCircuit neurons matching fragment traced from GAL4 image (R18C12) (Jenett et al., 2012).

(F′) Maximum Z projection of line R18C12.

(F″) The query fragment traced in Vaa3D (anterior view). Top three hits (anterior and dorsal views).

(G) GAL4 traces (Peng et al., 2014) matching selected FlyCircuit neuron (VGlut-F-500818). The top ten trace hits are shown (anterior and dorsal views).

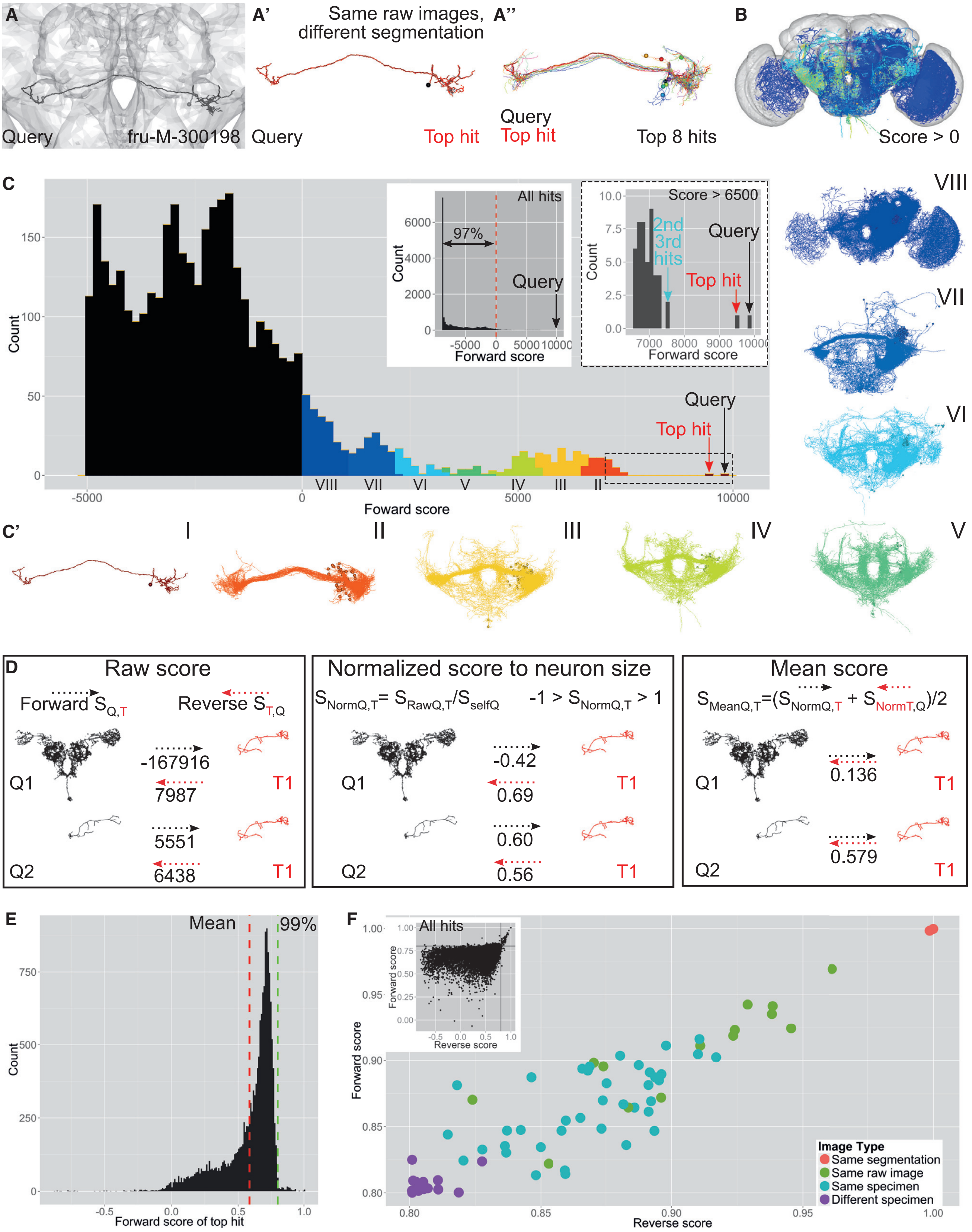

(legend on next page)

neurons appear to be different subtypes, each varying in their terminal arborizations. Conversely, we used one tracing from a published projectome dataset containing >9,000 neurite fibers (Peng et al., 2014) to find similar FlyCircuit neurons (Figure 2G).

NBLAST Scores Are Sensitive and Biologically Meaningful

A good similarity algorithm should be sensitive enough to reveal identical neurons with certainty, while having the specificity to ensure that all high-scoring results are relevant. We used the full FlyCircuit dataset to validate NBLAST performance.

Our first example uses an auditory interneuron, fru-M-300198, as query (Figures 3A–3C). The highest NBLAST score was the query neuron itself (it is present in the database), followed by the top hit (fru-M-300174), which completely overlaps with the query (Figure 3A′). A histogram of NBLAST scores showed that the top hit was clearly an outlier, scoring 96.1% compared to the self-match score of the query neuron (Figure 3C). Further investigation revealed that these "identical twins," both derived from the same raw confocal image, were likely the result of a data entry error. The next eight hits are also very similar to the query but are clearly distinct specimens, having small differences in position, length, and neurite branching that are typical of sister neurons of the same type (Figure 3A″).

The score histogram shows that only a minority of hits (3%) have a score above 0 (Figures 3B and 3C). A score of 0 represents a natural cutoff for NBLAST, since it means that, on average, segment pairs from this query and target neuron have a similarity level that is equally likely to have arisen from a random pair of neurons in the database as a pair of neurons of the same type. We divided the neurons with score >0 into 8 groups with decreasing similarity scores (Figure 3C′). Only the highest-scoring real hits (group II) appear to be of exactly the same type, although lower-scoring groups contain neurons that would be ranked as very similar.

Although raw NBLAST scores correctly identify similar neurons, they are not comparable from one query neuron to the next: the score depends on neuron size and segment number. This confounds search results for neurons of very different sizes or when the identity of query and target neurons is reversed. For example, a search with a large neuron as query and a smaller one as target (pair 1) will have a very low forward score because the large neuron has many unmatched segments, but a high reverse score, since most of target will match part of the query (Figure 3D). One approach to correct for this is to normalize the scores by the size of the query neuron. Although normalized scores are comparable, unequal forward and reverse scores between large and small neurons remain an issue. One simple strategy is to calculate the mean of the forward and reverse scores (mean score). Two neurons of similar size have a higher mean score than two neurons of unequal size (Figure 3D). Repeating the analysis of Figures 3C and 3C′ using mean scores (Figure S2) eliminated some false matches due to unequal size.

During our analysis, we sporadically noticed cases where two database images were derived from the same physical specimen (Figure S1). We tested if NBLAST could identify these instances. We collected the top hit for each neuron and analyzed the distribution of forward (Figure 3E) and reverse scores (data not shown). A small tail (~1% of all top hits) has anomalously high scores (>0.8). Given this distribution, we examined neuron pairs with forward and reverse scores >0.8. We classified these 72 pairs into 4 different groups. From highest to lowest predicted similarity, the groups are as follows: same segmentation, i.e., a neuron image duplicated after segmentation (Figure S1A); same raw image, resulting in different segmentations of the same neuron (Figure 3B′); same specimen, i.e., two separate confocal images from the same brain (Figure S1B); and different specimen, when two neurons are actually from different brains (but of the same neuron type). The distribution of NBLAST scores for these four categories matches the predicted hierarchy of similarity (Figure 3F). These results underline the high sensitivity of the NBLAST algorithm to small differences between neurons.

Taken together, these results validate NBLAST as a sensitive and specific tool for finding similar neurons.

NBLAST Scores Can Distinguish Kenyon Cell Classes

We next investigated whether NBLAST scores can be used to cluster neurons, potentially revealing functional classes. We began with KCs, the intrinsic neurons of the mushroom body and an intensively studied population given their key role in memory formation and retrieval (reviewed in Kahsai and Zars, 2011).

There are around 2,000 KCs in each mushroom body (Aso et al., 2009), whose axons form the medial lobe, consisting of the γ, β', and β lobes, and the vertical lobe, consisting of the α and α' lobes. The dendrites form the calyx around which cell bodies are positioned; the axon peduncle joins the calyx to the lobes (Figure 4C). Three main classes of KCs are recognized, named by the lobes they innervate: γ neurons are the first born, α'/β' neurons are generated next, and last born are α/β

Figure 3. NBLAST Scores Are Accurate and Meaningful

(A) NBLAST search with fru-M-300198 (black).

(A′) Query neuron (black) and top hit (red). The top hit is a different segmentation of the same raw confocal image.

(A″) Top eight hits have differences in neurite branching, length, and position.

(B) All hits with forward score >0, colored by score, as shown in (C).

(C) Histogram of forward scores for fru-M-300198. Only hits with scores >−5,000 are shown. Left inset shows score histogram for all hits; right inset shows zoomed view of top hits (score > 6,500). See also Figure S1.

(C′) Neurons in each of the score bins in (C).

(D) Comparison of raw, normalized, and mean score for two pairs of neurons: one of unequal (Q1, T1) and one of similar size (Q2, T1).

(E) Histogram of normalized top scores for each neuron in the whole dataset. The mean and 99th percentile are shown as dashed red and green lines, respectively.

(F) Plot of normalized reverse and forward scores for 72 pairs of neurons exceeding threshold score of 0.8. These pairs were classified into four categories of decreasing predicted similarity: same segmentation, same raw image, same specimen, and different specimen. Inset shows normalized reverse and forward scores for all top hits with threshold of 0.8 indicated by two black lines.

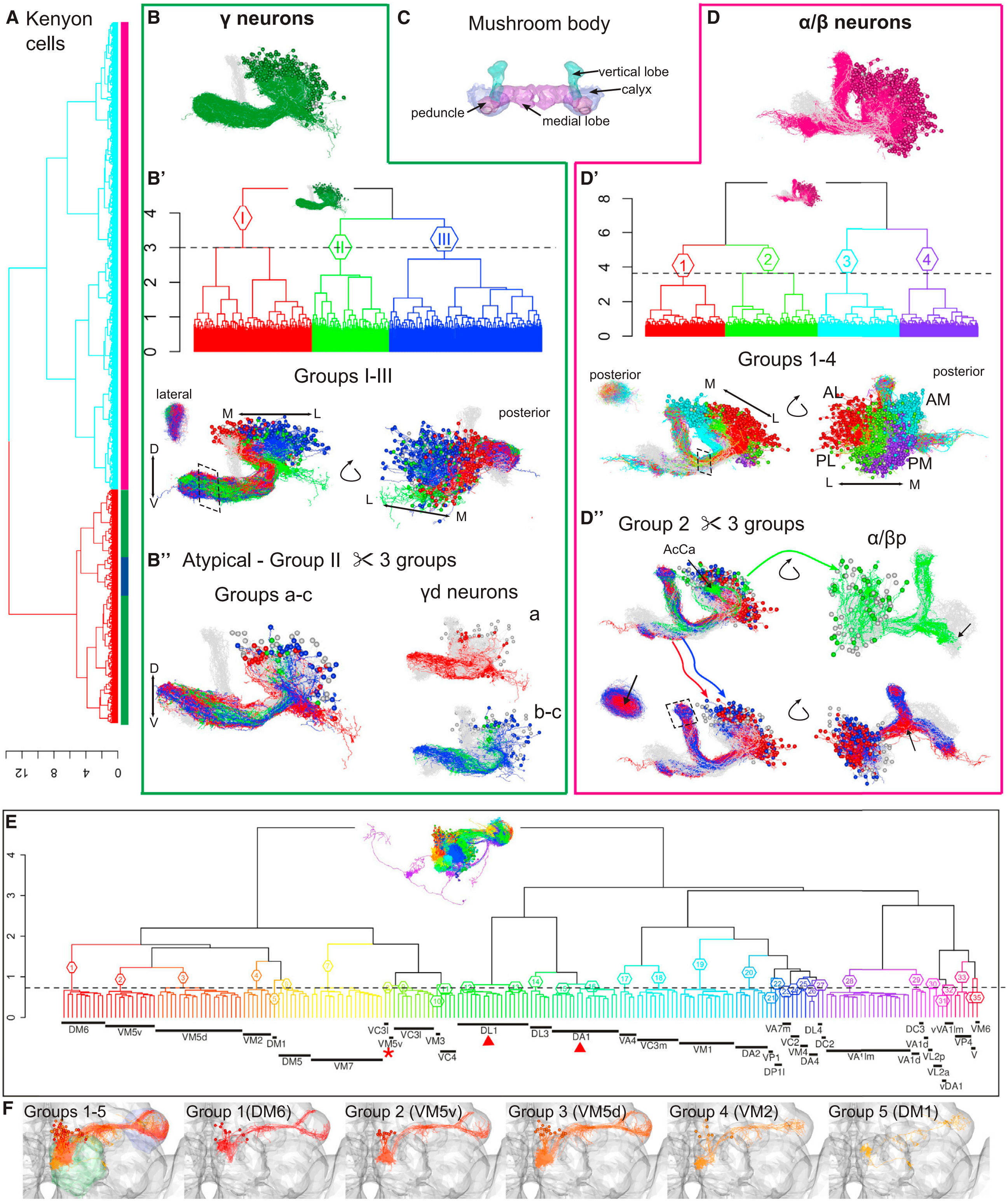

Figure 4. NBLAST Search and Clustering Reveal Kenyon Cell Subtypes

(A) Hierarchical clustering (HC) of KCs (n = 1,664). Bars below the dendrogram indicate the γ (green), α′/β′ (blue), and α/β neurons (magenta); h = 8.9.

(B) Plot of all γ neurons. KC exemplars plotted in gray for context.

(B′) HC of γ neurons (I–III); h = 3. Neuron plots of groups I–III. Lateral oblique and posterior views of neurons and lateral view of slice through horizontal lobe.

(legend continued on next page)

neurons. Four neuroblasts each generate the whole repertoire of KC types (Lee et al., 1999).

We started with a dataset of 1,664 KCs, representing 10.3% of the FlyCircuit dataset (see Supplemental Information for selection protocol), and calculated raw NBLAST scores of each KC against all others. Iterative hierarchical clustering allowed us to identify the main KC types, followed by detailed analyses that distinguished several subtypes.

For γ neurons (Figure 4B), we identified the classical neurons (Figure 4B′) (groups I and III), the recently described γd neurons (group a) (Aso et al., 2009, 2014), and two previously uncharacterized types (groups b and c) (Figure 4B′′). Analysis of α'/β' neurons highlighted the characterized subtypes of these neurons (Figure S3C), which differ in their anterior/posterior position in the peduncle and β' lobe (Tanaka et al., 2008; Aso et al., 2014).

The largest KC subset corresponds to α/β neurons (Figure 4D). We identified neurons from each of the four neuroblast lineages (Figure 4D′) (Zhu et al., 2003), and for each of these, we distinguished morphological subtypes that correlate to their birth time (Figures 4D′′ and S3D′): the last born (α/β core) inside the α lobe, the earlier (α/β) surface layer, and the earliest born (α/β posterior or pioneer) (Tanaka et al., 2008).

Hierarchical clustering of KCs using NBLAST scores therefore resolved KCs into three main types, identified the reported subtypes, and even isolated uncharacterized subtypes in an intensively studied cell population. This supports our claim that the NBLAST scores are a good metric when searching for similar neurons and organizing large datasets of related cells.

NBLAST Identifies Classic Cell Types at the Finest Level: Olfactory PNs

We have shown that clustering NBLAST scores can identify KC types. However, it remains uncertain what corresponds to an identified cell type, which we take to be the finest neuronal classification in the brain. We therefore analyzed a different neuron family, the olfactory PNs, which represent one of the best-defined cell types in the fly brain.

PNs transmit information between antennal lobe glomeruli, which receive olfactory input, and higher brain centers, including the mushroom body and the lateral horn (Masse et al., 2009). Uniglomerular PNs (uPNs) are unambiguously classified into individual types based on the glomerulus innervated by their dendrites and the axon tract they follow; these features show fixed relationships with their axonal branching patterns in higher centers and their parental neuroblast (Marin et al., 2002; Jefferis et al., 2001, 2007; Wong et al., 2002; Yu et al., 2010a; Tanaka et al., 2012).

We manually classified the 400 FlyCircuit uPNs by glomerulus (see Experimental Procedures). We found a very large number of DL2 uPNs (145 DL2d and 37 DL2v), out of 397 classified neurons. Nevertheless, our final set of uPNs broadly represents the total variability of described classes and contains neurons innervating 35 out of 56 different glomeruli (Tanaka et al., 2012), as well as examples of the three main lineage clones and tracts.

We computed mean NBLAST scores for each uPN versus the other 16,128 neurons, checking if the top hit was exactly the same uPN type, another uPN type, or a match to a different neuron class (Figure S4A). There were only eight cases in which the top hit did not match the query's type. These matches represented cases of uPNs innervating a neighboring glomerulus or multiglomerular PNs. This exercise encapsulates a very simple form of supervised learning (k-nearest neighbor with k = 1 and leave-one-out cross-validation) and shows that NBLAST scores are a useful metric, with an error rate of 2.4% for 35 classes; it is noteworthy that there was a huge amount of distracting information since uPNs represented only 2.47% of the 16,128 test neurons.

We also compared how the top three hits matched the query type (Figure S4B). For uPN types with more than three examples (non-DL2, n = 187), we collected the top three NBLAST hits for each of these neurons. We achieved very high matching rates: in 98.9% of cases (i.e., all but two), at least one of the top hits matched the query type, and all three hits matched the query type in 95.2% of cases.

Given the very high prediction accuracy, we wondered if unsupervised clustering based on NBLAST scores would group uPNs by type. To test this, we clustered uPNs (non-DL2, n = 214) and cut the dendrogram at a height of 0.725: at this level most groups corresponded to single-neuron types. For types with more than one representative neuron, all neurons co-clustered, with three exceptions (Figures 4E, 4F, and S4). The cluster organization also reflects higher-level features such as the axon tract/neuroblast of origin. Thus, unsupervised clustering of uPNs based on NBLAST scores gives an almost perfect neuronal classification: our two expert annotators took three iterative rounds of consensus-driven manual annotation to better this error rate of 1.4%.

In conclusion, these results demonstrate that morphological comparison by NBLAST is powerful enough to resolve differences

(B′′) HC of atypical γ neurons (group II in B′) divided into three groups (a–c). Neuron plots of groups a–c, a, and b and c. Group a corresponds to the γd subtype. See also Figure S3.

(C) Mushroom body neuropil and subregions.

(D) Neuron plot of α/β neurons. KC exemplars plotted in gray for context.

(D′) HC of α/β neurons divided into four groups (1–4); h = 3.64. Neuron plots of groups 1–4, which match neuroblast clones AM, AL, PM, and PL in posterior and lateral oblique views.

(D′′) HC of group 2 divided into three subgroups. Lateral oblique, posterior oblique, and dorsal view of a peduncle slice are shown. Red and blue subgroups match core and surface neurons, respectively; green subgroup corresponds to α/β posterior subtype (α/βp) (see also Figure S3D). AcCa, accessory calyx.

(E) Hierarchical clustering of uPNs (non-DL2s) (n = 214) cut into 35 groups (1–35) at h = 0.725. Dendrogram shows glomerulus for each neuron. Inset shows uPNs colored by dendrogram group. Neurons that innervate each glomerulus are indicated by black rectangles under dendrogram. Neurons originating from ventral neuroblast are indicated as vVA1lm and vDA1. Dendrogram groups correspond to unique neuron types, except for DL1 and DA1 neurons, which are split into two groups (12–13, 15–16, respectively) (red arrowhead), and the outlier neuron VM5v in group 9 (red asterisk).

(F) Neurons for groups 1–5 from (E); antennal lobe in green; lateral horn in purple. See also Figure S4.

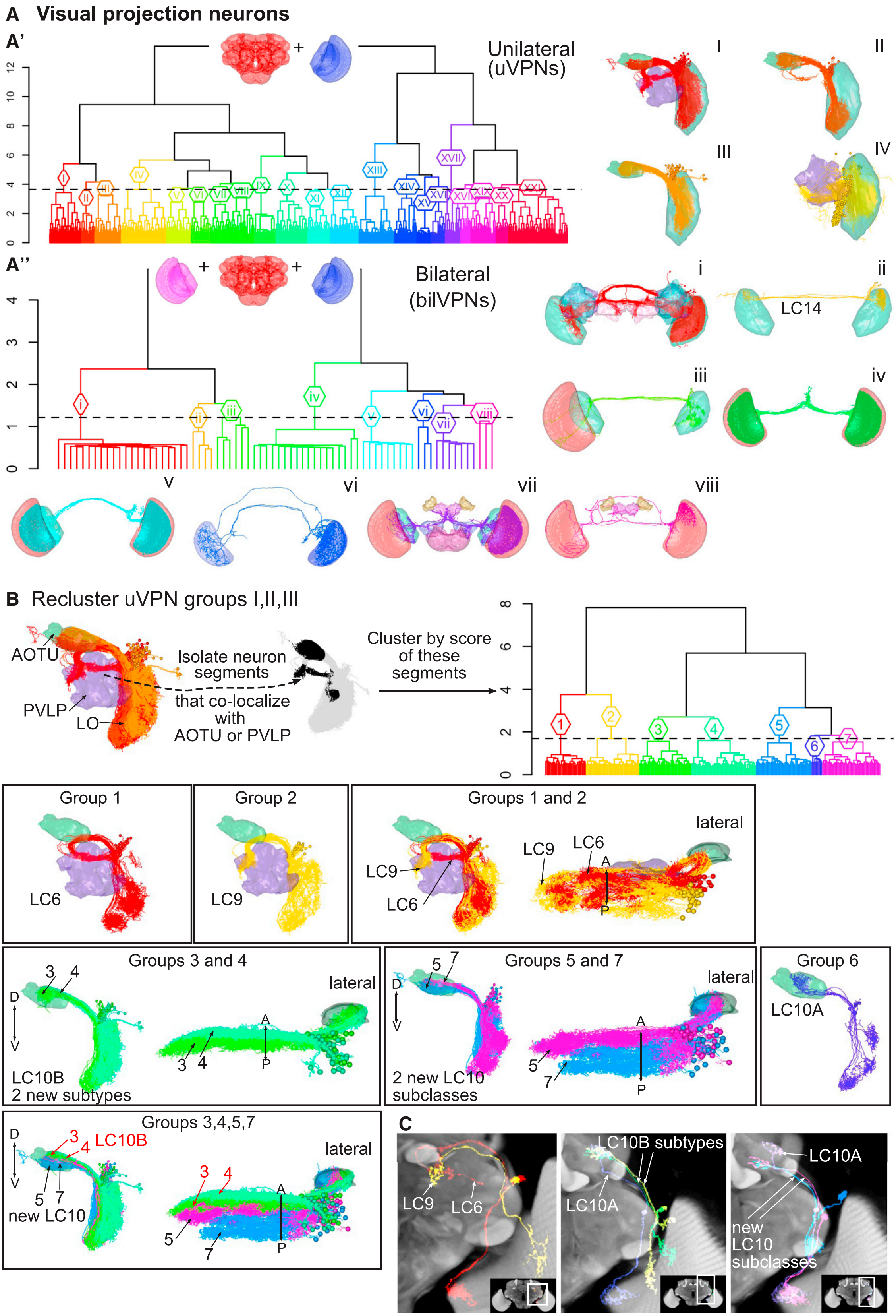

(legend on next page)

at the finest level of neuronal classification. Furthermore, they suggest that unsupervised NBLAST clustering could help reveal new neuronal types.

NBLAST Can Define New Cell Types

We wished to show the usefulness of whole and partial NBLAST searches in classifying other well-studied neuron types, and especially in identifying new cell types. We analyzed the visual PNs (VPNs), which relay information between optic lobe and the central brain (Figures 5 and S5). This is a morphologically diverse group with 44 types already described (Otsuna and Ito, 2006). We clustered FlyCircuit VPNs based only on the parts of their skeletons that overlap the central brain neuropils; this identified 11 known VPN types, 3 new subclasses, and 4 subtypes of unilateral VPNs (Table S1).

Another large and diverse neuron group is the auditory neurons. Several distinct types have been described based on anatomical and physiological features (Yorozu et al., 2009; Lai et al., 2012; Kamikouchi et al., 2006, 2009; Matsuo et al., 2016). Using simple whole-neuron searches, we were able to reveal new subtypes that differed mainly in their lateral arborizations (Figure S6; Table S2).

We also studied two classes of *fruitless*-expressing, sexually dimorphic neurons, critical for courtship behavior, the mAL (Koganezawa et al., 2010; Kimura et al., 2005) and P1 neurons (Kimura et al., 2008). We calculated NBLAST scores for partial mAL skeletons containing their axonal and dendritic arbors, clustering cleanly separated male and female neurons (Figures 6A and 6B; Supplemental Experimental Procedures), and identified three main types and two subtypes for the male neurons (Figure 6C). These male neurons include types with correlated differences in the position of input and output arbors (and likely therefore in functional connectivity). Clustering P1 neurons identified ten anatomical subtypes (Figure 6D). Nine of these contained only male neurons, each with highly distinctive patterns of dendritic and axonal arborization, suggesting that they are likely to integrate distinct sensory inputs and connect with distinct downstream targets. The last group consists only of female neurons, suggesting that a small population of female neurons shares anatomical features (and likely originates from the same neuroblast) with the male P1 neurons, key regulators of male behavior.

These analyses demonstrate that NBLAST scores for whole neurons or subregions can highlight morphological features important for defining neuron classes and provide an efficient and quantitative way to identify new cell types even for intensively studied neuronal classes.

Superclusters and Exemplars to Organize Huge Data

We have shown that NBLAST clustering can identify known and novel neuron types starting from a collection of neurons of a particular superclass (e.g., olfactory PNs). However, isolating such neuronal subsets requires considerable time. We next established a method to organize large datasets, extracting the main types automatically, retaining information on the similarity between types and subtypes, and allowing quicker navigation. We used affinity propagation clustering (Frey and Dueck, 2007), combined with hierarchical clustering, to achieve this. Applying affinity propagation to the 16,129 neurons in the FlyCircuit dataset resulted in 1,052 clusters (Figures 7A and 7B), each characterized by a single exemplar neuron. Hierarchical clustering of the exemplars and manually removing eleven stray neurons isolated the central brain neurons (groups B and C) (Figure 7C). Further hierarchical clustering of central brain exemplars revealed large superclasses of neuron types (groups I–XIV), with most containing an anatomically distinct subset, e.g., central complex neurons (I), P1 neurons (II), KCs (IV and V), and auditory neurons (VIII) (Figures 7D and 7D′). There were, however, superclusters for which the classification logic was not as clear (XI and XII, for example).

The affinity propagation clusters are also useful for identifying neuronal subtypes by comparing all clusters that contain a specified neuronal type (Figure 7E). We present examples for the neuronal types AMMC-IVLP PN 1 (AMMC-IVLP PN1) (Lai et al., 2012), and the uVPNs LC10B and LC4. For each of these, morphological differences are clear between clusters, suggesting that each one might help to identify distinct subtypes.

In short, combining affinity propagation with hierarchical clustering is an effective way to organize and explore large datasets, condensing information into a single exemplar, while retaining the ability to move up or down in the hierarchical tree, revealing broader superclasses or more narrow subtypes.

NBLAST Extensions

NBLAST is a powerful tool for working with single neurons from the adult fly; however, the algorithm was designed to be general. We now illustrate NBLAST in a wide variety of experimental contexts. We first use 40 neurons reconstructed from a complete serial section electron microscopy (EM) volume of the *Drosophila* larva. Clustering NBLAST scores recovers functional groups of neurons within a multimodal escape circuit (Figure 8A) (Ohyama et al., 2015). Pruning fine terminal branches from the EM reconstructions (mimicking light level reconstructions) has little impact on cluster assignments; therefore, NBLAST clustering of coarsely skeletonized neurons could be an important step to organize EM connectome data.

Figure 5. NBLAST Classification of Visual PNs

(A) Clustering of unilateral (uVPNs) and bilateral visual PNs (bilVPNs). Inset shows neuropils to which NBLAST was restricted. At right, plots of neuron groups, showing neuropils with most overlap. See also Figure S5.

(A′) Hierarchical clustering (HC) of uVPNs divided into 21 groups (I–XXI); h = 3.65.

(A″) HC of bilVPNs divided into 8 groups (i–viii); h = 1.22. Group ii corresponds to the LC14 neuron type (Otsuna and Ito, 2006).

(B) Reclustering of uVPN groups I, II, and III from A′. Only neuron segments within anterior optic tubercle (AOTU) or posterior ventrolateral protocerebrum (PVLP) were used for NBLAST HC. The dendrogram was cut into groups 1–7; h = 1.69. Neuron plots match dendrogram groups to known uVPN types. Group 1, LC6 neurons; group 2, LC9. Groups 3 and 4, two new LC10B subtypes. Groups 5 and 7, possible new LC10 types. Group 6, LC10A neurons. This analysis identified five subgroups of LC10 neurons, four of them not previously identified (see also Table S1 and Figure S5B).

(C) Plots of neuron skeletons with partial confocal image Z projections for selected types. White rectangle in inset shows location of close-up. LC, lobula columnar neuron.

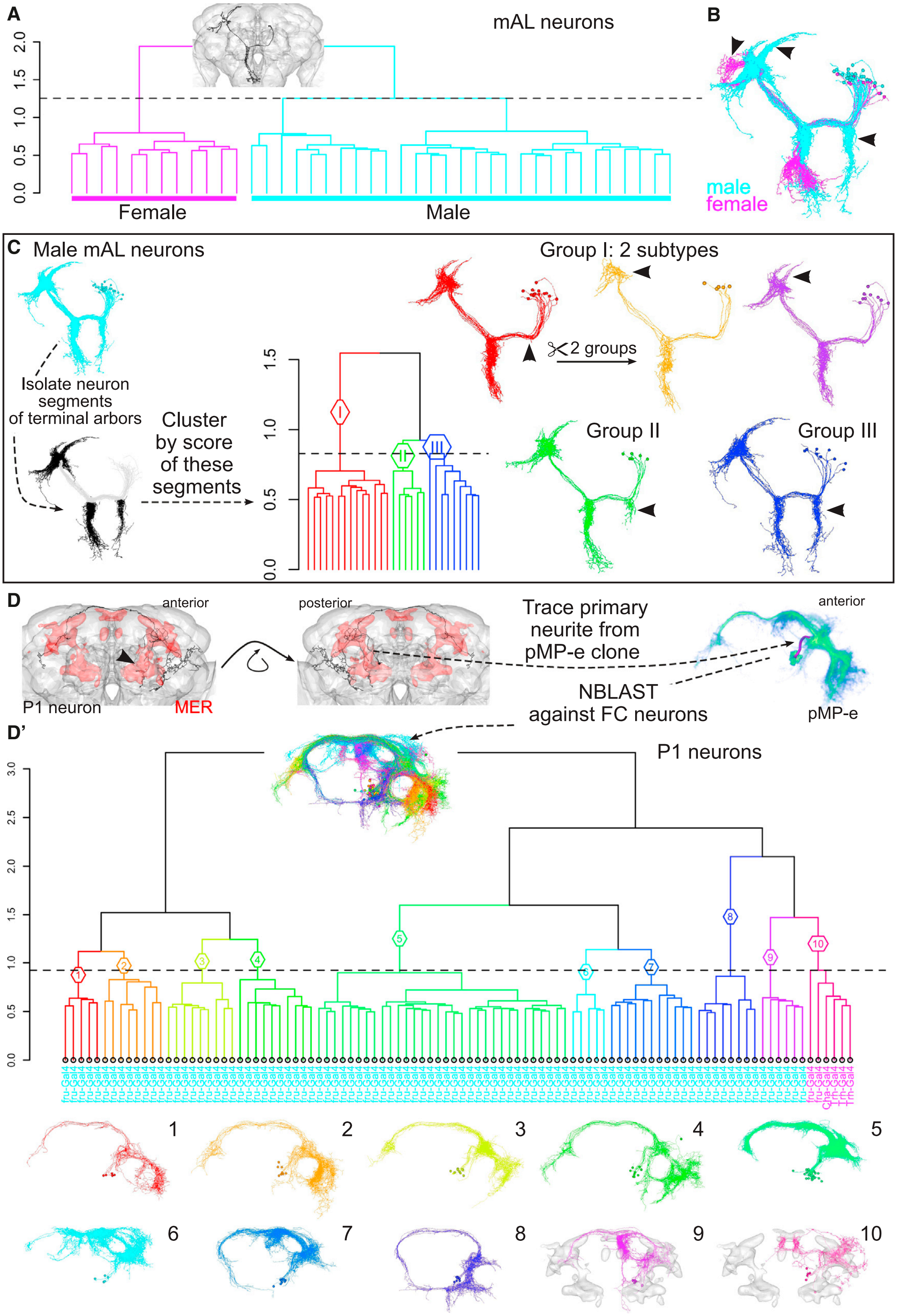

(legend on next page)

We next show two examples applying NBLAST to single-cell data from another invertebrate, the monarch butterfly, and a vertebrate, the larval zebrafish (Figures 8B and 8C). Clustering 29 monarch butterfly neurons from the central complex (Heinze et al., 2013) largely matches neuronal types defined by expert neuroanatomists—the few discrepancies were reviewed with the data provider and determined to be cases where computationally defined cell groups revealed features that were orthogonal to expert classification but still a valid classification.

The zebrafish data consisted of 55 mitral cells (second-order olfactory neurons) projecting to a variety of higher brain areas (Miyasaka et al., 2014). NBLAST clustering identified clearly distinct morphological groups (Figure 8C). Very similar neurons were co-clustered both by our algorithm and that of the original authors, but clustering of distantly related neurons was distinct. Only future experiments will show if one clustering has more functional relevance.

In our final example, we apply NBLAST to a distinct but experimentally vital form of neuroanatomical image data. Circuit neuroscience in many model organisms depends on manipulating circuit components with cell-type-specific driver lines. We have registered (Manton et al., 2014) and processed image data from the most widely used *Drosophila* collection, 3,501 GMR driver lines generated at the Janelia Research Campus (Jenett et al., 2012). We applied an image processing pipeline emphasizing tubular features (Masse et al., 2012), generating a vector cloud representation identical to that used elsewhere in this paper. These data (9 Gb for 3,501 image stacks) can be queried with single neurons or tracings in less than 30 s on a desktop computer. To demonstrate this approach at scale, we mapped GAL4 data to the same template space (Manton et al., 2014) as the FlyCircuit single neurons (merging these data in silico) and computed NBLAST scores for 16,129 neurons against 3,501 driver lines. We provide a simple web server for these queries at jefferislab.org/si/nblast/on-the-fly. We showcase this by identifying GAL4 driver lines targeting the sexually dimorphic mAL neuron population (Figures 8D and 8D′). We selected ten mAL neurons and then examined the ten GAL4 lines with the highest mean scores. The top hit line R43D01 has just been identified as targeting this population (Kallman et al., 2015), and all the top ten hits target the same population.

As a second example, we looked at driver lines labeling olfactory PNs targeting the CO_2-responsive V glomerulus. Comprehensive single-cell labeling identified classes critical for behavioral responses to different CO_2 concentrations (Lin et al., 2013). However, one class highly selective for the V glomerulus could not be functionally studied because no GAL4 line was identified. Searching this neuron, we found the fourth hit (R86A05) was highly selective for this cell type (Figure 8E). Finally, we take an auditory interneuron (AMMC-AMMC PN1; Figures S6 and S2) and a presumptive visual interneuron of the anterior optic tubercle. The top ten hits for both neurons included numerous matching GAL4 lines; we display one example for each in Figure 8F. Although all of these lines label multiple neuronal classes, NBLAST enables very rapid identification of lines containing a neuronal population of interest that could be used for the construction of completely cell-type-specific lines by intersectional approaches (Luan et al., 2006).

DISCUSSION

Comprehensive mapping of neuronal types in the brain will depend on methods for unbiased classification of pools of thousands or millions of individual neurons. Comparison of neurons relies strongly on morphology and brain position, essential determinants of connectivity and function. A neuron similarity measure should (1) be accurate, generating biologically meaningful hits; (2) be computationally inexpensive; (3) enable interactive searches for data exploration; and (4) be generally applicable. NBLAST satisfies all these criteria.

First, NBLAST correctly distinguishes closely related types across a range of major neuron groups, achieving 97.6% accuracy for 35 types of olfactory PNs. Unsupervised neuron clustering based on NBLAST scores correctly organized neurons into known types. We did find that neuron sizes (especially when very small) can influence the algorithm: one future direction is to convert raw scores into an expectation (E) value that accounts for the size of a neuron and the database in direct analogy to the results of Karlin and Altschul (1990) for sequence alignments, although the problem appears more complicated for neurons.

Second, NBLAST searches are fast, with pairwise comparisons taking about 2 ms on a laptop. Furthermore, for defined datasets all-by-all scores can be pre-computed, enabling highly interactive analysis. With data volumes increasing, one effective approach to handle much larger numbers of neurons will be to compute sparse similarity matrices, storing the top *n* hits for a given neuron. Alternatively, queries could be computed only against the non-redundant set of neurons that collectively embody the structure of the brain (analogous to UniProt; Suzek et al., 2007). For the fly brain, this could not exceed 50,000 neurons (due to bilateral symmetry), and we expect the actual number to be ~5,000. Our clustering of all 16,129 FlyCircuit neurons identified 1,052 exemplars, providing a non-redundant dataset that we use for rapid searches.

Figure 6. NBLAST Classification of Sexually Dimorphic Neurons

(A) *fruitless* mAL neurons. Hierarchical clustering (HC) of hits cut into two groups (h = 1.25). Inset shows mAL query neuron (fru-M-500159). Leaves labeled with sex of neuron: female or male.

(B) Neurons from two dendrogram groups: male (cyan) and female (magenta).

(C) Analysis of male mAL neurons. Neuron segments for terminal arbors (ipsi- and contralateral) were isolated and NBLAST scores calculated. HC divided into three groups, I–III (h = 0.83). Arborization differences indicated by arrowheads. Group I (red) is further subdivided in two.

(D) *fruitless* P1 neurons. Plot of query neuron (fru-M-400046). Male enlarged brain region (MER) shown in red (anterior and posterior views). Volume rendering of pMP-e *fruitless* neuroblast clone containing P1 neurons. The distinctive primary neurite of pMP-e was traced.

(D′) HC of NBLAST hits for P1 trace divided into groups 1–10 (h = 0.92). Inset shows neurons colored by group. Leaves labeled by GAL4 driver used to obtain neuron, colored cyan (male) and magenta (female). Below dendrogram, neuron plots of each group; MER shown in gray for groups 9 and 10.

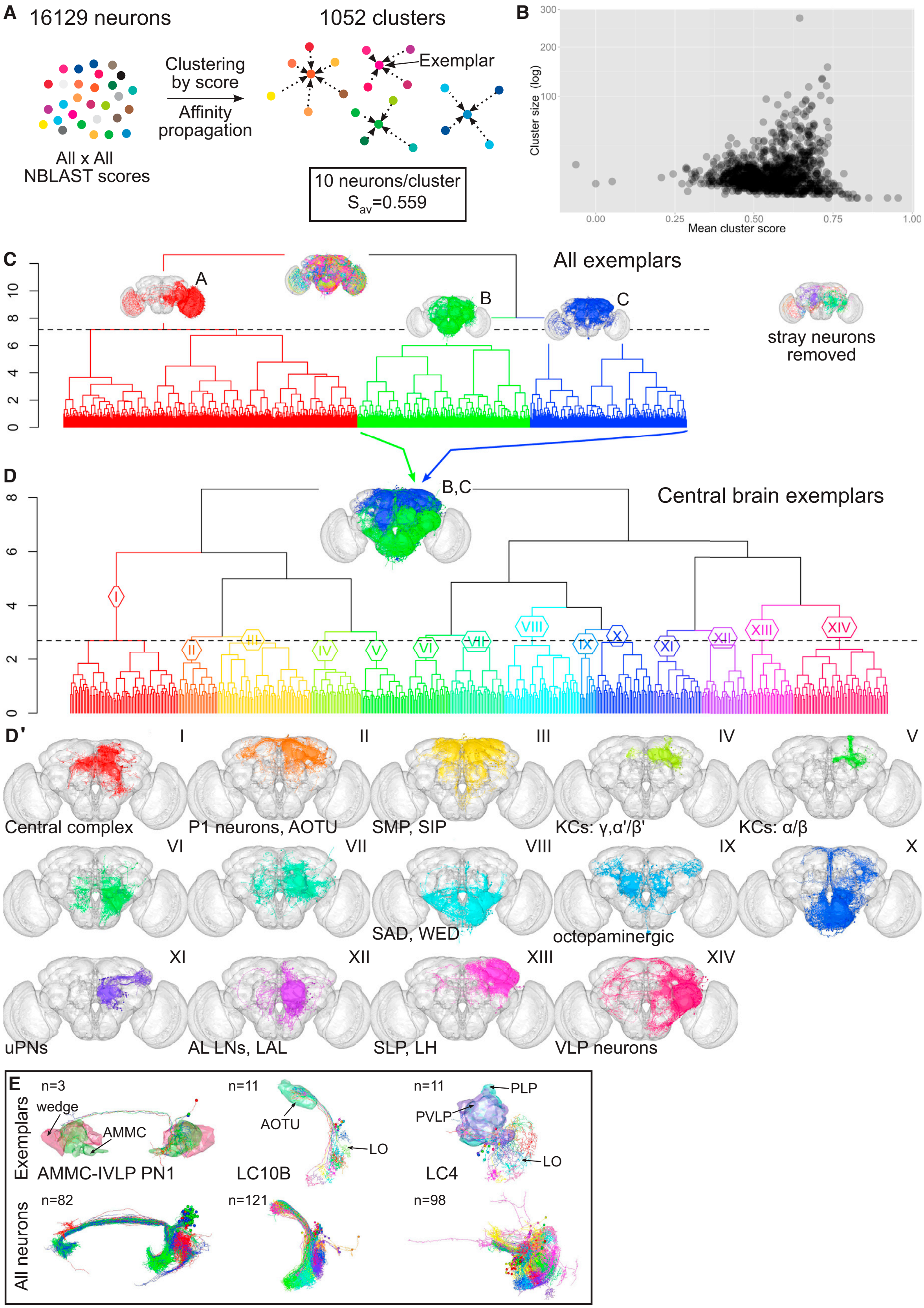

(legend on next page)

Third, NBLAST enables multiple types of analysis. Searches can use neuron fragments or tracings from complex image data as queries and databases of GAL4 lines as targets. Closely related neuronal types can be distinguished by clustering of only their terminal arbors without considering common features such as axon tracts.

Finally, one important question is the generality of our approach. This largely reduces to the relationship between length scales of neurons being examined and their absolute spatial stereotypy. Our method implicitly assumes spatial co-localization of related neurons; this is enforced by the use of image registration. Our strategy should be appropriate for any situation in which neuronal organization is highly stereotyped at the length scale of the neurons themselves. There is already strong evidence that this is true across large parts of the brain for simple vertebrate models like the larval zebrafish; indeed, we show that our NBLAST method can be applied directly to olfactory projectome data (Miyasaka et al., 2014). Mouse gene expression (Lein et al., 2007) and long-range connectivity also show global spatial stereotypy, as evidenced by recent atlas studies combining sparse labeling and image registration (Zingg et al., 2014; Susaki et al., 2014; Oh et al., 2014). Our method could be adapted for querying and hierarchical organization of these datasets by calculating an appropriate scoring matrix.

However, there are situations in which global brain registration is not appropriate. For example, the vertebrate retina has both laminar and tangential organization. Sümbül et al. (2014) recently introduced a registration strategy that showed that lamination of retinal ganglion cells is spatially stereotyped to the nearest micron. However, retinal interneurons and ganglion cells are organized in mosaics tangential to the retinal surface; global registration is not appropriate in this plane. The situation is similar for parallel columns of the outer *Drosophila* optic lobe. Possible approaches include local re-registration, mapping neurons onto a single canonical column, or amassing sufficient data so that neurons from neighboring columns/mosaics tile the brain, enabling identification of related groups by clustering or graph theoretic approaches.

Cataloguing all neuron types in the brain will rely not only on effective measures of neuronal similarity, but also on methods for automated classification of neurons into functionally relevant types. This is a challenging problem: it may be necessary to combine morphological approaches with data such as connectivity patterns, single-neuron gene expression patterns, or physiological properties to provide unambiguous automated classification (reviewed by Armañanzas and Ascoli, 2015). We have shown that NBLAST scores define a highly effective similarity metric that can be combined with hierarchical clustering and a specific dendrogram cut height to define a very wide range of neuronal classes. This approach enables very rapid exploratory analysis of new cell types even without expert neuroanatomical knowledge. Indeed, for *Drosophila* neurons it seems that NBLAST clustering is sufficient to define cell type.

Sümbül et al. (2014) recently explored the issue of defining the optimal dendrogram cut height for morphological clustering of 363 mouse retinal ganglion cells, establishing a reliable approach for these specific neurons. Nevertheless, our experience from the 16,129 neuron validation set is that differences in similarity levels within classically defined neuronal types preclude the existence of a universal value for dendrogram cut height. Some of this range (0.7 to 2 in this study) is probably due to differences in definitions: classic neuronal types may in some cases require splitting for consistency—we see evidence for this in the KC and visual PN datasets. More sophisticated statistical criteria may enable automated classification, especially when combined with measurements of, e.g., physiological or gene expression data (Armañanzas and Ascoli, 2015). However, all approaches to defining cluster numbers (i.e., statistically based cell types) depend on biological priors that must be acknowledged. Nevertheless, NBLAST's speed and sensitivity and the size of this validation dataset represent a significant step toward fully automated classification.

Finally, we note that NBLAST can identify genetic driver lines labeling a given query neuron. The pre-computed NBLAST result matrix that we provide for the GMR GAL4 collection (Jenett et al., 2012) will be of immediate utility to *Drosophila* colleagues planning experimental studies of particular cell classes. Thus, NBLAST can provide a vital link between studies of anatomical logic and neural circuit function.

EXPERIMENTAL PROCEDURES

Image Preprocessing

flycircuit.tw supplied 16,226 raw confocal stacks, which we converted to NRRD format with Fiji/ImageJ (http://fiji.sc/). We successfully preprocessed 16,204/16,226 total images, i.e., a 0.14% failure rate.

To make a template brain, we first averaged 17 female and 9 male brains to construct sex-specific templates using the CMTK (http://www.nitrc.org/projects/cmtk) avg_adm tool. We then averaged these two templates to generate an intersex template (FCWB) used for all subsequent analysis. We used CMTK to register images against this template first with a linear (9 degrees of freedom) and then a non-rigid registration (Rohlfing and Maurer, 2003; Jefferis et al., 2007). All registrations were checked by visual comparison

Figure 7. Organizing Huge Neuron Datasets

(A) Affinity propagation generated 1,052 clusters. The mean within-cluster similarity score was 0.559; mean cluster size = 10. Exemplars were identified for each cluster.

(B) Mean cluster score versus cluster size.

(C) Hierarchical clustering (HC) of 1,052 exemplars divided into three groups (A–C). Group A corresponds mostly to optic lobe and VPN neurons; B and C to central brain neurons. Insets show neurons from these groups. Right inset shows stray neurons removed (n = 11).

(D) HC of central brain exemplars (groups B and C, inset) cut into 14 groups; h = 2.7.

(D′) Neuron plots from dendrogram groups in (D). Main neuron types/innervated neuropils are noted.

(E) Affinity propagation of defined neuron types. Exemplar neurons (top row) or all neurons (bottom row) for auditory AMMC-IVLP PN1 neurons (compare Figure S6D) and VPN types LC10B (compare Figure 5B) and LC4 (compare Figure S5B). Numbers of exemplars and neurons indicated in top left corner. AMMC (antennal mechanosensory and motor center) in green; wedge in magenta; AOTU (anterior optic tubercle) in green; LO, lobula; PVLP (posterior ventrolateral protocerebrum) in purple; PLP (posterior lateral protocerebrum) in cyan.

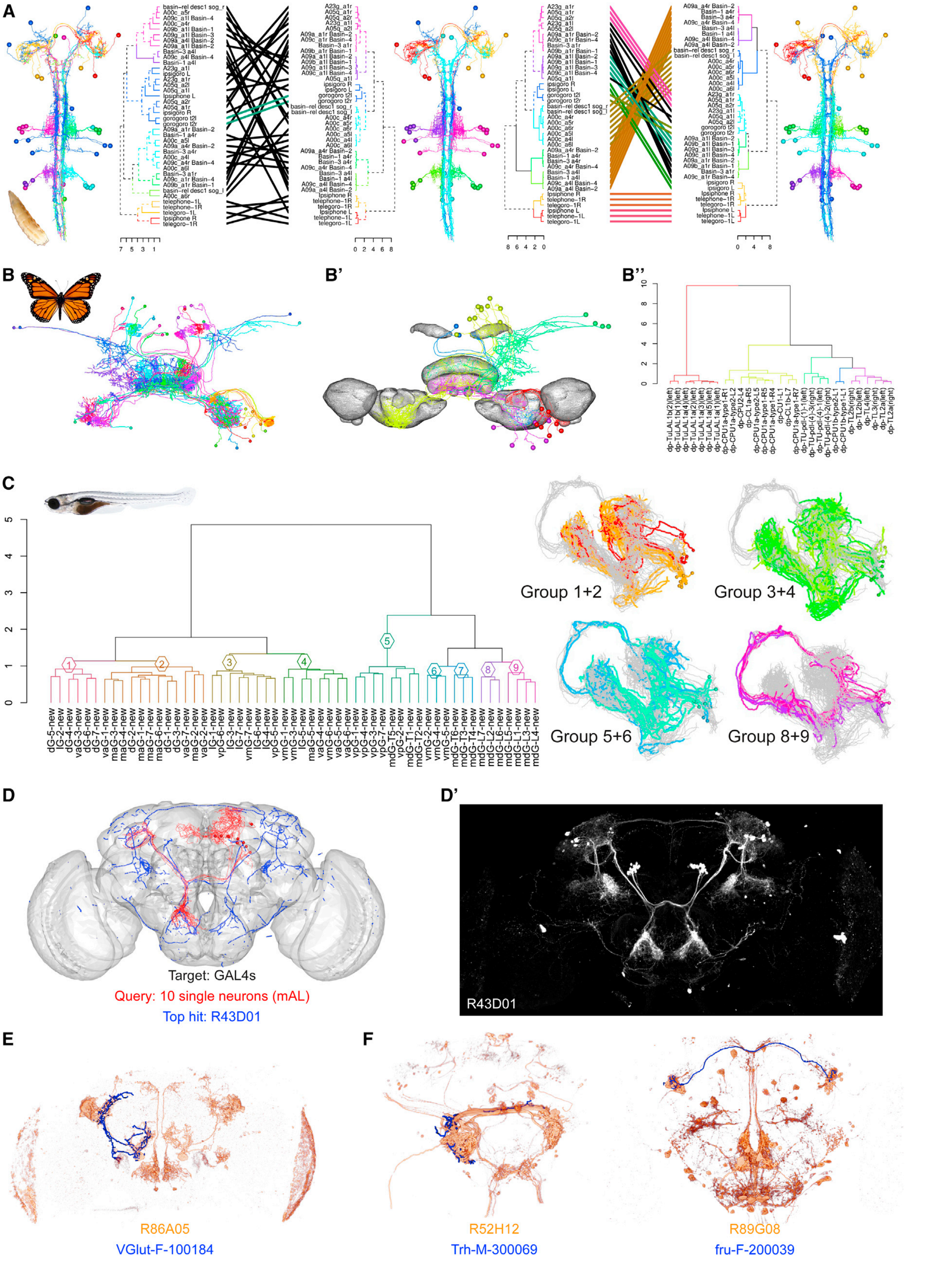

(legend on next page)

with the template in Amira (academic version, Zuse Institute). Poor registrations (10%) were re-initialized using affine registration based on a Global Hough Transform (Ballard, 1981; Khoshelham, 2007) calculated with an Amira extension module available from 1000shapes GmbH, or Amira's surface-based registration module, resulting in 16,129/16,204 successfully registered images (0.46% failure rate).

Chiang et al. (2011) included a segmented image for each neuron in their raw confocal dataset. We skeletonized this image using the Fiji plugin "Skeletonize (2D/3D)" (Doube et al., 2010) and then calculated a vector cloud representation for each skeleton (Masse et al., 2012) in R. Neurons on the right side of the brain were flipped to the left by applying a mirroring and flipping registration as described in Manton et al. (2014). We also calculated an overlap score for each neuron with the neuropil domains defined by Ito et al. (2014). See Supplemental Experimental Procedures for further details.

Neuron Search

Our reference implementation is the nblast function in the R package nat.nblast. Fast nearest-neighbor search depends on the nabo C++ library (Elseberg et al., 2012). The scoring matrix that we used for FlyCircuit neurons was constructed by taking 150 DL2 PNs, defining a neuron type at the finest level, and calculating the joint histogram of distance and absolute dot product for the 150 × 149 combinations of neurons, resulting in 1.4×10^7 measurement pairs; the number of counts in the histogram was then normalized (dividing by 1.4×10^7) to give a probability density, p_{match}. We then carried out a similar procedure for 5,000 random pairs of neurons sampled from the FlyCircuit dataset to give p_{rand}. Finally, the scoring matrix was calculated as $\log_2(p_{match} + \epsilon / p_{rand} + \epsilon)$, where $\varepsilon = 10^{-6}$ (a pseudocount to avoid infinite values).

Clustering

We used two methods for morphological clustering. For data subsets, we used hierarchical clustering with Ward's method (R function hclust). Dendrograms were cut at a height selected for each class (range 0.7–2), shown by a dashed line. By default, R plots the square of the Euclidean distance as the y axis, but we use the unsquared distance.

We used affinity propagation to cluster the whole dataset (Frey and Dueck, 2007) implemented in R package apcluster (Bodenhofer et al., 2011). This iterative method finds exemplars (representative members of each cluster) and does not require a priori input on the final number of clusters. An input preference parameter (p) can be set to control the final number of clusters. We used $p = 0$, since this is the value where, on average, matched segments are equally likely to have come from matching and non-matching neurons. Empirically, this produced clusters that mostly grouped neurons of the same type according to biological expert opinion.

Computer Code and Data

There is a dedicated website at http://jefferislab.org/si/nblast. This provides online NBLAST search tools, a web version of the affinity propagation clustering, video demos, and links to all computer code and data used to generate the figures in this paper, along with the open source libraries we have written and a help forum. See Supplemental Information for details.

SUPPLEMENTAL INFORMATION

Supplemental Information includes Supplemental Experimental Procedures, six figures, two tables, and three movies and can be found with this article online at http://dx.doi.org/10.1016/j.neuron.2016.06.012.

A video abstract is available at http://dx.doi.org/10.1016/j.neuron.2016.06.012#mmc6.

AUTHOR CONTRIBUTIONS

G.S.X.E.J. designed and implemented the NBLAST algorithm, supervised the study, and carried out initial data processing. M.C. and G.S.X.E.J. curated data, carried out validation studies of the NBLAST algorithm, and analyzed and visualized neuroanatomical data. J.D.M. refined NBLAST software, contributed to data visualization and analysis, and developed online search tools with G.S.X.E.J. A.D.O. constructed the template brain and contributed to initial image registration. S.P. implemented the Global Hough Transform registration pipeline and manual review system and investigated alternative search strategies. M.C. prepared figures with G.S.X.E.J. and J.D.M. M.C. and G.S.X.E.J. wrote the paper with J.D.M., incorporating feedback from A.D.O. and S.P.

ACKNOWLEDGMENTS

We first acknowledge A.S. Chiang and the flycircuit.tw team for generously providing the raw image data associated with Chiang et al. (2011). Images from FlyCircuit were obtained from the NCHC (National Center for High-Performance Computing) and NTHU (National Tsing Hua University). We thank S. Heinze for sharing monarch butterfly neuron tracings and useful discussions. We thank A. Cardona and M. Zlatic for larval *Drosophila* EM skeleton data. We thank N. Miyasaka, Y. Yoshihara, I. Arganda-Carreras, U. Sümbül, and S. Seung for sharing zebrafish mitral cell reconstructions. We thank A. Cardona, H. Cuntz, and members of the G.S.X.E.J. lab for comments on the manuscript; J. Grimmett and T. Darling for their vital assistance with the LMB compute cluster; and T. Rohlfing for discussions about image registration. We thank the Virtual Fly Brain project for help linking and incorporating some of the results of this study at http://www.virtualflybrain.org/. This study made use of the Computational Morphometry Toolkit, supported by the National Institute of Biomedical Imaging and Bioengineering. This work was supported by the Medical Research Council [MRC file reference U105188491] and European Research Council Starting and Consolidator Grants to G.S.X.E.J., who is an EMBO Young Investigator.

Received: April 2, 2016
Revised: May 13, 2016
Accepted: June 3, 2016
Published: June 30, 2016

REFERENCES

Altschul, S.F., Gish, W., Miller, W., Myers, E.W., and Lipman, D.J. (1990). Basic local alignment search tool. J. Mol. Biol. *215*, 403–410.

Figure 8. NBLAST extensions

(A) Larval *Drosophila* EM tracings. Tanglegrams match two different dendrograms; neurons are plotted by dendrogram group. In the middle, the original neurons; on the left, clustering based on synaptic connectivity network; on the right, NBLAST clustering after pruning first- and second-order terminal branches from neurons. Left tanglegram compares network clustering to NBLAST clustering; right tanglegram compares NBLAST clustering of original and pruned neurons.
(B) Monarch butterfly central complex neurons.
(B′) Neurons after mirroring and clustering colored by dendrogram group. Some brain neuropils are shown in gray.
(B′′) Hierarchical clustering after mirroring neurons.
(C) Zebrafish mitral neurons. Left: hierarchical clustering after mirroring neurons. Right: four examples of pairwise comparisons of neuron groups, colored by dendrogram group.
(D) Vector cloud representation of R43D01 expression pattern (blue) and query FlyCircuit mAL neurons (red).
(D′) R43D01 expression pattern.
(E) Candidate V-glomerulus-selective GAL4 expression pattern with query neuron (VGlut-F-100184).
(F) Identified GAL4 expression patterns found with AMMC-AMMC PN1 auditory neuron (Trh-M-300069) and AOTUv2 lineage neuron (fru-F-200039).

Armañanzas, R., and Ascoli, G.A. (2015). Towards the automatic classification of neurons. Trends Neurosci. *38*, 307–318.

Ascoli, G.A., Alonso-Nanclares, L., Anderson, S.A., Barrionuevo, G., Benavides-Piccione, R., Burkhalter, A., Buzsáki, G., Cauli, B., Defelipe, J., Fairén, A., et al.; Petilla Interneuron Nomenclature Group (2008). Petilla terminology: nomenclature of features of GABAergic interneurons of the cerebral cortex. Nat. Rev. Neurosci. *9*, 557–568.

Aso, Y., Grübel, K., Busch, S., Friedrich, A.B., Siwanowicz, I., and Tanimoto, H. (2009). The mushroom body of adult Drosophila characterized by GAL4 drivers. J. Neurogenet. *23*, 156–172.

Aso, Y., Hattori, D., Yu, Y., Johnston, R.M., Iyer, N.A., Ngo, T.T., Dionne, H., Abbott, L.F., Axel, R., Tanimoto, H., and Rubin, G.M. (2014). The neuronal architecture of the mushroom body provides a logic for associative learning. eLife *3*, e04577.

Badea, T.C., and Nathans, J. (2004). Quantitative analysis of neuronal morphologies in the mouse retina visualized by using a genetically directed reporter. J. Comp. Neurol. *480*, 331–351.

Ballard, D.H. (1981). Generalizing the Hough transform to detect arbitrary shapes. Pattern Recognit. *13*, 111–122.

Basu, S., Condron, B., and Acton, S.T. (2011). Path2Path: hierarchical path-based analysis for neuron matching. In Biomedical Imaging: From Nano to Macro, 2011 IEEE International Symposium on Biomedical Imaging (IEEE), pp. 996–999.

Bodenhofer, U., Kothmeier, A., and Hochreiter, S. (2011). APCluster: an R package for affinity propagation clustering. Bioinformatics *27*, 2463–2464.

Bota, M., and Swanson, L.W. (2007). The neuron classification problem. Brain Res. Brain Res. Rev. *56*, 79–88.

Cachero, S., Ostrovsky, A.D., Yu, J.Y., Dickson, B.J., and Jefferis, G.S.X.E. (2010). Sexual dimorphism in the fly brain. Curr. Biol. *20*, 1589–1601.

Cajal, S.R. (1911). Histologie du Système Nerveux de l'Homme et des Vertébrés (Maloine).

Cardona, A., Saalfeld, S., Arganda, I., Pereanu, W., Schindelin, J., and Hartenstein, V. (2010). Identifying neuronal lineages of Drosophila by sequence analysis of axon tracts. J. Neurosci. *30*, 7538–7553.

Chiang, A.S., Lin, C.Y., Chuang, C.C., Chang, H.M., Hsieh, C.H., Yeh, C.W., Shih, C.T., Wu, J.J., Wang, G.T., Chen, Y.C., et al. (2011). Three-dimensional reconstruction of brain-wide wiring networks in Drosophila at single-cell resolution. Curr. Biol. *21*, 1–11.

Doube, M., Kłosowski, M.M., Arganda-Carreras, I., Cordelières, F.P., Dougherty, R.P., Jackson, J.S., Schmid, B., Hutchinson, J.R., and Shefelbine, S.J. (2010). BoneJ: free and extensible bone image analysis in ImageJ. Bone *47*, 1076–1079.

El Jundi, B., Heinze, S., Lenschow, C., Kurylas, A., Rohlfing, T., and Homberg, U. (2010). The locust standard brain: a 3D standard of the central complex as a platform for neural network analysis. Front. Syst. Neurosci. *3*, 21.

Elseberg, J., Magnenat, S., Siegwart, R., and Nüchter, A. (2012). Comparison of nearest-neighbor-search strategies and implementations for efficient shape registration. Journal of Software Engineering for Robotics *3*, 2–12.

Fischbach, K.F., and Dittrich, A. (1989). The optic lobe of Drosophila melanogaster. I. A Golgi analysis of wild-type structure. Cell Tissue Res. *258*, 441–475.

Frey, B.J., and Dueck, D. (2007). Clustering by passing messages between data points. Science *315*, 972–976.

Ganglberger, F., Schulze, F., Tirian, L., Novikov, A., Dickson, B., Bühler, K., and Langs, G. (2014). Structure-based neuron retrieval across Drosophila brains. Neuroinformatics *12*, 423–434.

Heinze, S., Florman, J., Asokaraj, S., El Jundi, B., and Reppert, S.M. (2013). Anatomical basis of sun compass navigation II: the neuronal composition of the central complex of the monarch butterfly. J. Comp. Neurol. *521*, 267–298.

Ito, K., Shinomiya, K., Ito, M., Armstrong, J.D., Boyan, G., Hartenstein, V., Harzsch, S., Heisenberg, M., Homberg, U., Jenett, A., et al.; Insect Brain Name Working Group (2014). A systematic nomenclature for the insect brain. Neuron *81*, 755–765.

Jefferis, G.S.X.E., and Livet, J. (2012). Sparse and combinatorial neuron labelling. Curr. Opin. Neurobiol. *22*, 101–110.

Jefferis, G.S.X.E., and Manton, J.D. (2014). NeuroAnatomy Toolbox v1.5.2. Zenodo. http://dx.doi.org/10.5281/zenodo.10171.

Jefferis, G.S., Marin, E.C., Stocker, R.F., and Luo, L. (2001). Target neuron prespecification in the olfactory map of Drosophila. Nature *414*, 204–208.

Jefferis, G.S.X.E., Potter, C.J., Chan, A.M., Marin, E.C., Rohlfing, T., Maurer, C.R.J., Jr., and Luo, L. (2007). Comprehensive maps of Drosophila higher olfactory centers: spatially segregated fruit and pheromone representation. Cell *128*, 1187–1203.

Jenett, A., Rubin, G.M., Ngo, T.T., Shepherd, D., Murphy, C., Dionne, H., Pfeiffer, B.D., Cavallaro, A., Hall, D., Jeter, J., et al. (2012). A GAL4-driver line resource for Drosophila neurobiology. Cell Rep. *2*, 991–1001.

Kahsai, L., and Zars, T. (2011). Learning and memory in Drosophila: behavior, genetics, and neural systems. Int. Rev. Neurobiol. *99*, 139–167.

Kallman, B.R., Kim, H., and Scott, K. (2015). Excitation and inhibition onto central courtship neurons biases Drosophila mate choice. eLife *4*, e11188.

Kamikouchi, A., Shimada, T., and Ito, K. (2006). Comprehensive classification of the auditory sensory projections in the brain of the fruit fly Drosophila melanogaster. J. Comp. Neurol. *499*, 317–356.

Kamikouchi, A., Inagaki, H.K., Effertz, T., Hendrich, O., Fiala, A., Göpfert, M.C., and Ito, K. (2009). The neural basis of Drosophila gravity-sensing and hearing. Nature *458*, 165–171.

Karlin, S., and Altschul, S.F. (1990). Methods for assessing the statistical significance of molecular sequence features by using general scoring schemes. Proc. Natl. Acad. Sci. USA *87*, 2264–2268.

Kepecs, A., and Fishell, G. (2014). Interneuron cell types are fit to function. Nature *505*, 318–326.

Khoshelham, K. (2007). Extending generalized hough transform to detect 3D objects in laser range data. In ISPRS Workshop on Laser Scanning, ISPRS Proceedings (ISPRS), pp. 206–210.

Kimura, K., Ote, M., Tazawa, T., and Yamamoto, D. (2005). Fruitless specifies sexually dimorphic neural circuitry in the Drosophila brain. Nature *438*, 229–233.

Kimura, K., Hachiya, T., Koganezawa, M., Tazawa, T., and Yamamoto, D. (2008). Fruitless and doublesex coordinate to generate male-specific neurons that can initiate courtship. Neuron *59*, 759–769.

Koganezawa, M., Haba, D., Matsuo, T., and Yamamoto, D. (2010). The shaping of male courtship posture by lateralized gustatory inputs to male-specific interneurons. Curr. Biol. *20*, 1–8.

Kong, J.H., Fish, D.R., Rockhill, R.L., and Masland, R.H. (2005). Diversity of ganglion cells in the mouse retina: unsupervised morphological classification and its limits. J. Comp. Neurol. *489*, 293–310.

Lai, J.S.Y., Lo, S.J., Dickson, B.J., and Chiang, A.S. (2012). Auditory circuit in the Drosophila brain. Proc. Natl. Acad. Sci. USA *109*, 2607–2612.

Lee, T.C., Kashyap, R.L., and Chu, C.N. (1994). Building skeleton models via 3-D medial surface/axis thinning algorithms. CVGIP Graph. Models Image Process. *56*, 462–478.

Lee, T., Lee, A., and Luo, L. (1999). Development of the Drosophila mushroom bodies: sequential generation of three distinct types of neurons from a neuroblast. Development *126*, 4065–4076.

Lein, E.S., Hawrylycz, M.J., Ao, N., Ayres, M., Bensinger, A., Bernard, A., Boe, A.F., Boguski, M.S., Brockway, K.S., Byrnes, E.J., et al. (2007). Genome-wide atlas of gene expression in the adult mouse brain. Nature *445*, 168–176.

Lin, H.H., Lai, J.S.Y., Chin, A.L., Chen, Y.C., and Chiang, A.S. (2007). A map of olfactory representation in the Drosophila mushroom body. Cell *128*, 1205–1217.

Lin, C.Y., Chuang, C.C., Hua, T.E., Chen, C.C., Dickson, B.J., Greenspan, R.J., and Chiang, A.S. (2013). A comprehensive wiring diagram of the protocerebral

bridge for visual information processing in the Drosophila brain. Cell Rep. *3*, 1739–1753.

Luan, H., Peabody, N.C., Vinson, C.R., and White, B.H. (2006). Refined spatial manipulation of neuronal function by combinatorial restriction of transgene expression. Neuron *52*, 425–436.

Manton, J.D., Ostrovsky, A.D., Goetz, L., Costa, M., Rohlfing, T., and Jefferis, G.S.X.E. (2014). Combining genome-scale Drosophila 3D neuroanatomical data by bridging template brains. bioRxiv, Published online June 19, 2014. http://dx.doi.org/10.1101/006353.

Marin, E.C., Jefferis, G.S., Komiyama, T., Zhu, H., and Luo, L. (2002). Representation of the glomerular olfactory map in the Drosophila brain. Cell *109*, 243–255.

Masse, N.Y., Turner, G.C., and Jefferis, G.S.X.E. (2009). Olfactory information processing in Drosophila. Curr. Biol. *19*, R700–R713.

Masse, N.Y., Cachero, S., Ostrovsky, A.D., and Jefferis, G.S.X.E. (2012). A mutual information approach to automate identification of neuronal clusters in Drosophila brain images. Front. Neuroinform. *6*, 21.

Matsuo, E., Seki, H., Asai, T., Morimoto, T., Miyakawa, H., Ito, K., and Kamikouchi, A. (2016). Organization of projection neurons and local neurons of the primary auditory center in the fruit fly Drosophila melanogaster. J. Comp. Neurol. *524*, 1099–1164.

Mayerich, D., Bjornsson, C., Taylor, J., and Roysam, B. (2012). NetMets: software for quantifying and visualizing errors in biological network segmentation. BMC Bioinformatics *13* (*Suppl 8*), S7.

Migliore, M., and Shepherd, G.M. (2005). Opinion: an integrated approach to classifying neuronal phenotypes. Nat. Rev. Neurosci. *6*, 810–818.

Miyasaka, N., Arganda-Carreras, I., Wakisaka, N., Masuda, M., Sümbül, U., Seung, H.S., and Yoshihara, Y. (2014). Olfactory projectome in the zebrafish forebrain revealed by genetic single-neuron labelling. Nat. Commun. *5*, 3639.

Morante, J., and Desplan, C. (2008). The color-vision circuit in the medulla of Drosophila. Curr. Biol. *18*, 553–565.

Nelson, S.B., Sugino, K., and Hempel, C.M. (2006). The problem of neuronal cell types: a physiological genomics approach. Trends Neurosci. *29*, 339–345.

Oh, S.W., Harris, J.A., Ng, L., Winslow, B., Cain, N., Mihalas, S., Wang, Q., Lau, C., Kuan, L., Henry, A.M., et al. (2014). A mesoscale connectome of the mouse brain. Nature *508*, 207–214.

Ohyama, T., Schneider-Mizell, C.M., Fetter, R.D., Aleman, J.V., Franconville, R., Rivera-Alba, M., Mensh, B.D., Branson, K.M., Simpson, J.H., Truman, J.W., et al. (2015). A multilevel multimodal circuit enhances action selection in Drosophila. Nature *520*, 633–639.

Otsuna, H., and Ito, K. (2006). Systematic analysis of the visual projection neurons of Drosophila melanogaster. I. Lobula-specific pathways. J. Comp. Neurol. *497*, 928–958.

Parekh, R., and Ascoli, G.A. (2013). Neuronal morphology goes digital: a research hub for cellular and system neuroscience. Neuron *77*, 1017–1038.

Peng, H., Tang, J., Xiao, H., Bria, A., Zhou, J., Butler, V., Zhou, Z., Gonzalez-Bellido, P.T., Oh, S.W., Chen, J., et al. (2014). Virtual finger boosts three-dimensional imaging and microsurgery as well as terabyte volume image visualization and analysis. Nat. Commun. *5*, 4342.

Peng, H., Hawrylycz, M., Roskams, J., Hill, S., Spruston, N., Meijering, E., and Ascoli, G.A. (2015). BigNeuron: large-scale 3D neuron reconstruction from optical microscopy images. Neuron *87*, 252–256.

Rohlfing, T., and Maurer, C.R.J., Jr. (2003). Nonrigid image registration in shared-memory multiprocessor environments with application to brains, breasts, and bees. IEEE Trans. Inf. Technol. Biomed. *7*, 16–25.

Rowe, M.H., and Stone, J. (1977). Naming of neurones. Classification and naming of cat retinal ganglion cells. Brain Behav. Evol. *14*, 185–216.

Rybak, J., Kuß, A., Lamecker, H., Zachow, S., Hege, H.C., Lienhard, M., Singer, J., Neubert, K., and Menzel, R. (2010). The digital bee brain: integrating and managing neurons in a common 3D reference system. Front. Syst. Neurosci. *4*, http://dx.doi.org/10.3389/fnsys.2010.00030.

Schindelin, J., Arganda-Carreras, I., Frise, E., Kaynig, V., Longair, M., Pietzsch, T., Preibisch, S., Rueden, C., Saalfeld, S., Schmid, B., et al. (2012). Fiji: an open-source platform for biological-image analysis. Nat. Methods *9*, 676–682.

Sümbül, U., Song, S., McCulloch, K., Becker, M., Lin, B., Sanes, J.R., Masland, R.H., and Seung, H.S. (2014). A genetic and computational approach to structurally classify neuronal types. Nat. Commun. *5*, 3512.

Sunkin, S.M., Ng, L., Lau, C., Dolbeare, T., Gilbert, T.L., Thompson, C.L., Hawrylycz, M., and Dang, C. (2013). Allen Brain Atlas: an integrated spatio-temporal portal for exploring the central nervous system. Nucleic Acids Res. *41*, D996–D1008.

Susaki, E.A., Tainaka, K., Perrin, D., Kishino, F., Tawara, T., Watanabe, T.M., Yokoyama, C., Onoe, H., Eguchi, M., Yamaguchi, S., et al. (2014). Whole-brain imaging with single-cell resolution using chemical cocktails and computational analysis. Cell *157*, 726–739.

Suzek, B.E., Huang, H., McGarvey, P., Mazumder, R., and Wu, C.H. (2007). UniRef: comprehensive and non-redundant UniProt reference clusters. Bioinformatics *23*, 1282–1288.

Tanaka, N.K., Tanimoto, H., and Ito, K. (2008). Neuronal assemblies of the Drosophila mushroom body. J. Comp. Neurol. *508*, 711–755.

Tanaka, N.K., Endo, K., and Ito, K. (2012). Organization of antennal lobe-associated neurons in adult Drosophila melanogaster brain. J. Comp. Neurol. *520*, 4067–4130.

Wan, Y., Long, F., Qu, L., Xiao, H., Hawrylycz, M., Myers, E.W., and Peng, H. (2015). BlastNeuron for automated comparison, retrieval and clustering of 3D neuron morphologies. Neuroinformatics *13*, 487–499.

Wong, A.M., Wang, J.W., and Axel, R. (2002). Spatial representation of the glomerular map in the Drosophila protocerebrum. Cell *109*, 229–241.

Yorozu, S., Wong, A., Fischer, B.J., Dankert, H., Kernan, M.J., Kamikouchi, A., Ito, K., and Anderson, D.J. (2009). Distinct sensory representations of wind and near-field sound in the Drosophila brain. Nature *458*, 201–205.

Yu, H.H., Kao, C.F., He, Y., Ding, P., Kao, J.C., and Lee, T. (2010a). A complete developmental sequence of a Drosophila neuronal lineage as revealed by twin-spot MARCM. PLoS Biol. *8*, http://dx.doi.org/10.1371/journal.pbio.1000461.

Yu, J.Y., Kanai, M.I., Demir, E., Jefferis, G.S.X.E., and Dickson, B.J. (2010b). Cellular organization of the neural circuit that drives Drosophila courtship behavior. Curr. Biol. *20*, 1602–1614.

Zhu, S., Chiang, A.S., and Lee, T. (2003). Development of the Drosophila mushroom bodies: elaboration, remodeling and spatial organization of dendrites in the calyx. Development *130*, 2603–2610.

Zingg, B., Hintiryan, H., Gou, L., Song, M.Y., Bay, M., Bienkowski, M.S., Foster, N.N., Yamashita, S., Bowman, I., Toga, A.W., and Dong, H.W. (2014). Neural networks of the mouse neocortex. Cell *156*, 1096–1111.

Neuron, Volume *91*

Supplemental Information

NBLAST: Rapid, Sensitive Comparison of Neuronal Structure and Construction of Neuron Family Databases

Marta Costa, James D. Manton, Aaron D. Ostrovsky, Steffen Prohaska, and Gregory S.X.E. Jefferis

Supplemental Information

Supplemental Experimental Procedures

Image Preprocessing

flycircuit.tw supplied 16,226 raw confocal stacks in Zeiss LSM format on a single 2 TB hard drive in April 2011. Each stack was uncompressed, then read into Fiji/ImageJ (`http://fiji.sc/Fiji`) where channels were split and resaved as individual NRRD (`http://teem.sourceforge.net/nrrd`) files. Where calibration information was missing in the LSM metadata, we used a voxel size of (0.318427, 0.318427, 1.00935) microns as recommended by the FlyCircuit team. There were two issues to solve before images could be used for registration: 1) identifying which image channel contained the anti-Dlg (discs large 1) counterstaining 2) determining whether the brains had been imaged from anterior to posterior, or the reverse. The first issue was solved by exporting metadata associated with each LSM using the LOCI bioformats (`http://loci.wisc.edu/software/bio-formats`) plugin and developing heuristics to automate the identification of the channel sequence; for a minority of images metadata was missing and channel order was determined manually. Slice order could not be determined automatically from the image metadata. We therefore made maximum intensity Z projections (using the unu tool, `http://teem.sourceforge.net/unrrdu`) for the labeled neuron channel for each stack. Each projection was then compared with the matching thumbnail available from the flycircuit.tw website. The correlation score between the projection and thumbnail images was calculated both with and without a mirror flip across the YZ plane; a large correlation score for only one orientation was used as evidence for a given slice ordering. A small number of ambiguous results were verified manually. We successfully preprocessed 16,204/16,226 total images i.e. a 0.14 % failure rate. 12 failures were due to mismatches that could not be resolved between the segmented neuron present in the LSM file and the thumbnail image on the flycircuit.tw website; the remaining 10 failures were due to physical offsets between the brain and GFP channels or corrupt image data.

Template Brain

The template brain (FCWB) was constructed by screening for whole brains within the FlyCircuit dataset and manually selecting a pool of stacks that appeared of good quality. Separate average female and average male template brains were constructed from 17 and 9 brains, respectively using the CMTK (`http://www.nitrc.org/projects/cmtk`) `avg_adm` tool which takes a single brain as a seed. After five iterations the resultant average male and average female brains were placed in an affine symmetric position within their image stacks so that a simple horizontal (x-axis) flip of either template brain resulted in an almost perfect overlap of left and right hemispheres. Finally the two sex-specific template brains were then averaged (with equal weight) to make an intersex template brain using `avg_adm`. Since this template was designed to provide an optimized registration target for the FlyCircuit dataset, no correction was made for the disparity between the XY and Z voxel dimensions common to all images in the dataset. The scripts used for the construction of the template are available at `https://github.com/jefferislab/MakeAverageBrain`.

Image Registration

Image registration of Dlg neuropil staining used a fully automatic intensity-based (landmark free) 3D image registration implemented in the CMTK toolkit (Rohlfing and Maurer, 2003; Jefferis et al., 2007). An initial linear registration with 9 degrees of freedom (translation, rotation and scaling of each axis) was followed by a non-rigid registration that allows different brain regions to move independently, subject to a smoothness penalty (Rueckert et al., 1999). It is our experience that obtaining a satisfactory initial linear registration is crucial. All registrations were therefore manually checked by comparing the reformatted brain with the template in Amira (academic version, Zuse Institute, Berlin), using ResultViewer `https://bitbucket.org/jefferis/resultviewer`. This

identified about 10 % of brains with poor initial registrations. For these images a new affine registration was calculated using a Global Hough Transform (Ballard, 1981; Khoshelham, 2007) with an Amira extension module available from 1000shapes GmbH; the result of this affine transform was again manually inspected. In the minority of cases where this approach failed, a surface based alignment was calculated in Amira after manually aligning the two brains. Following satisfactory initial affine registration, a non-rigid registration was calculated. Finally each registration was checked manually in Amira against the template brain. This sequential procedure was resulted in successfully registration of 16,129/16,204 preprocessed images, giving a registration failure rate of 0.46 %.

Image Postprocessing

The confocal stack for each neuron available at http://flycircuit.tw includes an 8 bit image containing a single (semiautomatically) segmented neuron prepared by Chiang et al. (2011). This image was downsampled by a factor of 2 in x and y, binarized with a threshold of 1 and skeletonized using the Fiji plugin 'Skeletonize (2D/3D)' (Doube et al., 2010). Dot properties for each skeleton Masse et al. (2012), were extracted using the `dotprops` function of our new nat package for R. This converted each skeleton into segments, described by its location and tangent vector. Neurons on the right side of the brain were flipped to the left by applying a mirroring and a flipping registration as described in Manton et al. (2014). The decision to flip a neuron depended on earlier assignment of each neuron to a brain hemisphere using a combination of automated and manual approaches. Neurons whose cell bodies were more than 20 µm away from the mid-sagittal YZ plane were automatically defined as belonging to the left or right hemisphere. Neurons with cell bodies inside this 40 µm central corridor were manually assigned to a hemisphere, based on cell body position (right or left side), path taken by the primary neurite, location and length of first branching neurite. For example, neurons that had a cell body on the midline with significant innervation from the first branching neurite near the cell body on the left hemisphere, with the rest of the arborization on the right, were assigned to the left side and not flipped.

The cell body positions used were based on those published on the http://flycircuit.tw website for each neuron; these positions are in the space of the FlyCircuit female and male template brains (typical_brain_female and typical_brain_male). In order to transform them into the new FCWB template that we constructed, affine bridging registrations were constructed from the typical_brain_female and typical_brain_male brains to FCWB and the cell body positions were then transformed to this new space. Since these cell body positions depend on two affine registrations (one conducted by Chiang et al. (2011) to register each sample brain onto either their typical_brain_female or typical_brain_male templates and a second carried out by us to map those template brains onto our FCWB template) these positions are likely accurate only to ±5 microns in each axis.

We also calculated an overlap score for each neuron with the neuropil regions defined by Ito et al. (2014). We converted the JFRC2 space segmented neuropil volume available from virtualflybrain.org to the FCWB template space used in this study using the approach of Manton et al. (2014).

NBLAST Design

The algorithm design process was primarily motivated by a requirement for rapid and sensitive searches of neuron databases. It was necessary to consider both the data structure and the similarity algorithm jointly in the face of the design requirements. Our ideas in this area have been under independent development since 2007, but our efforts were strongly motivated by a very large single neuron dataset (Chiang et al., 2011) which offered the possibility of validating candidate algorithms at scale. We begin by summarizing published algorithms and data structures that represent different strategies that we were aware of as our own began to mature.

Mayerich et al. (2012) applied general graph similarity metrics (reviewed by Conte et al., 2004) to compare neurons represented as fully-connected graphs with a ground truth reconstruction; however this approach emphasizes the details of branching structure over positional information. Basu et al. (2011) decomposed neuronal trees into a family of unbranched paths proposing a geometric measure of similarity that could include positional informa-

tion, however the approach was not further developed or validated. Further simplifying the neuronal representation, Cardona et al. (2010) decomposed single unbranched neurites into sequences of vectors and used dynamic programming to find an optimal 3D alignment. Critically, they validated this approach on a database of a few hundred traced structures, achieving very high classification accuracy. However as noted in the main Introduction, this algorithm treats each unbranched neuronal segment as a separate alignment problem, so there is no natural way to handle trees with many such segments.

Recently the approach of Wan et al. (2015) combining graph matching with 3D positional information represents a natural combination of these different methods. However it seems restricted to a specific set of problems since 1) computation times are of the order minutes per neuron pair 2) it is restricted to fully traced neurons 3) it does not presently handle partial matches 4) it is not designed to use absolute positional information of neurons in a brain or brain structure.

One other approach developed in parallel to our own algorithm was presented by Ganglberger et al. (2014) who compared it to our earlier work (Masse et al., 2012). This uses a flexible data representation in which co-registered volumetric image data are smoothed and a vector field is calculated representing the local direction of tubular objects in the image. This approach has some advantages of the method that we will now describe but the use of large image volumes severely limits the throughput that can be achieved (the authors reported loading data for 1000 brains into memory over 15 mins). Furthermore the relatively small test dataset available to those authors did not allow them to make a strong case for the sensitivity of their algorithm.

Our first application would be data acquired in *Drosophila*, where previous studies using image registration have shown a high degree of spatial stereotypy (standard deviation of landmarks ~2.5 µm in each axis for a brain of 600 µm in its longest axis, Jefferis et al., 2007). Therefore one key design decision was to use co-registered data rather than calculating similarity using features of the neuron that are independent of absolute spatial location.

On the algorithm side, the key initial design decision was whether to develop a direct pairwise comparison algorithm or to use a form of dimensional reduction to map neuronal structure into a lower-dimensional space. The major advantage of the latter approach is that the similarity between neurons can be computed directly and almost instantaneously in the low dimensional space. However, the construction of a suitable embedding function either requires existing knowledge of neuronal similarity (likely supplied by experts in the form of large amounts of training data), huge amounts of unlabeled data that enable direct learning of features (e.g. Le et al., 2012), or a strategy based on successful extraction of key image features.

A number of considerations made us favor the approach of direct pairwise comparison. First, we suspected that it would be possible to make a more sensitive algorithm by working with the original data. Second, the amounts of image data available did not seem large enough to avoid a requirement for extensive labeled training data. Third, we reasoned that our own intuitions about neuronal similarity could be better expressed in the original physical space of the neuron than in a low dimensional embedding. Our own exploratory analysis in which we summarized each neuron in different ways as feature vectors of the same dimension and used a comparison function in the feature space (SP, GSXEJ unpublished observations) confirmed that constructing a sensitive metric of this sort is challenging.

The selection of a pairwise similarity metric meant that we had to give particularly careful consideration to performance issues in the design phase. We set two practical performance targets: 1) being able to carry out searches of a single neuron against a database of 10,000 neurons in less than a minute on a simple desktop or laptop computer. 2) Being able to complete all-against-all searches for 10,000 neurons (10^8 comparisons) in $<$ 1 day on a powerful desktop computer. These targets meant that each elementary comparison operation should take around 5 ms or less. Image pre-processing carried out once per neuron would therefore be a good investment if it reduced the time taken for each pairwise comparison. These considerations prompted us to generate a spatially registered, compact representation of each neuron as a separate pre-processing step, rather than develop an algorithm that simultaneously solved both the spatial alignment and similarity problem.

NBLAST Scoring

As described in the main results section, we defined NBLAST raw scores as the sum of segment pair scores:

$$S(\text{query}, \text{target}) = \sum_{i=1}^{n} f(d_i, |\vec{u}_i \cdot \vec{v}_i|). \tag{3}$$

We initially experimented with a function based on expert intuition:

$$f = \sqrt{|\vec{u}_i \cdot \vec{v}_i| \exp\left(-d_i^2/2\sigma^2\right)}. \tag{4}$$

This includes a negative exponential function of distance (related to the normal distribution), with a free parameter σ based on our previous estimates of the variability in position within the fly brain of landmarks after registration (Jefferis et al., 2007; Yu et al., 2010) set to 3 µm. Although this provided a useful starting point, we were unhappy with a scoring system that required parameters to be specified rather than derived empirically from data. This then motivated us to investigate the statistical scoring approach based on log probability ratios. This single parameter approach may still be helpful in some situations where insufficient neurons are available in order to define a statistical scoring matrix (we used 150 similar neurons and 5,000 random pairs). It can also enable a bootstrapping approach for new datasets in order to help identify similar pairs of neurons that can then be used to define a full scoring matrix.

Neuron Tracing

Neuron tracing for some sample queries was carried out in Amira (commercial version, FEI Visualization Sciences Group, Merignac, France) using the hxskeletonize plugin (Evers et al., 2005) or in Vaa3D (Peng et al., 2014) on registered image data. Traces were loaded into R using the `nat` package. When necessary, they were transformed into the space of the FCWB template brain (Manton et al., 2014).

Olfactory Projection Neuron Analysis

We started by manually classifying the 400 uPNs in the FlyCircuit dataset by glomerulus, neuroblast lineage, and axon tract, using the original image stacks. The definition of the manual gold standard annotations was an iterative process that took several days. The first round accuracy was about 95 %. Numerous discrepancies were revealed by subsequent NBLAST analysis and difficult cases were resolved by discussion between two expert annotators before finalizing assignments. We excluded 3 neurons for which no conclusion could be reached. We found a very large number of DL2 uPNs, (145 DL2d and 37 DL2v), in a total of 397 neurons. Nevertheless, our final set of uPNs broadly represents the total variability of described classes and contains neurons innervating 35 out of 56 different glomeruli (Tanaka et al., 2012), examples of the three main lineage clones (adPNs, lPNs and vPNs) in addition to one bilateral uPN, and neurons that follow each of the three main tracts (medial, mediolateral and lateral antennal lobe tracts). For subsequent analysis, we removed 3 neurons for which registration failed.

We restricted the analysis in Figure S4A to types with at least two examples in the dataset and to unique pairs (if PN A and PN B were top hits for each other, we only counted them once) (n=327). There were only 8 cases in which the top hit did not match the query neuron's class. Of these, four had matches to a uPN innervating a neighboring glomerulus with identical axon projections (DL2d vs DL2v, VM5d vs VM5v) that are challenging even for experts to distinguish. There were a further four matches to multi-glomerular PNs that innervated the same glomerulus as well as some others.

There were three mis-clustered neurons in the unsupervised hierarchical clustering (Figure 4E). For one case there was substantial mis-registration of the antennal lobe overlooked by our image registration quality control; in the other 2 cases, neurons of the same type were split into two adjacent clusters.

Visual Projection Neuron Analysis

We started with the 1,052 exemplars found by affinity propagation clustering of NBLAST scores (Figure 7). We then clustered those exemplars using hierarchical clustering and found that extrinsic and intrinsic optic lobe neurons together formed a distinct "optic lobe" group within this (Figure 7C). We then collected all neurons associated with those "optic lobe" exemplars and calculated the overlap of neurons with each of the standard neuropils defined by Ito et al. (2014) (see Experimental Procedures and Manton et al., 2014 for technical details). This then allowed us to separate neurons by innervation pattern into 3 groups: 1) ipsilateral optic lobe neuropils only (see Figure S5D for intrinsic optic lobe neurons), 2) ipsilateral and central brain neuropils (unilateral VPNs, uVPNs) or 3) both optic lobes and central brain neuropils (bilateral VPNs). This selection procedure resulted in a set of 1,793 uVPNs, 72 bilateral VPNs and 2,892 intrinsic optic lobe neurons.

Hierarchical clustering of the unilateral VPNs (uVPNs) resulted in a dendrogram, which we divided into 21 groups (I-XXI), in order to isolate one or a few cell types by group based both directly on morphological stereotypy and on previous literature (Otsuna and Ito, 2006) (Figure 5A–A' and Figure S5A–A').

To isolate uVPNs that overlap the lobula, anterior optic tubercle (AOTU) and posterior ventrolateral protocerebrum (PVLP), we took neuron skeletons from groups I–III uVPN and isolated the axon arbors innervating the two latter neuropils (Figure 5B). A new clustering based on the scores of these partial skeletons, allowed us to identify 7 different groups (1-7). A clear distinction between neurons that innervated the PVLP (groups 1, 2) and those that extensively innervated the AOTU (groups 3–6) was evident.

To isolate uVPNs that overlap the lobula, PVLP- and posterior lateral protocerebrum (PLP) we performed a similar analysis with uVPN groups that had dendritic innervation restricted to the lobula and axons projecting to the PVLP and PLP (groups IV, VI, VII and XI) (Figure S5A'). Following the same strategy, we re-clustered neurons based on the scores calculated only for the axon arbors that overlapped with the PVLP or PLP (Figure S5B).

Auditory Neuron Analysis

We employed a two-step search strategy to identify the pool of auditory interneurons for analysis. First, for 5 of the auditory types, we used the FlyCircuit neuron named by Lai et al. (2012) as the seed neuron for an initial search. Candidate neurons were selected using strict anatomical criteria. A second search was then done using these candidates as query neurons and collecting all high scoring neurons (mean NBLAST score >0.5) generating our set for analysis of types.

Sexually Dimorphic Neuron Analysis

Males have around 30 neurons mAL, females only 5; they regulate wing extension by males during courtship song (Koganezawa et al., 2010). The set of mAL neurons resulted from a search with a seed neuron, fru-M-500159. We then collected 41 hits with a mean NBLAST score greater than 0.2. Cluster analysis of the male neurons used partial skeletons that only contained the axonal and dendritic arbors (Figure 6C). We used this approach because all mAL neurons have a very similar primary neurite and main axon tract; differences in the position of these parts of the neuron are unlikely to be of great functional significance. In contrast differences in dendritic arborisations likely alter input connectivity including pheromone information detected by chemoreceptors on the male forelegs (Koganezawa et al., 2010; Kohl et al., 2015). The 3 different male types differed in the length of the ipsilateral ventral projection; this feature has previously been proposed as the basis of a qualitative classification of mAL neurons (Kimura et al., 2005).

There are around 20 P1 neurons that develop from the pMP-e *fruitless* neuroblast clone. They have extensive bilateral arborizations in the PVLP and ring neuropil, partially overlapping with the male-specific enlarged brain regions (MER) (Kimura et al., 2008; Cachero et al., 2010). The set of P1 neurons for analysis was identified by searching the FlyCircuit dataset with a tracing of the distinctive primary neurite of a pMP-e clone (Cachero et al.,

2010). Hierarchical clustering of the top hits, after manual verification, identified a subset consisting solely of P1 neurons which was used in the subsequent analysis.

NBLAST of Other Species

The 29 monarch butterfly neurons were downloaded from http://insectbraindb.org and scaled by a factor of 5 before clustering so that their length scale matched the adult *Drosophila* brains that we used. The 55 zebrafish mitral cells were provided by Dr Y. Yoshihara and S. Seung and colleagues. A bridging registration was constructed using CMTK to map opposite brain hemispheres onto each other. All neurons were then mapped onto the right-hand side of the larval fish brain before clustering.

Online resources

The image processing pipeline used two custom packages for the R statistical environment (http://www.r-project.org): https://github.com/jefferis/nat and https://github.com/jefferis/nat.as that coordinated processing by the low level registration (CMTK) and image processing (Fiji, unu) software mentioned above. NBLAST neuron search is implemented in R package **nat.nblast** available at https://github.com/jefferislab/nat.nblast. Analysis code specific to the flycircuit dataset is available in a dedicated R package https://github.com/jefferis/flycircuit, with a package vignette showcasing the main tools that we have developed. Further details of these supplemental software and the associated data are presented at http://jefferislab.org/si/nblast. The registered image dataset can be viewed in the stack viewer of the http://virtualflybrain.org website and all 16,129 registered single neuron images and corresponding skeletonized neurons will be available at http://jefferislab.org/si/nblast or on request to GSXEJ on a hard drive; the unregistered data remain available at http://flycircuit.tw.

Precomputed score matrices of all by all FlyCircuit neurons and all FlyCircuit neurons against all GMR GAL4 lines are distributed through the https://github.com/jefferis/flynblastscores R package or available via a remote web service that can return the top n hits for any neuron (see `vfb_nblast` function in package https://github.com/jefferis/vfbr).

The online resources accompanying this paper are listed at http://jefferislab.org/si/nblast and include the following:

- Code and instructions to generate some of the figure panels used in the paper. Instructions can be found at https://github.com/jefferislab/NBLAST_figures. A video demo is also available at http://youtu.be/LJgZejabqqg.
- The affinity propagation clustering of the flycircuit.tw dataset (as in Figure 7D), excluding intrinsic optic lobe neurons. This can be viewed online at http://jefferislab.org/si/nblast/clusters, including interactive 3D rendering of clusters powered by WebGL.
- The clusters identified by affinity propagation clustering (including intrinsic optic lobe neurons) are all indexed by the Virtual Fly Brain (http://www.virtualflybrain.org, VFB) website, which links to 3D WebGL renderings of each cluster hosted at jefferislab.org. Clusters can be identified by VFB queries for the neuropil region that they innervate. For example, search from the VFB homepage for “AMMC”. From the results page, choose the query “Images of neurons with: some part here (clustered by shape)”. A list of clusters, with thumbnail images is displayed; single exemplars are also displayed for each cluster, hyperlinked to the original data at flycircuit.tw. The images of the individual neurons that are part of this cluster can also be displayed in the stack browser from this page (“List individual members” or “Open all in viewer”). Clicking on a cluster thumbnail links to a page which includes a snapshot and 3D rendering of the cluster, and information about the neurons that are part of this cluster, including links to the appropriate Neuron ID pages at flycircuit.tw; a second table provides links to result pages for the most similar clusters. A video demo is available at http://youtu.be/YFsxjkdr5y8.

- An online web-app allowing on-the-fly NBLAST queries of FlyCircuit neurons against other FlyCircuit neurons, as well as queries of user-uploaded neurons against the FlyCircuit dataset, available at `http://jefferislab.org/si/nblast/on-the-fly/`.
- Video demos for the online resources are available at http://jefferislab.org/si/nblast/www/demos.

Supplemental Figures and Legends

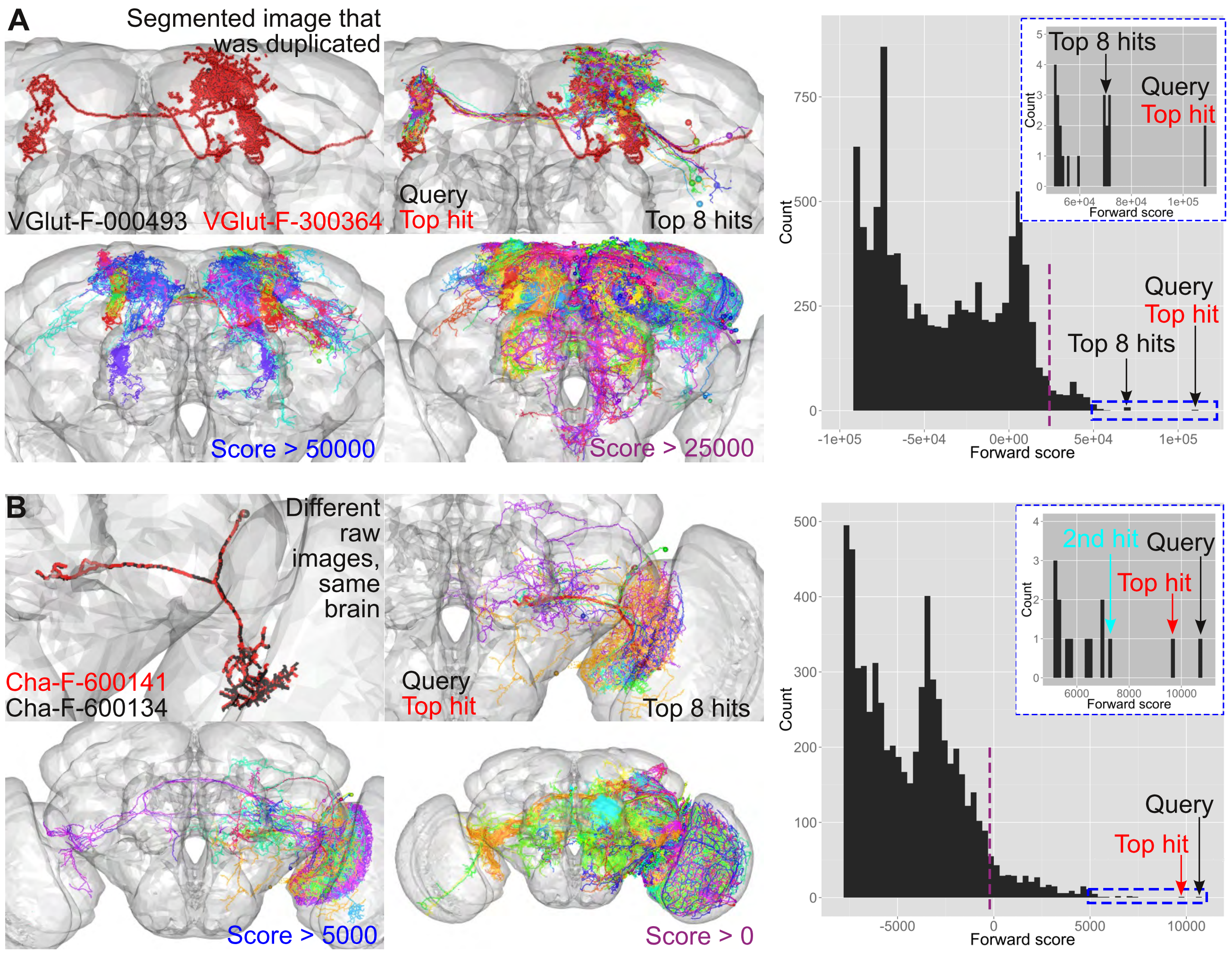

Figure S1: **Neuron search with NBLAST, related to Figure 3**
(A) NBLAST search with VGlut-F-000493 as query. Neuron plots of (from higher to lower score): the query (black) and top hit (red), top 8 hits, hits with a score over 50,000 and hits with a score over 25,000. The top hit corresponds to a segmented image that was duplicated. It perfectly overlays the query neuron. As the score decreases, so does the similarity of the hits to the query. On the right, histogram of forward scores. Only hits with scores over −100,000 are shown. The score of the query, top hit and top 8 hits are indicated. A dashed purple line marks 25,000. The left inset shows a zoomed view of the top hits (score > 50,000) (dashed blue rectangle in main plot). The score of the query, top hit and top 8 hits are indicated. **(B)** NBLAST search with Cha-F-600134 as query (black). Neuron plots of (from higher to lower score): the query and top hit, top 8 hits, hits with a score over 5,000 and hits with a score over 0. The top hit corresponds to an image of a neuron from the same brain but from a different raw image. It is very similar to the query neuron. As the score decreases, so does the similarity of the hits to the query. On the right, histogram of forward scores. Only hits with scores over −8,000 are shown. The score of the query and top hit are indicated. A dashed purple line marks 0. The left inset shows a zoomed view of the top hits (score > 5,000) (dashed blue rectangle in main plot). The score of the query, top hit and second top hits are indicated.

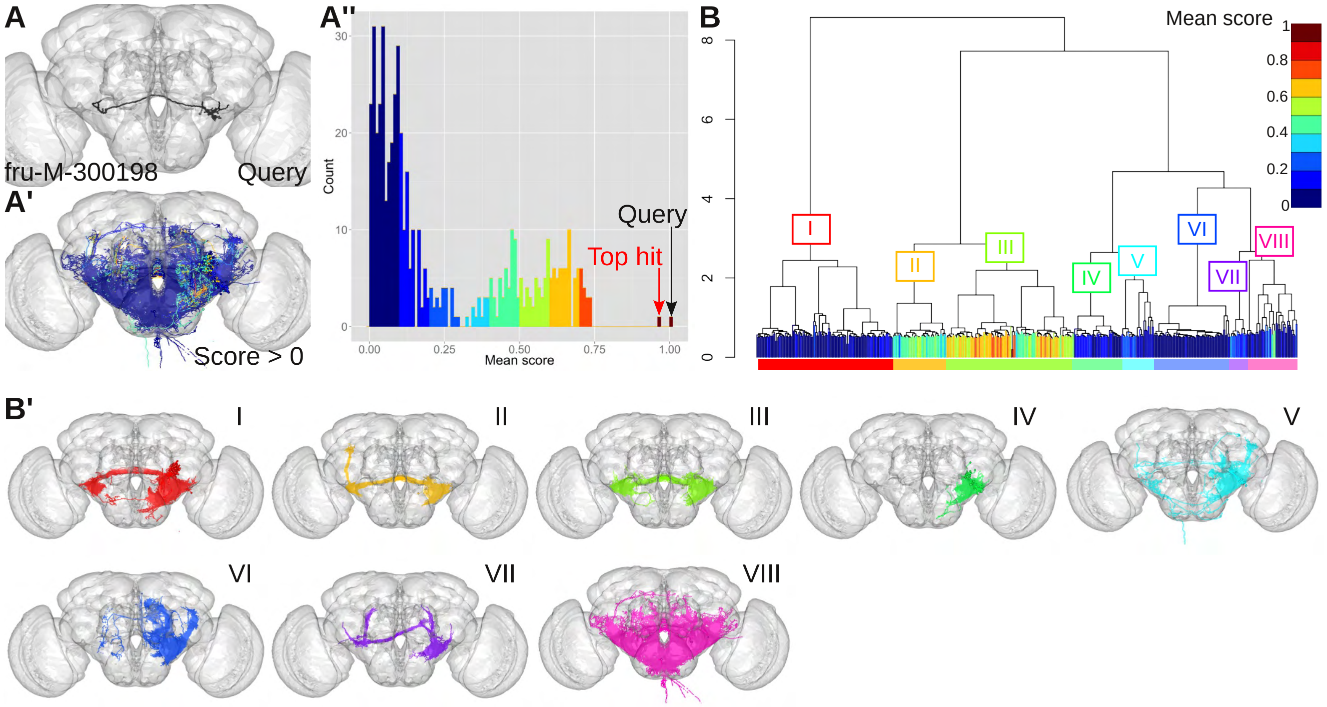

Figure S2: **NBLAST search using mean scores, related to Figure 3**
(A) The query neuron fru-M-300198 (same as in Figure 3B). **(A')** Neuron plot of the hits with a mean score over 0 for a search against the query. Hits are colored by score bin (10), as in A". **(A")** Histogram of mean scores for hits against fru-M-300198 with a score over 0 divided into 10 bins (indicated in the scale bar in B). **(B)** Hierarchical clustering of hits with a mean score over 0. The leaves of the dendrogram are colored by score (same as in A"), and as shown in the scale bar. The dendrogram was divided into eight groups (I–VIII), with each one being assigned a color, shown on the colored rectangle below the leaves. The query neuron is in group III, and the hits with the higher scores are in groups II and III. **(B')** Neuron plots corresponding to the dendrogram groups (I–VIII), following the colors assigned to each group. Groups II and III, corresponding to the highest scores, are the most similar neurons to the query.

Supplemental Kenyon cell analysis

Dataset collection and dividing into types We collected the dataset of initial KCs by performing a forward and reverse search against all neurons using one identified KC (fru-M-500225). We then selected neurons that had both raw scores above −2,500 (2,088 neurons). We performed affinity propagation clustering (Frey and Dueck, 2007) of these neurons, obtaining 59 clusters, and manually verified each one, resulting in 1,562 neurons being identified as KCs. An additional search for high scorers against these KC exemplars uncovered an extra 102 neurons, bringing the total number of KCs used in our analysis to 1,664, representing 10.3 % of the FlyCircuit dataset.

We performed hierarchical clustering of the KCs, based on the NBLAST scores, and divided the dendrogram into two groups (Figure 4A and Figure S3A). Contrary to expectations, one group contained both the γ and α′/β′ neurons, whereas the other group consisted exclusively of α/β neurons (Figure S3B–D), the largest subset in our sample. We separated α′/β′ from the γ neurons in a subsequent hierarchical clustering of this group. We performed additional analysis for each of the neuron types.

Analysis of γ neurons Hierarchical clustering of the 470 γ neurons resulted in a dendrogram which we divided into three groups (I–III) (Figure 4B'). The number of clusters was chosen by visual inspection in order to reveal differences in morphology and organization between the groups. Groups I and III corresponded to the classical γ neurons while group II matched atypical γ neurons. There were differences in neurite positioning in the calyx, from medial to lateral, with group I being the most medial, followed by groups II and III. There were also differences in the gamma lobe, with group II occupying the anterodorsal region, while groups I and III were mostly mixed in the rest of the lobe. A subsequent clustering analysis of the classical γ neurons divided into 4 groups (groups A-D) revealed that there were differences between the groups in their medial to lateral position in the calyx. These differences correlated to a certain degree with differences in the dorsal/ventral position of the projections in the γ lobe, with groups C and D, the most medial, being also the most dorsal (Figure 4B'). These observations suggest that the relative position of the projections of classical γ neurons is maintained at the calyx and γ lobe.

In order to understand if the relative position of the classical γ neurites was maintained in between the calyx and the γ lobe, we clustered the neurons based on the scores of the segments in the peduncle. We took neuron skeletons from classical γ neurons and isolated the axon arbors that co-localized with the peduncle volume (Figure S3E'). We then carried out a new clustering based on all-by-all NBLAST scores of these partial skeletons, cutting the dendrogram at a level defined by visual inspection (4 groups). The overall organization almost fully recapitulated the positioning of the neurites in the whole neuron analysis (compare Figure 4B' with Figure S3E'). A clear and expected lamination was found in the peduncle, with neurites occupying the most outer stratum. Differences in the medial to lateral positioning of neurites in the calyx followed the previously observed organization, with the most medial groups occupying the dorsal region of the gamma lobe. The overall organization almost fully recapitulated the positioning of the neurites in the whole neuron analysis. Thus, the stereotypical organization of the classical γ neurons is maintained throughout the neuropil.

Group II of the γ neurons matched atypical γ neurons (γ dorsal neurons) (Aso et al., 2014) with neurons that extended neurites posteriolaterally in the calyx and projected to the most dorsal region of the γ lobe (Figure 4B"). Hierarchical clustering of these neurons resulted in a dendrogram that we divided into 3 groups (a–c). This number of groups isolated the previously identified subtype of atypical γ neurons – γd neurons (Aso et al., 2009) – into one group (group a). These neurons extend neurites ventrolaterally at the level of the calyx (identified as ventral accessory calyx) (Aso et al., 2014). The other 2 groups (b, c) correspond to uncharacterized types. Although they project to a similar region in the γ lobe, their dendrites do not extend laterally and their calyx neurites are longer than γd neurons.

Analysis of α′/β′ neurons Hierarchical clustering analysis of the α′/β′ neurons and separation into 4 groups highlighted the characterized subtypes of α′/β′ neurons (Figure S3C). They differ in their anterior/posterior position in the peduncle and β′ lobe with three types described - α′/β′ anterior and posterior (α′/β′ap) and α′/β′ medial (α′/β′m)

(Tanaka et al., 2008, Y. Aso, personal communication)). Although we were unable to unambiguously assign a α′/β′ subtype to each group i–iv because our sample was too small, there were clear trends. Neurites of neurons in groups i and iv were more anterior than the other 2 groups (ii, iii) in both the peduncle and β′ lobe. These relative positions were not maintained in the calyx, with the two the anterior groups (i, iv) occupying either a medial or a lateral position.

Analysis of α/β neurons The largest subset of KCs corresponds to α/β neurons (Figure 4D). During the analysis of this group we found 18 neurons that did not correspond to α/β cells, since they innervated either only the β or the α lobes, and they were removed from the analysis. We performed hierarchical clustering on the remaining 1,091 cells and divided the resulting dendrogram into four groups (1–4) (Figure 4D'), which matched the four neuroblast lineages from which they originate (Zhu et al., 2003). The relative position of the neurites of the four groups within the calyx is somewhat maintained in the peduncle, with the lateral neuroblast clones (group 1 AL; group 2 PL) extending along the dorsolateral peduncle, while the medial clones (group 3 AM; group 4 PM) occupy a more ventromedial region. Hierarchical clustering of each group revealed an expected common organization for all neuroblast clones (Figure 4D" and Figure S3D'). For all groups there was a clear distinction between the late born core (α/β core, α/β-c) and early born peripheral neurons (α/β surface, α/β-s). Core neurons are on the inside stratum of the α lobe. They are also reported to occupy the inside stratum of the peduncle and β lobe (Tanaka et al., 2008). We were unable to observe this, although the projections of α/β core neurons were ventral to α/β surface neurons in both the peduncle and β lobe. There was also a trend for α/β surface neurons to occupy a more medial position in the calyx in comparison to α/β core ones. A subgroup of group 2 corresponded to the α/β posterior or pioneers neurons (α/βp). The α/βp neurons are the earliest born α/β and they innervate the accessory calyx, run along the surface of the posterior peduncle into the β lobe but stop before reaching the medial tip (Tanaka et al., 2008). A new clustering based on peduncle position of the neuron segments did not recapitulate the relative positions of the calyx neurites for each of the neuroblast clones observed in the whole neuron analysis suggesting that the relative position of the α/β neurons in the peduncle does not completely reflect their stereotypical organization in the calyx.

In order to investigate the stereotypical organization of α/β neurites, we performed a similar analysis as for the classic γ neurons, isolating the axon arbors that co-localized with the peduncle for groups 1 to 4 (Figure S3E"). The new clustering based on peduncle position of these partial neuron skeletons did not recapitulate the relative positions of the calyx neurites for each of the neuroblast clones observed in the whole neuron analysis (compare Figure 4D' with Figure S3E"). In addition, there was no clear organization of neurites in the α lobe that correlated with their position in the peduncle.

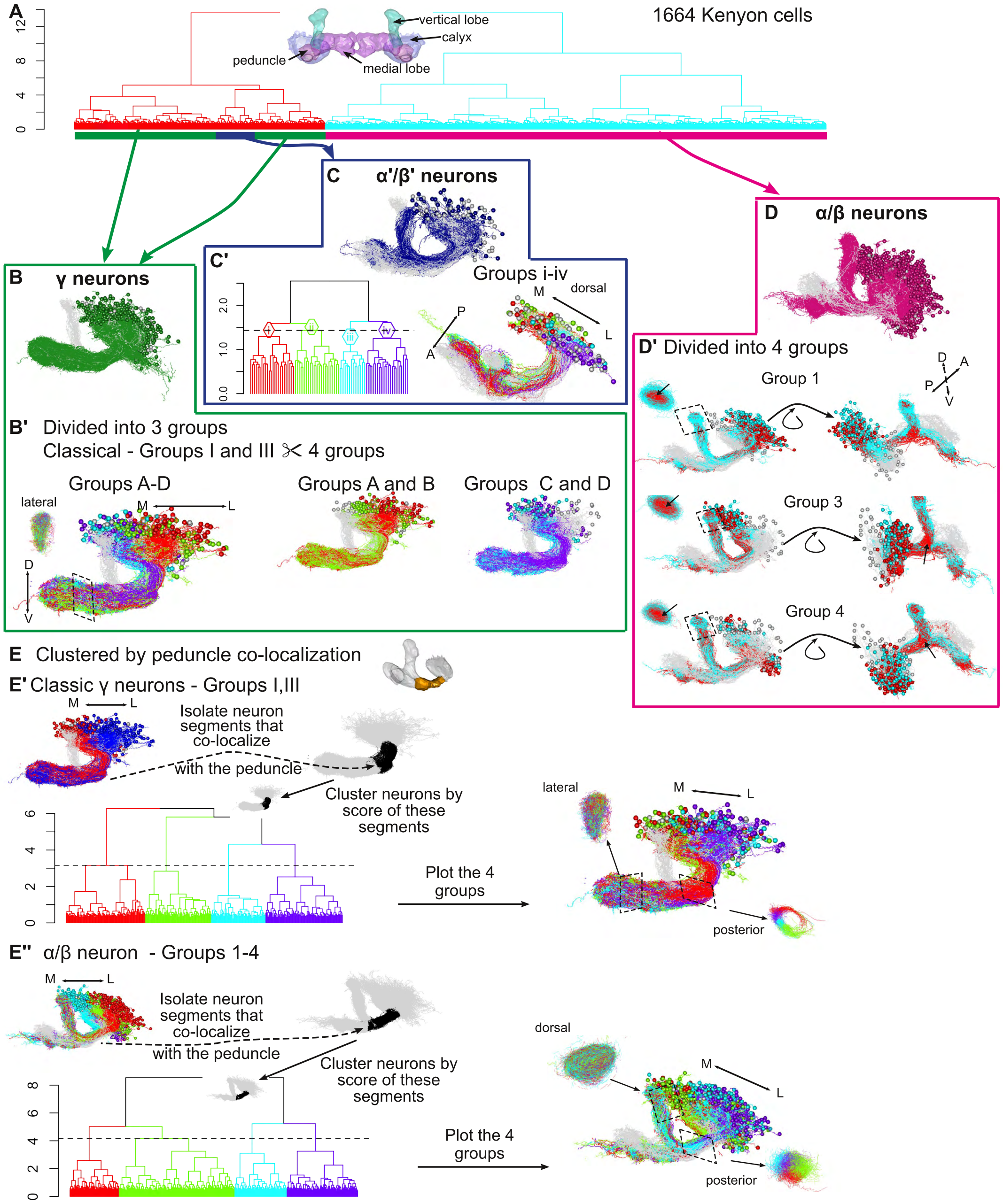

Figure S3: **NBLAST search and classification of hits reveals Kenyon cells subtypes, related to Figure 4**

(A) Hierarchical clustering (HC) of Kenyon cells (n=1664). Bars below the dendrogram indicate the γ (green), α′/β′ (blue) and α/β neurons (magenta), h=8.9. **(B)** Neuron plot of γ neurons. **(B')** HC of the classic γ neurons, corresponding to groups I and III in Figure 4B', divided into four groups (A–D). Neuron plots of groups A–D, A–B and C–D. **(C)** Neuron plot of α′/β′ neurons. **(C')** HC of α′/β′ neurons, divided into four groups (i–iv), h=1.43. **(D)** Neuron plot of α/β neurons. **(D')** α/β neurons divided into four groups (see Figure 4D') and neuron plots of groups 1, 3 and 4. Each group was divided divided into 2 subgroups, separating the neurons into surface (cyan) and core (red) (same as in Figure 4D"). Peripheral neurons occupied a more lateral calyx position and were dorsal to core neurons in the peduncle and β lobe. Lateral oblique, posterior oblique and a dorsal view of a peduncle slice (position indicated by dashed rectangle) are shown. **(E)** Reclustering of Kenyon cells based on the co-localization of neurons segments in the peduncle. The neuron segments that co-localize with the peduncle were isolated, followed by HC of the neurons based on the NBLAST score of the segments. **(E')** HC of the neurons segments of classic γ neurons, groups I and III (see B') divided into 4 groups, h=3.16. Neuron plot of the 4 groups. A posterior view of a slice of the peduncle shows an expected clear organization. It correlates to the position of the neurons in the calyx, with more medial neurons (cyan and green) being dorsal and ventral in the peduncle than more lateral neurons (red and purple). No clear structural organization is discernible in a lateral view of a slice of the γ lobe. **(E")** HC of the neurons segments of classic α/β neurons, groups 1 to 4 (see Figure 4D') divided into 4 groups, h=4.16. Neuron plot of the 4 groups. A posterior view of a peduncle slice shows an expected clear organization. It correlates to the position of the neurons in the calyx, with more medial neurons (cyan and green) being ventrolateral in the peduncle than more lateral neurons (red and purple). No structural organization is discernible in a dorsal view of a slice of the α lobe. For all neuron plots, the neurons in grey correspond to the Kenyon cell exemplars.

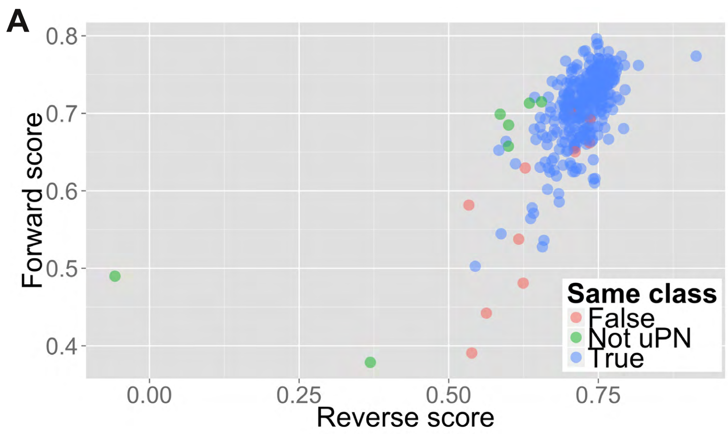

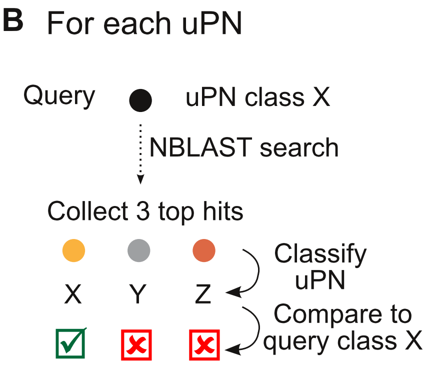

Matches to query class	%	n
Top hit	98.3	184
Any one	98.9	185
Any two	96.2	180
All three	95.2	178
		187

Figure S4: **NBLAST search and classification of hits reveals olfactory projection neuron neuronal types, related to Figure 4**
(A) Plot of the reverse and forward normalized scores for the top hit in an NBLAST search using the uniglomerular olfactory projection neurons (uPNs) as queries (n=327) against all other neurons. For each query neuron, we identified cases for which both the top hit and query were of the same class (True) (n=319); the top hit is a uPN but does not match the class of the query (False) (n=4), or the top hit is not a uPN (Not uPN) (n=4). **(B)** Percentage of neuron type matches in the top hit and top 3 hits for each uPN. The top three hits for each uPN (mean score) were collected and the neuron type of each hit and query was compared.

Dendrogram group	Neuron type	Comments
1	LC6	Lobula innervation, dorsal cell bodies, axons follow the anterior optic tract to the AOTU, turning ventrally midway to innervate the lateral PVLP.
2	LC9	As group 1, but terminating in the medial PVLP rather than extending laterally.
3	LC10B (possibly different subtype to 4)	Dorsal AOTU innervation, ventral to group 4.
4	LC10B (possibly different subtype to 3)	Dorsal AOTU innervation, dorsal to group 3.
5	New LC10 subtype	The most ventral AOTU innervation. Similar to group 7, but ventral to it in the AOTU.
6	LC10A	Axons project through ventral AOTU, turn sharply dorsally to terminate in the dorsal AOTU.
7	New LC10 subtype	Ventral AOTU innervation, dorsal to group 5.
A	LC12 subtype	Innervation in most lateral and anterior PVLP glomerulus. Note: corresponds to LC17 in Panser et al. (2015).
B	LC12 subtype	Innervation in more posterior and medial PVLP glomerulus than group A. Note: corresponds to LC12 in Panser et al. (2015).
C	LC4	Innervates a more medial PVLP glomerulus than LC12.
D	LT12	Tentative match. Class was identified in Otsuna and Ito (2006) based on a single neuron.
E	New LC class	Innervates more dorsal PVLP glomerulus than LC12. Extends along the posterior PVLP, with a sharp anterior turn. Terminates with a blunt-stick like ending in the lateral PVLP. Corresponds to LC18 in Panser et al. (2015).
F	New LT class	Similar to LT12, but with projections posterior to it, terminating in the lateral region of the superior posterior slope.
G	Unmatched	Do not correspond to a single type.

Table S1: Correspondences between hierarchical clustering groups of AOTU- and PVLP-innervating (groups 1-7) and PVLP- and PLP-innervating (groups A-G) uVPNs via NBLAST scores and previously determined neuron types (Otsuna and Ito, 2006). Related to Figure 5 and Figure S5.

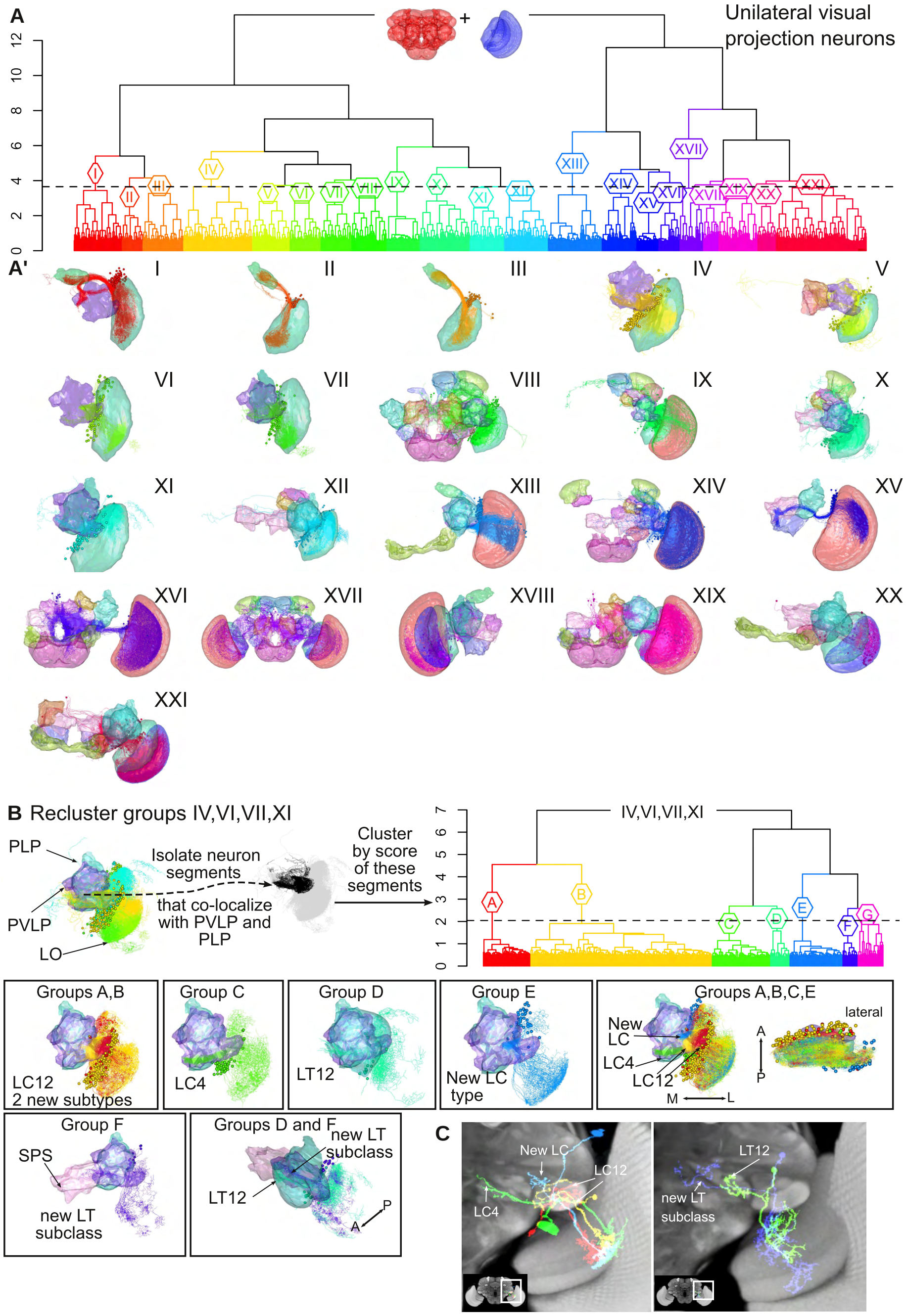

Figure S5: **NBLAST search and classification hits uncovers unilateral visual projection neurons neuronal types, related to Figure 5** **(A)** Hierarchical clustering (HC) analysis of unilateral visual projection neurons (uVPNs). The dendrogram was divided into 21 groups (I–XXI), h=3.65. Inset on the dendrogram shows the neuropils considered for the overlap. **(A')** Neuron plots of groups I to XXI. The neuropils that contain the most overlap are shown. **(B)** Reclustering of uVPN groups IV, VI, VII and XI from A. The neuron segments that co-localize with either the PVLP or PLP were isolated, followed by HC of the neurons based on the NBLAST score of these neuron segments. The dendrogram was divided into seven groups (A–G), h=2.04. Neuron plots matching the dendrogram groups to known uVPN types. An anterior, a lateral or lateral oblique views are shown. Groups A and B possibly correspond to two LC12 subtypes. Group B innervates a more anterior and medial glomeruli than group A (see also C). Group C corresponds to LC4. Group D corresponds to LT12 neurons. Group E corresponds to a new LC type. Group F corresponds to a possibly new LT subclass. This analysis identified 5 distinct types, including a new subtype. **(C)** Overlay of Z projections of registered image stacks of example neurons from the types identified in B on a partial Z projection of the template brain (a different one for each panel). The white rectangle on the inset shows the location of the zoomed in area. LC: lobula columnar neuron; LT: lobula tangential neuron. **(D)** Hierarchical clustering of intrinsic optic lobe neurons. This neuron set was defined as any neuron that overlapped only one of the optic lobes and with no arborization in the central brain neuropils. Dendrogram of the intrinsic optic lobe neurons, divided into into 20 groups (I–XX) with the corresponding heatmap calculated from the neuropil overlap in the different neuropils: medulla (ME), lobula (LO), lobula plate (LOP) and accessory medulla (AME). Neuron plots corresponding to the dendrogram groups are shown below. The neuropils for which the overlap is more significant are plotted. Although some organizational structure is seen, the dendrogram groups to do not represent unique types.

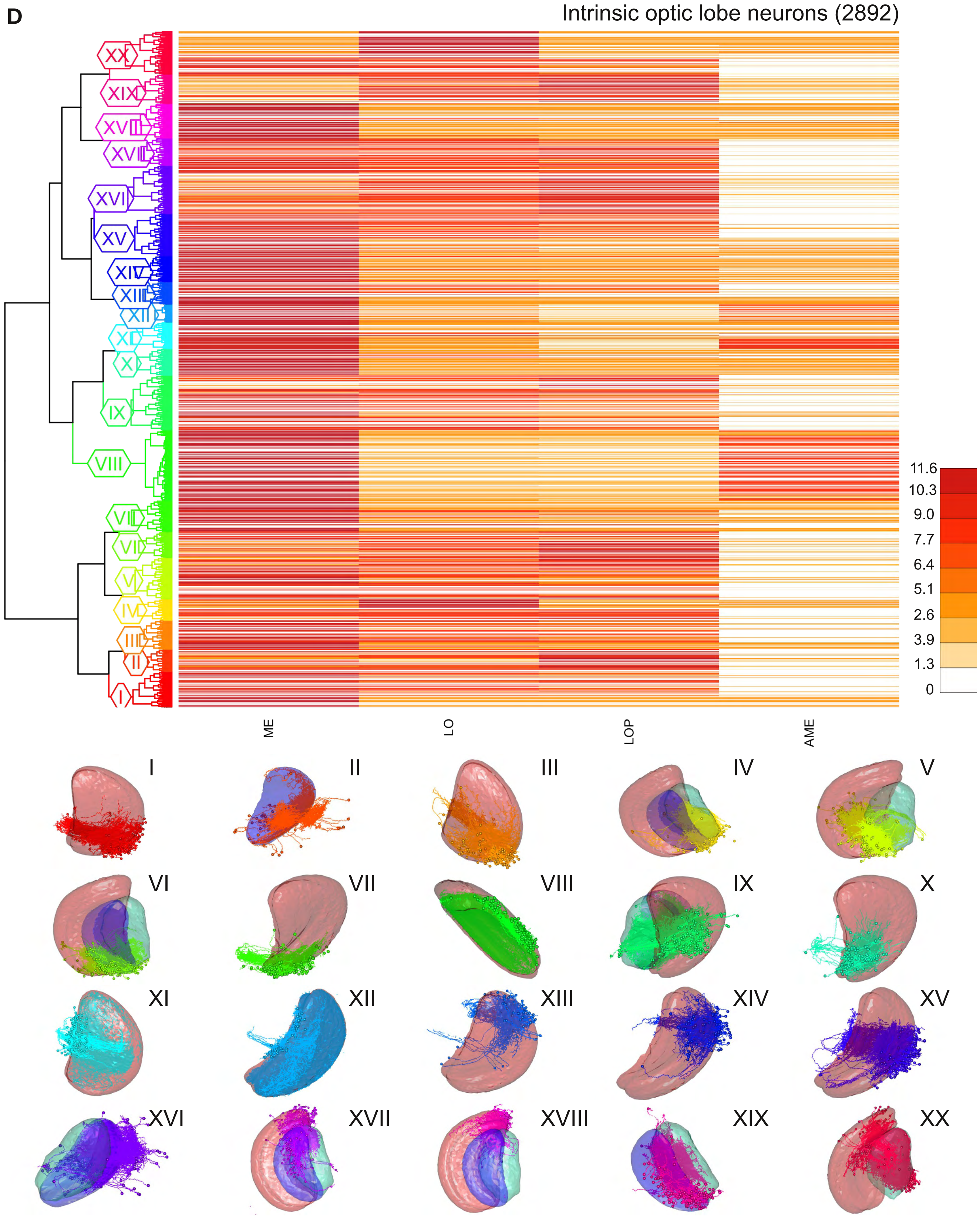
D
Intrinsic optic lobe neurons (2892)
XX
XIX
XVIII
XVII
XVI
XV
XIV
XIII
XII
XI
X
IX
VIII
VII
VI
V
IV
III
II
I
11.6
10.3
9.0
7.7
6.4
5.1
2.6
3.9
1.3
0
ME
LO
LOP
AME
I
II
III
IV
V
VI
VII
VIII
IX
X
XI
XII
XIII
XIV
XV
XVI
XVII
XVIII
XIX
XX

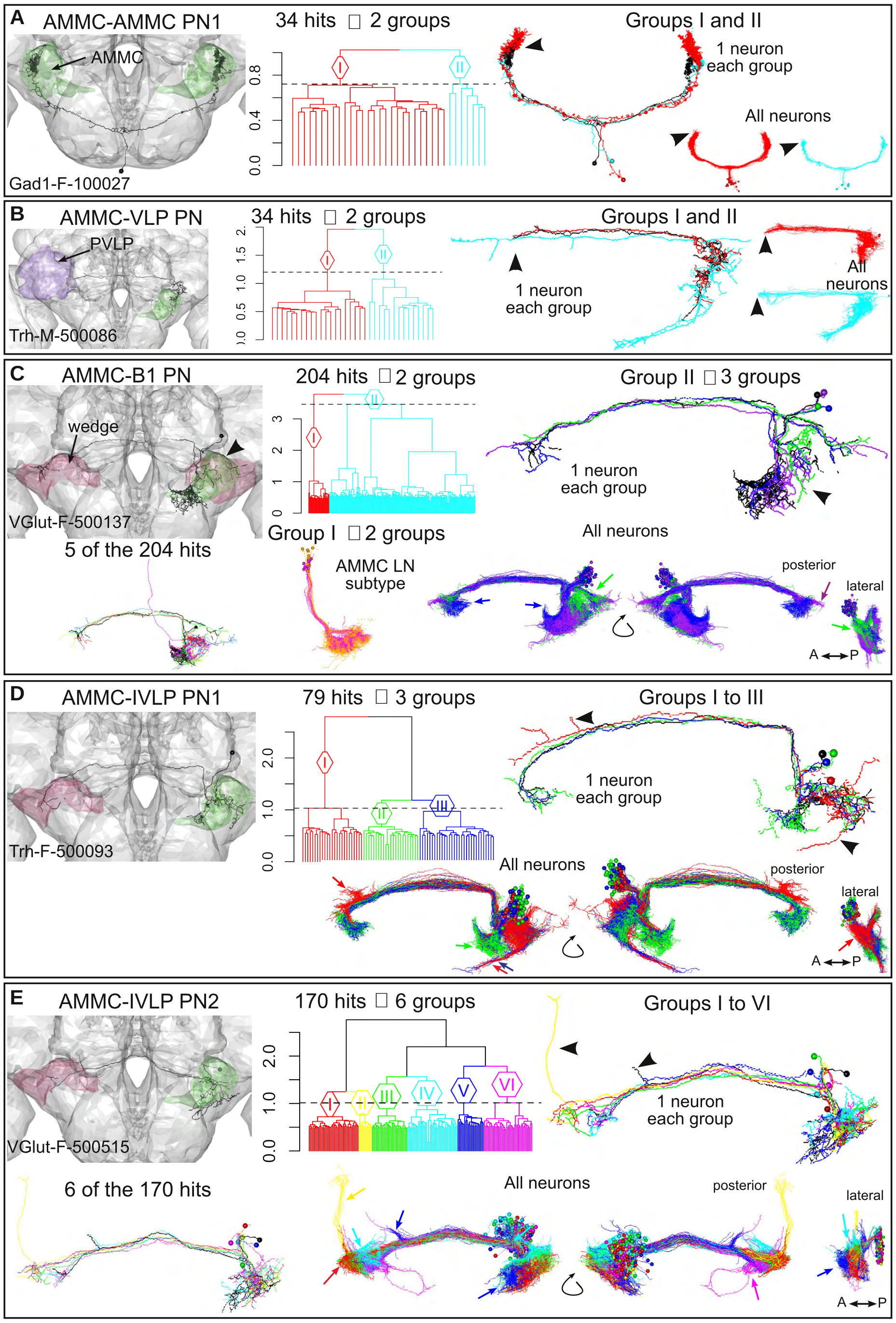

Figure S6: **NBLAST search and clustering identifies auditory neuron types, related to Figure 5**
On the left, a neuron of the types identified by Lai et al. (2012) (A-E) which was used as query. The dendrogram and neuron plots for each type (anterior, posterior and lateral views) showing either one or all neurons from each clustering group are shown on the middle and right panels. Main differences in arborization between neurons are indicated by arrowheads and arrows. **(A)** Hierarchical clustering (HC) of AMMC-AMMC projection neuron 1 (PN1). The 34 top scorers were clustered and divided into 2 groups, h=0.72. Neuron plot of the query neuron (black), and one neuron from each group (red and cyan). To the right, neuron plots of of each group. **(B)** HC of AMMC-VLP PNs. The 34 top scorers were clustered and divided into 2 groups, h=1.2. Neuron plot of the query neuron (black), and one neuron from each group (red and cyan). On the left, neuron plots of each group. **(C)** HC of AMMC-B1 PNs. Five hits of the 204 top scorers are shown on the left. The 204 top scorers were clustered and divided into 2 groups, h=3.46. Group I was matched to an unidentified type of AMMC local neurons (LN). It was clustered and divided into 2 groups, h=0.77. Group II corresponds to a mix of AMMC-B1 PNs and AMMC-IVLP PN1. After selecting the AMMC-B1 PNs, the neurons were clustered and divided into 3 groups, h=1.5. Neuron plot of the query neuron (black), and one neuron from each group (purple and green). Below, neuron plots of the 3 groups. **(D)** HC of AMMC-IVLP PN1. The 79 top scorers were clustered and divided into 3 groups, h=1.03. Neuron plot of the query neuron (black), and one neuron from each group (red, green and blue). Below, anterior, posterior and lateral view neuron plots of the 3 groups. **(E)** HC of AMMC-IVLP PN2. Six hits of the 170 top scorers are shown on the left. The 170 top scorers were clustered and divided into 6 groups, h=1.02. Neuron plot of the query neuron (black), and one neuron from each group (red, yellow, green, cyan, blue, magenta). Below, anterior, posterior and lateral view neuron plots of the 6 groups. Arrows and arrowheads indicate differences between groups. wedge in magenta, AMMC in green or PVLP in purple.

Panel	Neuron type	Comments
A	AMMC-AMMC PN1 (2 possible subtypes)	These neurons innervate both AMMCs, with a ventral cell body. Group I extends more dorsally than group II.
B	AMMC-VLP (2 possible subtypes)	These neurons innervate the ipsilateral AMMC and the contralateral VLP. Group II extends more laterally than group I.
C	AMMC-B1 PN (3 possible subtypes)	These neurons innervate the ipsilateral AMMC and IVLP and the contralateral IVLP. Blue group innervates more medial regions and has more extensive innervation contralaterally; purple group innervates more anterior and posterior regions ipsilaterally, and the green group innervates the dorsal regions in the ipsilateral AMMC.
C	New AMMC-LN type (2 subtypes)	A type of AMMC LN, with a dorsal cell body. Two possible subtypes: the magenta group innervates more dorsal regions than the orange group.
D	AMMC-IVLP PN1 (3 possible subtypes)	These neurons innervate the ipsilateral AMMC and the contralateral IVLP. Red group has a more dorsomedial ipsilateral innervation more medial regions, with some dorsal medial branches in the contralateral hemisphere; some neurons extend a long neurite ventrally ipsilaterally. The green group innervates the more ventromedial regions ipsilaterally. The blue group is similar to the green one, although it does not extend as ventrally in the ipsilateral side, and a few neurons extend a long neurite ventrally (similar to red group).
E	AMMC-IVLP PN2 (6 possible subtypes)	These neurons innervate the ipsilateral AMMC and the contralateral IVLP, with a posterior cell body. Group I innervates the more lateral regions in both hemispheres. Group II has a long dorsal branch in the contralateral hemisphere, at the lateral edge of the neuron. Group III and IV are very similar, with the latter innervating more dorsal regions in both hemispheres. Group V corresponds to the strict definition of the neuron type by Lai et al. (2012), showing a short dorsal branch just medial to the contralateral IVLP. Group VI are similar to group V, with a few neurons showing a short dorsal branch, and innervating a more ventral region in the contralateral IVLP.

Table S2: Correspondence between hierarchical clustering of auditory neuron via NBLAST scores and previously determined neuron types (Lai et al., 2012). Related to Figure 5 and Figure S6.

Supplemental Movies

Movie S1: Online NBLAST search, related to Figure 2.

This movie describes how to use the NBLAST web app to search for similar neurons, within the FlyCircuit dataset.

Movie S2: Online NBLAST search for GAL4 drivers, related to Figure 2.

This movie describes how to use the NBLAST web app to search for GAL4 drivers of the FlyLight collection that might label a specified neuron.

Movie S3: Online NBLAST search for neurons using a trace, related to Figure 2.

This movie describes how to use the NBLAST web app to search for FlyCircuit neurons that are similar to an uploaded trace.

Supplemental References

Aso, Y., Grübel, K., Busch, S., Friedrich, A.B., Siwanowicz, I., and Tanimoto, H. (2009). The mushroom body of adult Drosophila characterized by GAL4 drivers. Journal of neurogenetics *23*, 156–172.

Aso, Y., Hattori, D., Yu, Y., Johnston, R.M., Iyer, N.A., Ngo, T.T., Dionne, H., Abbott, L., Axel, R., Tanimoto, H., et al. (2014). The neuronal architecture of the mushroom body provides a logic for associative learning. eLife *3*, e04577.

Ballard, D.H. (1981). Generalizing the Hough transform to detect arbitrary shapes. Pattern Recognition *13*, 111–122.

Basu, S., Condron, B., and Acton, S.T. (2011). Path2Path: hierarchical Path-Based analysis for neuron matching. In Biomedical Imaging: From Nano to Macro, 2011 IEEE International Symposium on (IEEE), pp. 996–999.

Cachero, S., Ostrovsky, A.D., Yu, J.Y., Dickson, B.J., and Jefferis, G.S.X.E. (2010). Sexual dimorphism in the fly brain. Curr Biol *20*, 1589–601.

Cardona, A., Saalfeld, S., Arganda, I., Pereanu, W., Schindelin, J., and Hartenstein, V. (2010). Identifying neuronal lineages of Drosophila by sequence analysis of axon tracts. J Neurosci *30*, 7538–53.

Chiang, A.S., Lin, C.Y., Chuang, C.C., Chang, H.M., Hsieh, C.H., Yeh, C.W., Shih, C.T., Wu, J.J., Wang, G.T., Chen, Y.C., Wu, C.C., Chen, G.Y., Ching, Y.T., Lee, P.C., Lin, C.Y., Lin, H.H., Wu, C.C., Hsu, H.W., Huang, Y.A., Chen, J.Y., Chiang, H.J., Lu, C.F., Ni, R.F., Yeh, C.Y., and Hwang, J.K. (2011). Three-dimensional reconstruction of brain-wide wiring networks in Drosophila at single-cell resolution. Curr Biol *21*, 1–11.

Conte, D., Foggia, P., Sansone, C., and Vento, M. (2004). Thirty years of graph matching in pattern recognition. International journal of pattern recognition and artificial intelligence *18*, 265–298.

Doube, M., Kłosowski, M.M., Arganda-Carreras, I., Cordelières, F.P., Dougherty, R.P., Jackson, J.S., Schmid, B., Hutchinson, J.R., and Shefelbine, S.J. (2010). BoneJ: Free and extensible bone image analysis in ImageJ. Bone *47*, 1076 – 1079.

Evers, J.F., Schmitt, S., Sibila, M., and Duch, C. (2005). Progress in functional neuroanatomy: precise automatic geometric reconstruction of neuronal morphology from confocal image stacks. J Neurophysiol *93*, 2331–42.

Frey, B.J., and Dueck, D. (2007). Clustering by passing messages between data points. Science *315*, 972–976.

Ganglberger, F., Schulze, F., Tirian, L., Novikov, A., Dickson, B., Bühler, K., and Langs, G. (2014). Structure-Based Neuron Retrieval Across Drosophila Brains. Neuroinformatics .

Ito, K., Shinomiya, K., Ito, M., Armstrong, J.D., Boyan, G., Hartenstein, V., Harzsch, S., Heisenberg, M., Homberg, U., Jenett, A., et al. (2014). A systematic nomenclature for the insect brain. Neuron *81*, 755–765.

Jefferis, G.S.X.E., Potter, C.J., Chan, A.M., Marin, E.C., Rohlfing, T., Maurer, C.R.J., and Luo, L. (2007). Comprehensive maps of Drosophila higher olfactory centers: spatially segregated fruit and pheromone representation. Cell *128*, 1187–1203.

Jenett, A., Rubin, G.M., Ngo, T.T., Shepherd, D., Murphy, C., Dionne, H., Pfeiffer, B.D., Cavallaro, A., Hall, D., Jeter, J., et al. (2012). A GAL4-Driver Line Resource for Drosophila Neurobiology. Cell Rep *2*, 991–1001.

Khoshelham, K. (2007). Extending Generalized Hough Transform to Detect 3D Objects in Laser Range Data. In ISPRS Workshop on Laser Scanning, Proceedings, LS 2007. pp. 206–210.

Kimura, K.i., Hachiya, T., Koganezawa, M., Tazawa, T., and Yamamoto, D. (2008). Fruitless and doublesex coordinate to generate male-specific neurons that can initiate courtship. Neuron *59*, 759–769.

Kimura, K.I., Ote, M., Tazawa, T., and Yamamoto, D. (2005). Fruitless specifies sexually dimorphic neural circuitry in the Drosophila brain. Nature *438*, 229–233.

Koganezawa, M., Haba, D., Matsuo, T., and Yamamoto, D. (2010). The Shaping of Male Courtship Posture by Lateralized Gustatory Inputs to Male-Specific Interneurons. Current Biology *20*, 1–8.

Kohl, J., Huoviala, P., and Jefferis, G.S. (2015). Pheromone processing in Drosophila. Curr Opin Neurobiol *34*, 149–157.

Lai, J.S.Y., Lo, S.J., Dickson, B.J., and Chiang, A.S. (2012). Auditory circuit in the Drosophila brain. Proc Natl Acad Sci U S A *109*, 2607–12.

Le, Q., Ranzato, M., Monga, R., Devin, M., Chen, K., Corrado, G., Dean, J., and Ng, A. (2012). Building high-level features using large scale unsupervised learning. In International Conference in Machine Learning.

Manton, J.D., Ostrovsky, A.D., Goetz, L., Costa, M., Rohlfing, T., and Jefferis, G.S.X.E. (2014). Combining genome-scale Drosophila 3D neuroanatomical data by bridging template brains. bioRxiv .

Masse, N.Y., Cachero, S., Ostrovsky, A., and Jefferis, G.S.X.E. (2012). A mutual information approach to automate identification of neuronal clusters in Drosophila brain images. Frontiers in Neuroinformatics *6*.

Mayerich, D., Bjornsson, C., Taylor, J., and Roysam, B. (2012). NetMets: software for quantifying and visualizing errors in biological network segmentation. BMC Bioinformatics *13 Suppl 8*, S7.

Otsuna, H., and Ito, K. (2006). Systematic analysis of the visual projection neurons of Drosophila melanogaster. I. Lobula-specific pathways. J Comp Neurol *497*, 928–958.

Panser, K., Tirian, L., Schulze, F., Villalba, S., Jefferis, G.S., Buehler, K., and Straw, A.D. (2015). Automatic segmentation of Drosophila neural compartments using GAL4 expression data reveals novel visual pathways. bioRxiv .

Peng, H., Tang, J., Xiao, H., Bria, A., Zhou, J., Butler, V., Zhou, Z., Gonzalez-Bellido, P.T., Oh, S.W., Chen, J., Mitra, A., Tsien, R.W., Zeng, H., Ascoli, G.A., Iannello, G., Hawrylycz, M., Myers, E., and Long, F. (2014). Virtual finger boosts three-dimensional imaging and microsurgery as well as terabyte volume image visualization and analysis. Nat Commun *5*, 4342.

Rohlfing, T., and Maurer, C. R., J. (2003). Nonrigid image registration in shared-memory multiprocessor environments with application to brains, breasts, and bees. IEEE Trans Inf Technol Biomed *7*, 16–25.

Rueckert, D., Sonoda, L.I., Hayes, C., Hill, D.L., Leach, M.O., and Hawkes, D.J. (1999). Nonrigid registration using free-form deformations: application to breast MR images. IEEE Trans Med Imaging *18*, 712–21.

Tanaka, N.K., Endo, K., and Ito, K. (2012). Organization of antennal lobe-associated neurons in adult Drosophila melanogaster brain. J Comp Neurol *520*, 4067–4130.

Tanaka, N.K., Tanimoto, H., and Ito, K. (2008). Neuronal assemblies of the Drosophila mushroom body. J Comp Neurol *508*, 711–755.

Wan, Y., Long, F., Qu, L., Xiao, H., Hawrylycz, M., Myers, E.W., and Peng, H. (2015). BlastNeuron for Automated Comparison, Retrieval and Clustering of 3D Neuron Morphologies. Neuroinformatics .

Yu, J.Y., Kanai, M.I., Demir, E., Jefferis, G.S.X.E., and Dickson, B.J. (2010). Cellular organization of the neural circuit that drives Drosophila courtship behavior. Curr Biol *20*, 1602–14.

Zhu, S., Chiang, A.S., and Lee, T. (2003). Development of the Drosophila mushroom bodies: elaboration, remodeling and spatial organization of dendrites in the calyx. Development *130*, 2603–2610.
